# Semisynthesis of Isomerized Histone H4 Reveals Robustness and Vulnerability of Chromatin toward Molecular Aging

**DOI:** 10.1021/jacs.4c14136

**Published:** 2025-02-03

**Authors:** Tianze Zhang, Luis F. Guerra, Yana Berlina, Jon R. Wilson, Beat Fierz, Manuel M. Müller

**Affiliations:** † Department of Chemistry, King’s College London, Britannia House, 7 Trinity Street, London SE1 1DB, U.K.; ‡ École Polytechnique Fédérale de Lausanne (EPFL), ISIC, Lausanne CH-1015, Switzerland; § The Francis Crick Institute, 1 Midland Road, London NW1 1AT, U.K.

## Abstract

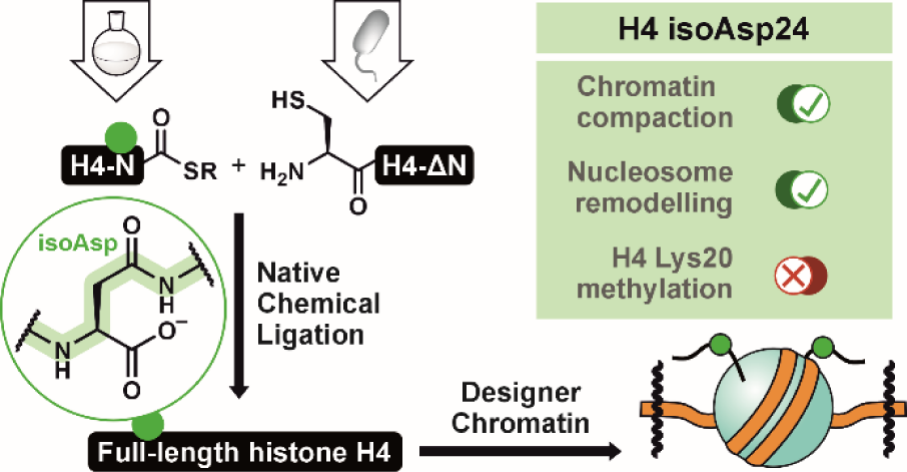

Proteins are subject to aging in the form of spontaneous, nonenzymatic post-translational modifications (PTMs). One such PTM is the formation of the β-linked isomer l-isoaspartic acid (isoAsp) from aspartic acid (Asp) or asparagine residues, which tends to occur in long-lived proteins. Histones can exhibit half-lives on the order of 100 days, and unsurprisingly, isoAsp formation has been observed in nearly every histone family. Delineating the molecular consequences of isoAsp formation in histones is challenging due to the multitude of processes that occur on such time scales. To isolate the effects of a specific isoAsp modification thus necessitates precise *in vitro* characterization with well-defined substrates. Here, we adapt a protein semisynthesis approach to generate full-length variants of histone H4 in which the canonical Asp at position 24 is replaced by its isoAsp isomer (H4isoD24). This variant was incorporated into chromatin templates, and the resulting constructs were used to interrogate key parameters of chromatin integrity and maintenance *in vitro*: compaction, nucleosome remodeling, and methylation of H4 lysine 20 (H4K20). Remarkably, despite its disruptive changes to the backbone’s spacing and direction, isoD24 did not dramatically disrupt Mg^2+^-mediated chromatin self-association or nucleosome repositioning by the remodeler Chd1. In contrast, H4isoD24 significantly inhibited both Set8- and Suv4-20h1-catalyzed methylation at H4K20. These results suggest that H4isoD24 gives rise to a complex reorganization of the chromatin functional landscape, in which macroscopic processes show robustness and local mechanisms exhibit vulnerability to the presence of this mark.

## Introduction

Biomacromolecules, including proteins, are subject to molecular “wear-and-tear” as they undergo spontaneous chemical modification.^1^ Most proteins are regularly replaced to prevent the accumulation of damaged species. Indeed, typical half-lives of proteins range from 0.5–43 h in mammalian cells, much shorter than the lifetimes of the host organisms. Some proteins, however, are exceptionally long-lived and constitute persistent targets of inevitable molecular damage.^2^ Histones, for example, exhibit cell- and tissue-specific half-lives on the order of 100 days across several studies.^3−7^ Given the importance of histones as both genomic scaffolds and as targets of a vast, regulatory “code” of post-translational modifications (PTMs),^8^ the predisposition of this class of proteins to aging-related modifications presents a fundamental challenge for the maintenance of cellular homeostasis.

**Figure 1:**
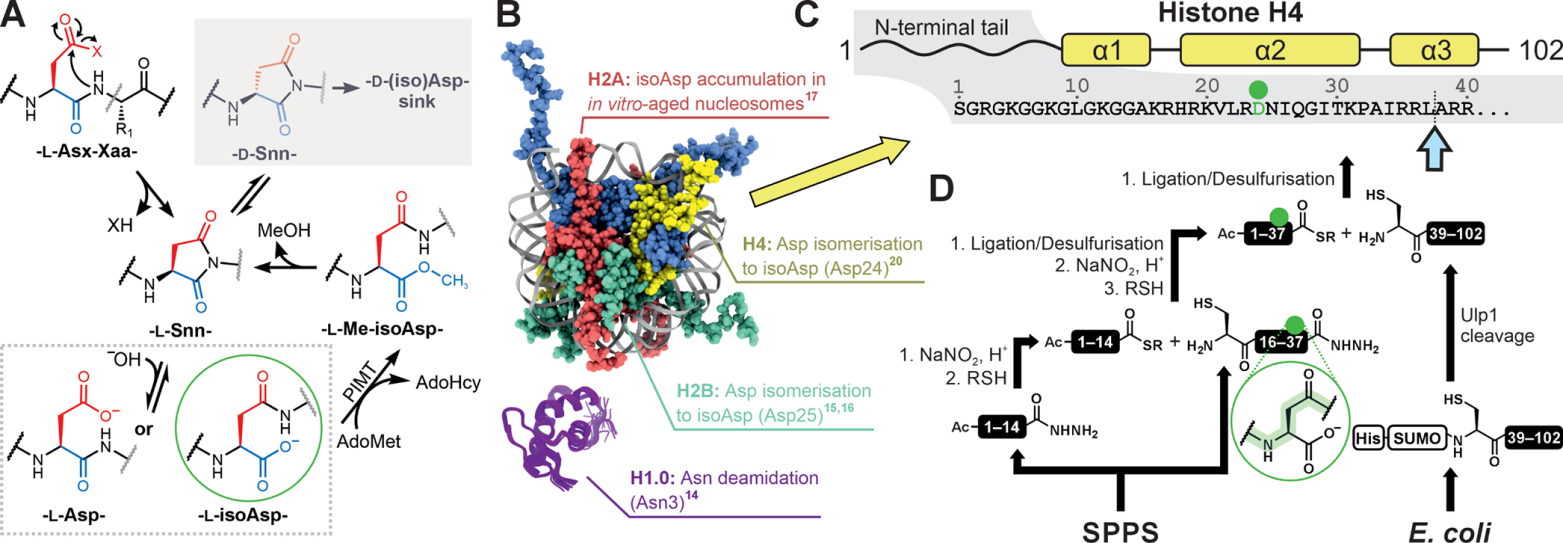
isoAsp formation in histone H4 and its investigation by protein semisynthesis. (A) isoAsp is formed from Asx residues via spontaneous cyclization to and biased hydrolysis of a succinimide intermediate. *In vivo* repair occurs through methylation of isoAsp by PIMT, though this process falters in the case of epimerization of the succinimide and subsequent generation of d-(iso)Asp. PIMT = protein isoaspartyl methyltransferase; AdoMet = *S*-adenosyl-l-methionine; AdoHcy = *S*-adenosyl-l-homocysteine. (B) Documented occurrences of the Asx deamidation/isomerization pathway in histones H1.0 (purple; PDB: 6HQ1), H2A (red), H2B (cyan), and H4 (yellow). Nucleosome structure from PDB: 1KX5.^29^ (C) Architecture of H4, indicating the α-helices of the histone fold, the sequence of the N-terminal tail, and the sites of isoAsp formation (green circle) and ligation (dashed line and blue arrow). (D) Scheme for the semisynthesis of full-length H4isoD24 via a ligation/desulfurization strategy. The site of truncation for recombinant H4 is indicated by a blue arrow. isoAsp is highlighted by a green circle. SPPS = solid-phase peptide synthesis; His = hexahistidine tag; SUMO = small ubiquitin-like modifier.

Asparagine (Asn) and aspartate (Asp) residues are particularly prone to aging-related modifications. They spontaneously rearrange into a β-linked isoform, isoaspartate (isoAsp), via the formation of a 5-membered succinimide intermediate that can be hydrolyzed to yield either an isoAsp or Asp residue (Figure 1A).^9^ The time scale of isoAsp formation varies by several orders of magnitude, depending on the identity of the original amino acid, its surrounding sequence, and the presence of local secondary structure—typically ranging from days to centuries for Asn,^10^ with 13–36x slower rates for Asp.^11^ Glycine and, to a lesser extent, small polar residues in the i+1 position are typically associated with rapid isoAsp formation. Conversely, the absence of an N+1 backbone N–H in proline, or the masking of this group’s reactivity via H-bonding (e.g., in the context of secondary structure), protects the Asx residue from succinimide formation. isoAsp can be repaired *in vivo* through the action of the enzyme protein isoaspartyl methyltransferase (PIMT).^12^ Methylesterification of l-isoAsp by PIMT facilitates reformation of the succinimide intermediate, enabling downstream hydrolysis into a biased mixture of 15–30% Asp and 70–85% isoAsp.^13^ However, this repair process is fundamentally limited by its underlying reaction pathway. The succinimide intermediate is prone to epimerization, leading to the formation of the ineffective PIMT substrates d-Asp and d-isoAsp and thus their gradual accumulation.^9^

Products of the above Asn/Asp degradation and repair pathways, including isoAsp, have been observed in all histone families excluding histone H3 (Figure 1B); the latter absence is presumably due to the sequence and structural context of Asn/Asp residues. Linker histone H1.0 undergoes deamidation at Asn3–Ser4, which—alongside concurrent N-terminal acetylation—was postulated to influence chromatin structure by weakening electrostatically mediated histone-DNA contacts.^14^ isoAsp reaches near-stoichiometric levels in histone H2B extracted from the brains of PIMT-knockout mice,^15^ and specifically Asp25 of H2B undergoes significant *in vivo* racemization via the PIMT-dependent repair pathway^16^ and accumulates isoAsp in mononucleosomes derived from rat liver and subjected to *in vitro* aging.^17^ Mononucleosomes isolated from chicken erythrocytes and subjected to the same aging process instead exhibited isoAsp formation primarily in histone H2A, an observation explained by differences in the two species’ histone sequences. The presence of isoAsp25 in H2B(21–35) peptides renders them immunogenic and may thus provide an explanation for the targeting of H2B by autoantibodies in systemic lupus erythematosus.^18^d-isoAsp25 in H2B is also significantly enriched in transcriptionally active genes, suggesting that either structurally open chromatin is more permissive for isoAsp formation in H2B or, conversely, that this modification increases chromatin accessibility.^19^ PIMT-mediated methylation of Asp24 in histone H4 (H4D24) was found in histone extracts prepared from mouse livers, indicating a significant presence of isoAsp at this position (H4isoD24).^20^ Intriguingly, methylation of this residue also increased binding to VprBP, a substrate recognition module of the DDB1-CUL4 E3 ligase complex, hinting at a connection between protein aging, damage, and degradation.

Despite these findings, the molecular consequences of isoAsp formation in histones remain unclear. Illuminating the direct mechanisms by which isoAsp impacts chromatin structure and function necessitates controlled biochemical and biophysical studies *in vitro* with chemically well-defined histones containing this modification, but such substrates are difficult to generate due to the slow rates of isoAsp formation and the heterogeneity of protein aging pathways more generally. Here we deploy a chemical biology approach based on protein semisynthesis—which is ideally suited to produce such site-specifically modified histones^21^ and fully compatible with the incorporation of isoAsp residues^22^—to assess the possible impacts of the spontaneous H4isoD24 modification. We prepared H4 bearing this aging-related mark via protein semisynthesis (Figures 1C,D) and incorporated it into chromatin templates in order to ascertain the effects of H4 aging in a physiologically relevant molecular context—packaged alongside its histone partners H2A, H2B, and H3 within well-folded nucleosomes.^23^ We specifically chose the H4isoD24 modification as a model for the exploration of histone aging due to the importance of the H4 N-terminal tail in controlling chromatin structure and function. The highly basic H4 tail is necessary for chromatin compaction and facilitates this folding via internucleosomal contacts with a seven-residue acidic patch at the H2A/H2B dimer interface.^24,25^ The H4 tail also serves as the main competitor for other binders of the H2A/H2B acidic patch.^26^ Similarly, many nucleosome-targeted enzymes—e.g., nucleosome remodelers^27^—engage the basic residues of the H4 tail via their own acidic interfaces. H4D24 is also adjacent to key regulatory PTMs, e.g., H4K20 methylation.^28^ Thus, we utilized our full-length H4isoD24 constructs to investigate the effects of this modification on the following parameters of chromatin integrity and maintenance: chromatin compaction, nucleosome remodeling, and the deposition of nearby signaling PTMs. Our results demonstrate that the extent of chromatin’s robustness or vulnerability toward the aging of H4 via formation of H4isoD24 is highly specific to the chromatin-centered activity being interrogated. The variability of this response suggests that molecular aging may induce a complex reorganization of global chromatin function, with higher-order molecular interactions unaffected and local enzymatic engagement substantially altered.

## Results

### Semisynthesis of H4isoD24

**Figure 2:**
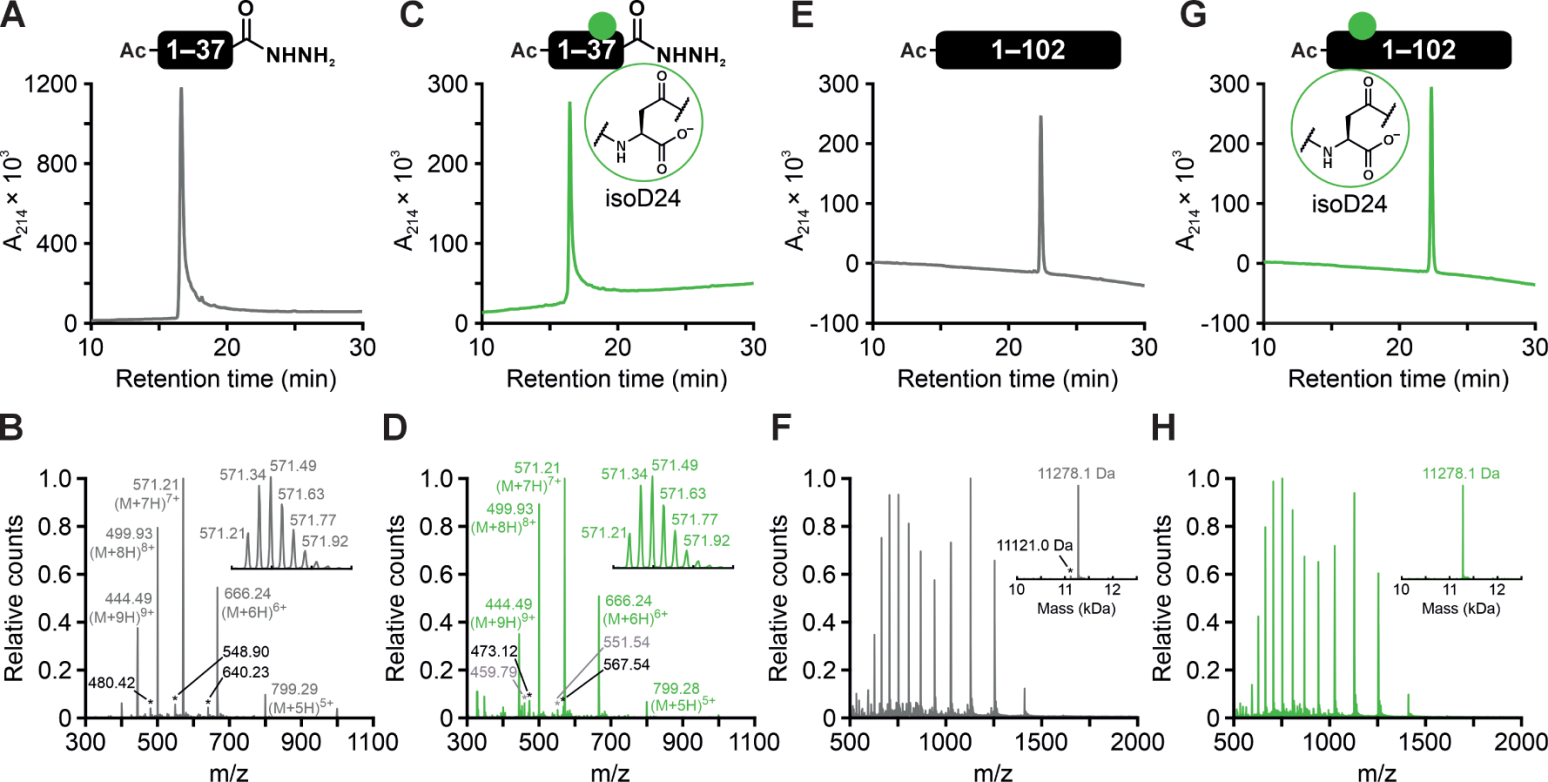
Unmodified and isoAsp-containing H4 peptides and full-length proteins. (A) RP-HPLC and (B) HRMS of H4D24(1–37). Expected mass = 3991.38 Da; observed mass = 3991.47 Da. Asterisks denote 8+, 7+, and 6+ charge states of a -Arg truncation. (C) RP-HPLC and (D) HRMS of H4isoD24(1–37). Expected mass = 3991.38 Da; observed mass = 3991.47 Da. Gray asterisks denote 6+ and 5+ charge states of H4isoD24(A15C; 15–37) with loss of hydrazine, and black asterisks denote 6+ and 5+ charge states of H4isoD24(A15C; 15–37) with oxidation of C15 to the sulfonic acid. (E) RP-HPLC and (F) HRMS of full-length H4D24. Expected mass = 11278.2 Da; observed mass = 11278.1 Da. Asterisk denotes a -Arg truncation. (G) RP-HPLC and (H) HRMS of full-length H4isoD24. Expected mass = 11278.2 Da; observed mass = 11278.1 Da. Insets depict (B, D) the isotope distribution of the most intense peak or (F, H) the deconvoluted spectrum.

Due to the need to incorporate the nonproteinogenic isoAsp residue at position 24 of histone H4, we decided to adapt a protein semisynthesis approach.^30^ The fortuitous presence of alanine at position 38 suggested a straightforward strategy: Native chemical ligation (NCL) between a C-terminal peptide thioester encompassing residues 1–37 of H4 and a recombinant H4 fragment (residues 38–102) bearing the requisite A38C mutation that could be desulfurized to recover the native sequence.^31−33^ The isoD24- and the D24-containing versions of H4(1–37) were synthesized by Fmoc solid-phase peptide synthesis (SPPS) as C-terminal acyl hydrazides, which could subsequently be converted to the required thioesters.^34^ The peptides were N-terminally acetylated to reflect the presence of this modification *in vivo*,^35^ and purified via reversed-phase chromatography (RP-HPLC). Subsequent analytical RP-HPLC of purified H4D24(1–37) exhibited a single peak (Figure 2A), and high-resolution mass spectrometry (HRMS) confirmed the correct mass (Figure 2B). However, purified H4isoD24(1–37) displayed two major peaks during RP-HPLC (Figure S1A), both exhibiting the expected mass (Figure S1B). This observation suggested the presence of an isomeric peptide in our preparation of H4isoD24(1–37); indeed, coinjection with successfully synthesized H4D24(1–37) confirmed that this peak splitting was due to isomerization of isoD24/D24 (Figure S1C), likely caused by aspartimide formation during the synthesis. To mitigate this issue, we implemented a convergent synthesis to reduce the total number of deprotection cycles, whereby H4isoD24(1–37) would be produced via the NCL of two smaller peptides (Figure 1D). Additionally, we included 0.1 M Oxyma during Fmoc deprotection to suppress aspartimide formation.^36,37^

Thus, peptides H4(1–14) and H4isoD24(A15C, 15–37) were synthesized as C-terminal acyl hydrazides, purified (Figure S2), and ligated upon oxidation of the H4(1–14) peptide to the acyl azide using NaNO_2_ and thioesterification via addition of methyl thioglycolate.^34^ The A15C mutation was reverted by radical desulfurization to generate the desired H4isoD24(1–37) peptide.^32^ This purified peptide exhibited a single peak in analytical RP-HPLC (Figure 2C) with the correct mass confirmed by HRMS (Figure 2D), confirming the suitability of the convergent approach in maintaining the integrity of the isoD24 modification (Figure S3). To ascertain both the successful incorporation of isoAsp into H4isoD24(1–37) and the retention of the canonical residue in H4D24(1–37), we subjected both peptides to methylation by PIMT with subsequent quantification by HRMS. After a 1 h incubation with PIMT and its *S*-adenosyl-l-methionine (AdoMet) cofactor, H4D24(1–37) remained unmethylated (Figure S4A), whereas H4isoD24(1–37) was almost entirely converted to either its methyl-isoAsp analogue or the latter’s succinimidyl byproduct (Figure S4B,C), as expected.

The N-terminal H4 peptides were then elaborated into full-length H4 via NCL with recombinantly produced H4(A38C, 38–102) (Figures 1C,D and S5).^38^ The ligations and subsequent desulfurizations were performed as for the convergent synthesis of H4isoD24(1–37) and yielded 0.36 and 0.42 mg of the desired H4D24 and H4isoD24 proteins, respectively (Figures 2E–H and S6).

### Compaction of Nucleosome Arrays Containing Aged Histone H4

With both full-length H4 variants in hand, we moved on to the incorporation of these substrates into a physiologically relevant chromatinized context. They were refolded with recombinantly produced histones—H2A, H2B, and H3 (Figures S7 and S8)—into octamers (Figure S9) and incorporated into nucleosome arrays with DNA templates containing 12 copies of the strong “601” nucleosome positioning sequence.^39^ The successful preparation of octamers and “12-mer” nucleosome arrays (Figure S10) demonstrates that aged histones, i.e., H4isoD24, are compatible with the assembly of key chromatin components.

**Figure 3:**
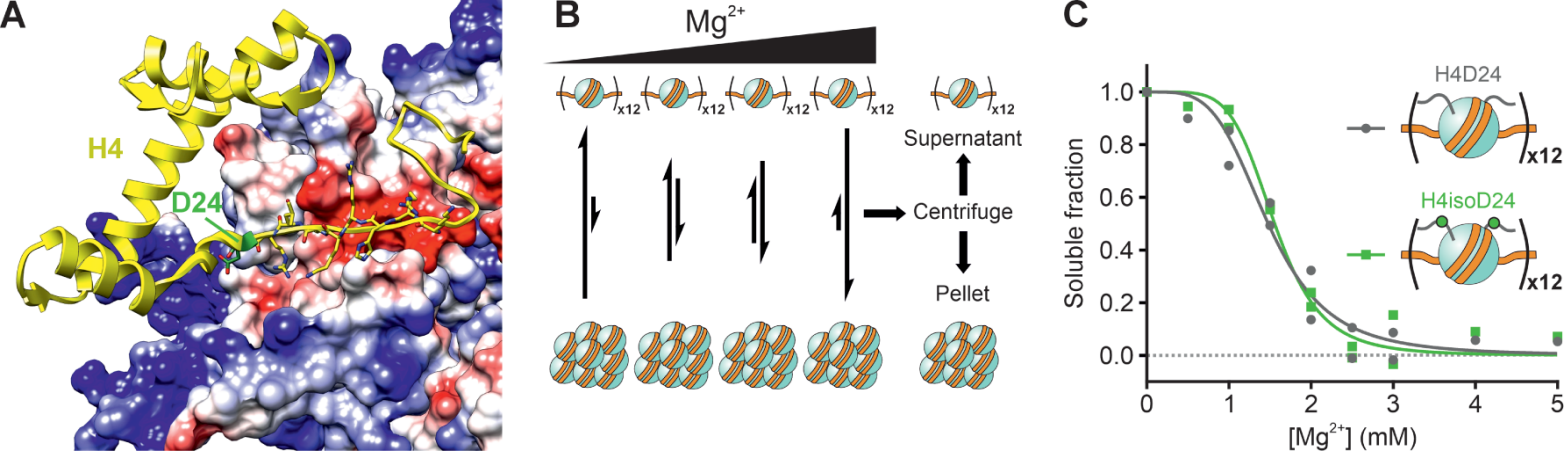
H4isoD24 does not significantly modulate chromatin compaction as measured by Mg^2+^-mediated self-association. (A) The interaction of the acidic patch on H2A/H2B (surface colored according to Coulombic potential: red, negative; white, neutral; blue, positive) with the H4 N-terminal tail of a neighboring nucleosome core particle (H4 in yellow, D24 in green). PDB code: 1KX5.^29^ (B) Scheme of the Mg^2+^-induced self-association assay, in which 12-mers are incubated with varying concentrations of Mg^2+^ and then centrifuged to isolate soluble from oligomerized arrays. (C) Soluble fractions (relative to input) vs [Mg^2+^] (mM) after Mg^2+^-induced self-association, as measured by DNA absorbance at 260 nm for 12-mers containing H4D24 (gray circles) or H4isoD24 (green squares). Points are means of two technical replicates, and measurements at [Mg^2+^] = 0, 1–3 mM contain two replicates from independent 12-mer preparations. Solid gray and green lines are fits of the data to a two-state cooperative model for H4D24- and H4isoD24-containing arrays, respectively. See text for fitting results.

As discussed previously, the disordered H4 N-terminal tail plays a critical role in facilitating the internucleosomal contacts necessary for chromatin compaction, an important regulator of accessibility to the genome.^40^ X-ray crystal structures of the nucleosome core particle place H4D24 at the boundary between the N-terminus of the histone fold and the tail (Figure 3A).^29^ This “gating” position suggests that H4D24 may be a key residue controlling the appropriate positioning of the basic H4 N-terminus and thus its myriad hydrogen bonds with the H2A/H2B acidic patch. Indeed, measurements performed on nucleosome arrays using solid-state NMR spectroscopy assign H4D24 as one of the first relatively rigid residues demarcating the boundary between the N-terminal tail and H4’s more structured regions.^41^ We thus hypothesized that H4isoD24—with its altered structural properties—would modulate the extent of chromatin fiber compaction.

Chromatin folding and oligomerization, i.e., self-association, can be induced *in vitro* by the addition of divalent cations such as Mg^2+^. This *in vitro* self-association has been used extensively to interrogate the structural malleability of chromatin, and the oligomers formed by this induced self-association share several key features with higher-order chromatin condensates found *in vivo*.^42,43^ Thus, we opted to use Mg^2+^-mediated self-association to investigate the possible effects of H4isoD24 on chromatin compaction. H4D24- and H4isoD24-containing 12-mers were incubated with varying concentrations of Mg^2+^ ([Mg^2+^]) and then centrifuged to separate monomers and small oligomers from heavily oligomerized species (Figure 3B). After quantification of the soluble fractions by absorption of DNA at 260 nm, the resulting solubility curves were fit to a two-state cooperative model as a function of [Mg^2+^] (Figure 3C). The Mg^2+^ concentrations at 50% soluble fraction were [Mg^2+^]_50_ = 1.5 ± 0.1 mM (95% CI) and 1.56 ± 0.09 mM (95% CI) for H4D24- and H4isoD24-containing 12-mers, respectively. The Hill coefficients were |*n|* = 4 ± 1 (95% CI) and 5 ± 1 (95% CI) for 12-mers containing H4D24 and H4isoD24, respectively. Thus, the structural responses of the H4isoD24 12-mer and its unmodified counterpart to the addition of Mg^2+^—in terms of both sensitivity and cooperativity—were statistically indistinguishable. At this level of analysis, the presence of H4isoD24 did not significantly alter the internucleosomal contacts which are mediated by the H4 N-terminal tail and are responsible for chromatin compaction.

### Remodeling of MNs Containing Aged Histone H4 by Chd1

**Figure 4:**
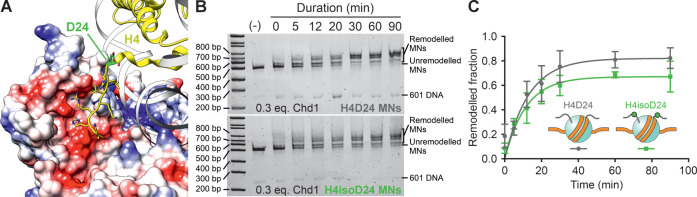
H4isoD24 chromatin is efficiently remodeled by Chd1 *in vitro*. (A) The interaction between the acidic patch on ATPase lobe 2 of *S. cerevisiae* Chd1 (surface colored according to Coulombic potential: red, negative; white, neutral; blue, positive) and the N-terminal tail of nucleosomal H4 (H4 in yellow, D24 in green). PDB code: 5O9G.^49^ (B) An example of the EMSA used to quantify the remodeling efficiency of Chd1 on mononucleosomes (MNs) containing H4D24 (top) or H4isoD24 (bottom). The symmetric MNs produced by Chd1 migrate more slowly under native gel electrophoresis, and remodeled fractions are calculated by normalizing summed intensities of upper bands by total signal with background subtraction (Figure S14). (−) = No Chd1. (C) Remodeled fractions of H4D24- (gray circles) or H4isoD24- (green squares) containing MNs vs time (min). Each time point comprises four to five biological replicates with error bars representing the standard deviations, and solid gray and green lines are fits of the data to a single-exponential association model for MNs with H4D24 or H4isoD24, respectively. See text for fitting results.

Like chromatin compaction, the repositioning of nucleosomes is a key determinant of genomic accessibility. Nucleosome remodelers—the enzymatic factors responsible for this process—thus facilitate DNA transcription, replication, and repair.^44^ The highly conserved chromodomain-helicase-DNA-binding protein 1 (Chd1),^45^ a member of the CHD subfamily of nucleosome remodelers, functions to assemble and regularly space nucleosomes.^46^ In murine contexts, Chd1 has been implicated in both promoter escape of RNA polymerase II^47^ and maintenance of pluripotency in embryonic stem cells.^48^ A cryogenic electron microscopy (cryo-EM) structure reveals that the Chd1 ATPase motor binds to the DNA gyre and is anchored to the N-terminal tail of histone H4, and—reminiscent of the interaction between the H4 N-terminal tail and the H2A/H2B acidic patch discussed earlier—D24 again lies at the interface between the unengaged histone fold and the extensively contacted tail (Figures 4A and S11A).^49^ This arrangement is conserved among archetypal members of the SWI/SNF (Snf2, Figure S11B)^50^ and ISWI (ISW1, Figure S11C)^51^ remodeler subfamilies, suggesting that the interaction observed between Chd1 and the H4 N-terminal tail is a generalizable feature of remodeler-nucleosome engagement.

We thus set out to explore how aged H4 may influence ATP-dependent chromatin reorganization by Chd1 using an electrophoretic mobility shift assay (EMSA). Accordingly, we prepared both H4D24- and H4isoD24-containing mononucleosomes (MNs) using a DNA template with an offset “601” sequence (Figure S12A) and then incubated these asymmetric MNs with Chd1 (Figures S12B,C and S13). Chd1 is known to symmetrize MNs, which in turn leads to slower migration of MNs on native gels,^52^ allowing for quantification of the remodeled MN fraction by normalizing emergent bands of lower mobility to total MN signal (Figures 4B and S14). Chd1 activity was calculated by fitting remodeled fractions as a function of time to single-exponential association models (Figure 4C). For MNs containing H4D24, Chd1 remodeling exhibited an effective rate of *k* = 0.08 ± 0.02 min^–^^1^ (95% CI) and a plateau of *A* = 0.82 ± 0.08 (95% CI). The corresponding parameters for MNs with H4isoD24 were *k* = 0.09 ± 0.02 min^–1^ (95% CI) and *A* = 0.67 ± 0.06 (95% CI). Based on these measurements, H4isoD24-containing nucleosomes are remodeled efficiently by Chd1, albeit with an apparent reduction to the overall extent of remodeling.

### H4K20 Mono- and Dimethylation in H4isoD24-Containing Peptides and Nucleosome Arrays

**Figure 5:**
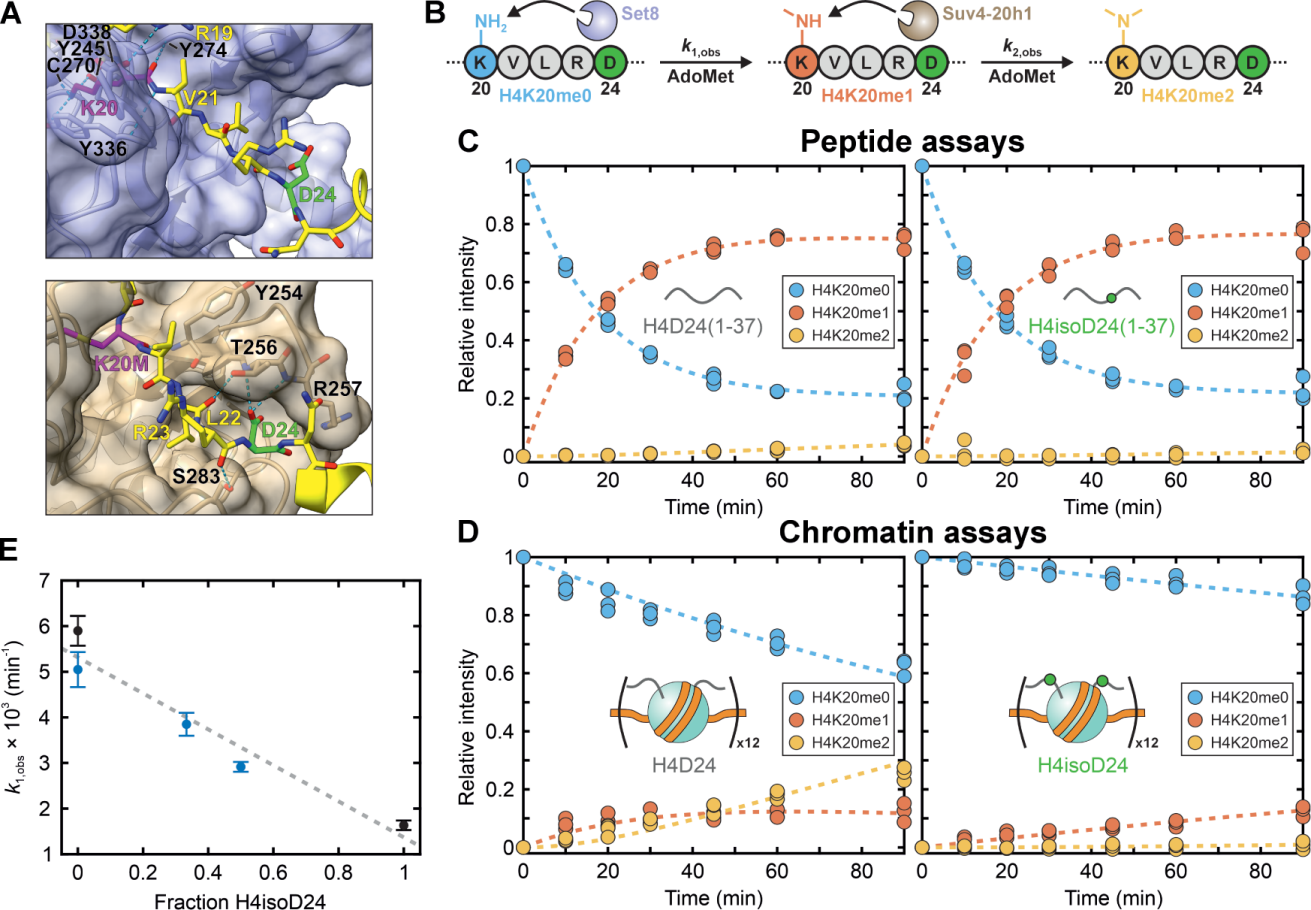
H4isoD24 significantly inhibits the Set8/Suv4-20h1 H4K20 methylation cascade in a chromatin context. (A) Interactions between the H4 N-terminal tail (H4, yellow; H4D24, green; H4K20/H4K20M, magenta) and the *H. sapiens* orthologues of Set8 (purple, top) and Suv4-20h1 (brown, bottom) extracted from cryo-EM structures of enzyme-nucleosome complexes. PDB: 7D1Z(62) and 8JHG,^63^ respectively. Hydrogen bonds denoted with blue, dashed lines, and black labels refer to enzymatic residues. (B) Scheme of the Set8/Suv4-20h1 H4K20 methylation cascade. Utilizing AdoMet, Set8 monomethylates H4K20 (H4K20me0, blue) to H4K20me1 (red), and Suv4-20h1 methylates the latter to H4K20me2 (yellow) with rate constants *k*_1,obs_ and *k*_2,obs_, respectively. H4D24 highlighted in green. (C) Sequential methylation cascade using peptide substrates. Relative intensities as measured by HRMS of H4D24(1–37) (left) and H4isoD24(1–37) (right) peptides exhibiting H4K20me0 (blue circles), H4K20me1 (red circles), and H4K20me2 (yellow circles) after incubation with both Set8 and Suv4-20h1 (All time points from three replicates are shown). Dashed lines represent global fits of the data to a sequential reaction model with a correction factor *p* to account for incomplete methylation (eqs S5–S7). (D) As in (C), but using 12-mer substrates and eqs S8–S10 directly without correction. (E) H4isoD24 exhibits a local effect on Set8 activity. The monomethylation rate *k*_1,obs_ [(min^–1^) × 10^3^] on 12-mers is plotted vs the fractional content of H4isoD24. Black circles represent the rates obtained from the methylation cascades in (D), and blue circles represent those obtained in the absence of Suv4-20h1. All points and error bars are derived from global fits of the data to either a two-step (black) or one-step (blue; eqs S8, S11) reaction model, and the gray, dashed line represents a nonweighted linear regression. The blue data point at fraction H4isoD24 = 0 derives from a singleton measurement, and those at 0.33 and 0.5 from duplicates.

Mg^2+^-mediated self-association and nucleosome remodeling by Chd1, both of which well-tolerated H4isoD24, rely on the H4 N-terminal tail as an anchor point for directing large-scale, reorganizational processes. We thus turned our attention to more local molecular mechanisms that may be perturbed by the presence of aged H4—namely, the installation of nearby PTMs. The four core histones are heavily modified by a plethora of PTMs with varied regulatory roles. Indeed, PTMs have been found on every single residue in the H4 N-terminal tail with a reactive side chain.^53^ In this domain, methylation of H4K20 is associated with key cellular functions. Monomethylation (H4K20me1), dimethylation (H4K20me2), and trimethylation (H4K20me3) are all involved to various degrees in replication timing, cell cycle progression, and DNA repair,^28^ with the latter mark playing an important role in gene silencing/heterochromatin maintenance.^54^ H4K20me1 and H4K20me3 are also implicated in direct facilitation of chromatin decompaction^55^ and compaction,^56^ respectively. The methyltransferase Set8 is the only known enzyme to monomethylate H4K20,^57^ while in higher eukaryotes sequential methylation of H4K20me1 to H4K20me2 is primarily carried out by the paralogues Suv4-20h1 and Suv4-20h2^58^ to such an extent that ∼84% of all H4 in a *HeLa* S3 cell line contains the latter mark.^59^ Activity assays on H4 peptides demonstrate that Asp24 is important for molecular recognition in both Suv4-20h1^60^ and Set8.^61^ These observations can be rationalized based on cryo-EM structures (see Figure S15A,B for Set8^62^ and Figure S15C,D for Suv4-20h1^63^), with Suv4-20h1 making more extensive contacts with the residues C-terminal to H4K20 (Figure 5A). Thus, we hypothesized that both enzymes might be sensitive to H4isoD24.

Our initial experiments used H4(iso)D24(1–37) acyl hydrazide peptides because peptide methylation can be readily monitored by HRMS. We subjected varying concentrations of these peptides to Set8-catalyzed monomethylation (Figures S16 and S17), but determination of the Michaelis–Menten constants was unsuccessful due to the large *K*_m_ values for Set8 monomethylation of peptide substrates^64^ (*K*_m_ = 120 [20, ∞] μM and 28 [19, 44] μM for H4D24(1–37) and H4isoD24(1–37), respectively, with asymmetric 95% CI) (Figure S17G). Instead, substrate preference was approximated using the relative catalytic proficiencies (*k*_cat_/*K*_m_) from linear fits of the velocities at the lowest substrate concentrations, giving *k*_cat_/*K*_m_ = 0.013 ± 0.002 μM^–1^ min^–1^ and 0.016 ± 0.003 μM^–1^ min^–1^ for the unmodified and isoAsp-modified H4 peptides, respectively (Figure S17H). Thus, at the level of peptides, no statistically significant effect on Set8 activity could be discerned. We next attempted a methylation cascade combining H4(1–37) peptide monomethylation by Set8 with subsequent dimethylation by Suv4-20h1 (Figures S18 and S19), assuming a straightforward sequential kinetic model with rate constants *k*_1,obs_ and *k*_2,obs_ (Figure 5B). This assay also did not detect a difference between Asp- and isoAsp-containing H4(1–37), possibly due to Suv4-20h1’s low activity on peptide substrates (Figure 5C).

Given that the observed tolerance of Set8 to the presence of isoAsp may be a consequence of Set8’s preference for nucleosomal substrates^65^—which Suv4-20h1 shares^66^—we decided to investigate the activity of these enzymes on 12-mer nucleosome array substrates. 12-mers containing either H4D24 or H4isoD24 were assembled and validated as before (Figure S20), incubated with Set8, Suv4-20h1, and AdoMet and subjected to HRMS to quantify H4K20me1 and H4K20me2 deposition (Figures S21 and S22) according to the same sequence of irreversible, first-order reactions (Figure 5B). Remarkably, the methylation cascade on H4isoD24-containing 12-mers was significantly inhibited by H4isoD24 as compared to the unmodified control (Figure 5D and Table 1). The rate of Set8 monomethylation of the latter was reduced by >3-fold, and even more strikingly, the rate of Suv4-20h1 dimethylation was decreased >10-fold. As the former observation could result from the decreased production of H4K20me1, we calculated the relative amount of H4K20me2 that would form after 90 min assuming the same *k*_1,obs_ but varying *k*_2,obs_ (Figure S23). Assuming the same experimental precision, *k*_2,obs_ ≈ 0.006 min^–1^ gives a statistically significant upper bound for the dimethylation rate, and so the rate of Suv4-20h1 activity decreases at least 5-fold. Finally, restriction digests confirmed that the 12-mers were intact throughout the enzymatic reactions (Figure S24). This analysis clearly demonstrates that the methylation cascade performed by Set8 and Suv4-20h1, in the context of their physiological, chromatinized substrate, is severely hindered by the presence of H4isoD24. This observation suggests that the bivalent engagement between the methyltransferases and chromatin, involving active site binding to K20 as well as distal recognition of the nucleosome, is perturbed by isoD24. The perturbation is particularly pronounced in Suv4-20h1, whose deep peptide-binding cleft is apparently unable to accommodate the unusual conformation provided by the beta-peptidic linkage in isoD24. Such a pronounced inhibitory effect also suggested the possibility that H4isoD24 may modulate local enzyme activity nonlinearly, with a “critical concentration” of the aged substrate sufficient to significantly alter nearby PTM dynamics. To test this hypothesis, we assembled 12-mer nucleosome arrays containing 2:1 and 1:1 ratios of H4D24- and H4isoD24-containing octamers (Figure S25) and subjected these stochastic constructs to the same reaction conditions, HRMS analyses (Figures S26–S28), and postreaction digests (Figure S29) as for the dimethylation cascade, but now exclusively as a monomethylation reaction in the absence of Suv4-20h1 to simplify analysis (eq S8 and S11). Subsequent estimations of *k*_1,obs_ revealed a negative, linear correlation between the fractional content of H4isoD24 (*F*_isoD_) in the mixed 12-mer species and the overall rate of methylation by Set8 (Figure 5E) of −0.004 ± 0.002 min^–1^/*F*_isoD_, instead supporting a model by which the inhibitory effect of H4isoD24 is exerted locally via molecular deviation from the expected enzymatic substrate.

**Table 1:** Kinetic Parameters for the Set8/Suv4-20h1 Methylation Cascade on H4(iso)D24 Peptides and 12-mers

Substrate	H4 variant	*k*_1,obs_^a^ (min^–1^)	*k*_2,obs_^a^ (min^–1^)	*p*^a^^b^
H4(1–37) peptides	H4D24	0.057 ± 0.002	0.0007 ± 0.0001	0.795 ± 0.007
	H4isoD24	0.058 ± 0.003	0.0003 ± 0.0003	0.78 ± 0.01
12-mers	H4D24	0.0059 ± 0.0003	0.033 ± 0.005	—
	H4isoD24	0.0016 ± 0.0001	0.002 ± 0.002	—

a Ranges represent 95% CI, obtained from fits to the data shown in Figure 5C,D.

b Correction factor reflecting incomplete methylation. See eqs S5–S7.

## Discussion

As long-lived proteins, histones undergo many nonenzymatic, spontaneous modifications. For example, glycation—a key indicator of diabetes—is a stable adduct formed by condensation of monosaccharides or glycolytic byproducts with primary amino/guanidino moieties. This PTM is known to occur on histones, with observed *in vitro* and *in cellulo* consequences on the maintenance of chromatin structure.^67^ Exogenous DNA damaging reagents such as ionizing radiation can induce abasic DNA lesions that can subsequently react with and modify key regulatory histone lysines such as H4K12, H4K16, and H4K20.^68^ Here we focus on the spontaneous rearrangement of Asn and Asp residues to their β-linked isomer, a process that occurs within the lifetimes of histones and is unique in that it does not require an exogenous chemical agent and instead arises from the inherent reactivity of the canonical amino acids.

Traditionally considered as undesirable protein aging, growing evidence indicates that the Asp isomerization/Asn deamidation reaction pathway is involved in a variety of biological processes and is possibly harnessed as a molecular timer that changes the binding affinities of interaction partners. The PTMs of this pathway have been implicated in the regulation of the tumor suppressor p53,^69^ host recognition by human noroviruses,^70^ modulation of the binding interactions of fibronectin in the extracellular matrix,^71^ and deactivation of the antiapoptotic factor Bcl-x_L_.^72^ Additionally, both the succinimidyl and isoAsp products of Asn deamidation were found to play stable structural roles in proteins, suggesting that evolutionary control of Asp isomerization/Asn deamidation is operative via selection of the surrounding primary sequence.^73,74^

In this study, we investigated the molecular consequences of isoAsp formation at position 24 of histone H4.^20^ This necessitated the production of well-defined substrates containing this PTM. Thus, we harnessed protein semisynthesis to generate site-specifically modified, full-length H4isoD24 and then incorporated this construct into precise mono- and polynucleosomal templates. Subsequent experiments revealed that chromatin compaction, measured by Mg^2+^-mediated self-association, and remodeling by Chd1 tolerated H4isoD24 despite this modification’s disruptive changes to the backbone’s spacing and direction and its unusual repositioning of a negative charge. In contrast, the methylation cascade carried out on H4K20 by Set8 and Suv4-20h1 was strongly inhibited by the presence of aged H4, with both enzymes individually exhibiting reductions in activity of >70%. This “crosstalk” between aging in the form of H4isoD24 and H4K20me is reminiscent of the long-established biochemical interactions between differing histone PTMs.^75^

At first glance, the mechanism-dependent sensitivities toward H4isoD24 observed in these different processes may be a consequence of both the degree of isoAsp involvement in the underlying interactions and the characteristics of these interactions themselves. For chromatin compaction mediated by interactions between the H4 N-terminal tail and the H2A/H2B acidic patch, H4isoD24 is not actively engaged with this interface and instead lies at its C-terminal boundary. Additionally, the internucleosomal interaction this PTM may be conformationally gating is highly dynamic; Solid-state NMR measurements reveal that the H4 N-terminal tail remains dynamic even at the high > ∼200 mg/mL concentrations found in the cell.^41^ The relationship of H4isoD24 to the Chd1-nucleosome complex, which also utilizes a similar interaction with the H2A/H2B acidic patch, can likely be described in comparable terms. In contrast, Set8 and Suv4-20h1 interactions with the H4 N-terminal tail are defined by a highly specific recognition of the latter’s primary sequence—a model that aligns with the observed negative, linear correlation between the former enzyme’s activity and the presence of H4isoD24. The steric and electrostatic perturbations induced by isoAsp (and in the case of Suv4-20h1, direct disruption of hydrogen bonds to the canonical Asp) would thus be expected to have a disproportionate influence on these enzymes’ activities.

It is worth emphasizing, however, that the observed macroscopic robustness of chromatin architecture—i.e., compaction and nucleosome remodeling—toward aging of histone H4 is remarkable considering that isoAsp was present at stoichiometric levels in the corresponding assays. Such processes would be particularly sensitive because any perturbation can be amplified beyond the site of damage: chromatin compaction is a highly cooperative, internucleosomal process,^42^ and Chd1’s high dwell times on MNs, as well as the flexible linker present between its ATPase and DNA-binding domains, suggest that it may act processively on neighboring nucleosomes.^76^ Thus the resilience to a magnified prevalence of H4isoD24 suggests that—given the likely substoichiometric levels of this mark *in vivo*—there was limited evolutionary pressure to prevent the aging-related formation of isoAsp at this site. On the other hand, both Set8 and Suv4-20h1 lack the domain architecture seen in other histone methyltransferases that can be allosterically activated by their own deposited PTM.^77^ The inhibition of H4K20 methylation is therefore—initially, at least—a local effect which can potentially be bypassed by unaffected H4 N-terminal tails within the same or adjacent MNs. It may be the case that maintenance of chromatin integrity is promoted by limiting the effects of isoAsp formation to those processes that are not inherently amplified, thus minimizing disruption prior to repair or degradation. Whether this correlation is generally relevant and can be extrapolated to other instances of isoAsp formation, however, remains to be seen.

## Conclusion

Collectively, our results demonstrate that chromatin exhibits both a considerable robustness and vulnerability toward the molecular aging of histone H4, with implications for the structural and epigenetic maintenance of the genome. However, these findings also make clear that the biochemical and physical effects of isoAsp are difficult to generalize, and their interrogation requires the use of chemically well-defined substrates in precise *in vitro* experiments. We anticipate that our bottom-up chemical biology approach will provide a template for future investigations of this unique PTM in chromatin and beyond.

## Supporting Information Available

Experimental details, materials and methods. Characterization of synthetic peptides (RP-HPLC and MS), histones (RP-HPLC, MS and gel), octamers (SEC and gel), nucleosome arrays (native gels and digests). Raw data of remodeling assays (gels) and methyltransferase assays (MS). Limit of detection analysis (PDF)
