# Supplementary material for: Semisynthesis of Isomerized Histone H4 Reveals Robustness and Vulnerability of Chromatin toward Molecular Aging
Source: J Am Chem Soc. 2025 Feb 3;147(6):4952–61. doi: 10.1021/jacs.4c14136 (PMC11826994; doi:10.1021/jacs.4c14136)
Supplement: Supplementary file 1 — ja4c14136_si_001.pdf [file ja4c14136_si_001.pdf]

Supporting Information for:

Semisynthesis of Isomerized Histone H4 Reveals Robustness and Vulnerability of Chromatin toward Molecular Aging

Tianze Zhang[1§†], Luis F. Guerra[1†], Yana Berlina[2], Jon R. Wilson[3], Beat Fierz[2], Manuel M. Müller[1]*

1. Department of Chemistry, King's College London, Britannia House, 7 Trinity Street, SE1 1DB London, UK

2. École Polytechnique Fédérale de Lausanne (EPFL), ISIC, CH-1015 Lausanne, Switzerland

3. The Francis Crick Institute, 1 Midland Road, NW1 1AT London, UK

§ Present address: The Francis Crick Institute, 1 Midland Road, NW1 1AT London, UK

† These authors contributed equally to this work.

*Correspondence to M.M.M. (manuel.muller@kcl.ac.uk)

Contents

Experimental Procedures 5
General materials and methods 5
General procedure for reversed-phase high-performance liquid chromatography (RP-HPLC) 6
Mass spectrometry....6
Protein sequences 7
DNA sequences for mononucleosome (MN) and 12-mer nucleosome array production....8
Resin functionalization with hydrazine....10
Solid-phase synthesis and purification of all peptides 10
Peptide Ac-H4D24(1–37)-$NHNH_2$....11
Peptide Ac-H4isoD24(1–37)-$NHNH_2$ (linear synthesis)....11
Peptide Ac-H4(1–14)-$NHNH_2$ 12
Peptide H4isoD24(A15C, 15–37)-$NHNH_2$....12
Peptide Ac-H4isoD24(1–37)$NHNH_2$ (convergent synthesis) 13
Expression and purification of recombinant histone proteins....14
Expression and purification of truncated H4 38−102 (A38C)....14
Expression and purification of Chd1....15
Expression and purification of Suv4-20h1 16
Native chemical ligation and desulfurisation of H4(iso)D24(1–102) 16
PIMT methylation of H4D24(1–37) and H4isoD24(1–37) peptides 16
Scheme S1. Reaction diagram for PIMT-mediated methylation of isoAsp containing peptides....18
Octamer assembly....18
MN assembly....18
Assembly of 12-mers 19
Quality checking of 12-mers....19
Mg^{2+}-mediated self-association of H4(iso)D24 12-mers 19
Chd1 remodelling of H4(iso)D24 MNs....20
Set8 monomethylation on H4(iso)D24(1-37) peptides....20
Set8/Suv4-20h1 H4K20 dimethylation cascade on H4(iso)D24(1–37) peptides 20
Set8/Suv4-20h1 H4K20 dimethylation cascade on H4(iso)D24 12-mers....22

Set8 monomethylation of H4(iso)D24 12-mers....23
Supplementary Figures....25
Figure S1: Analysis of linearly synthesised H4isoD24(1-37) peptide....25
Figure S2: Analysis of H4isoD24(1-37) peptide building blocks....26
Figure S3: Comparison of H4D24(1-37) and H4isoD24(1-37) peptides....27
Figure S4: PIMT assays on H4 peptides....28
Figure S5: Analysis of H4(A38C, 38–102)....29
Figure S6. Synthesis of full-length H4 via native chemical ligation....30
Figure S7. HPLC analysis of purified recombiant histones....30
Figure S8: HRMS analysis of purified recombinantly-produced histones....32
Figure S9: Purification and analysis of histone octamers....32
Figure S10: Analyses of H4(iso)D24-containing 12-mers....33
Figure S11. Interactions between the H4 N-terminal tail and selected nucleosome remodellers....34
Figure S12. MN assembly and Chd1 purification....35
Figure S13. Native PAGE of remodelling assays....36
Figure S14: Example analysis of Chd1 remodelling assays on MNs....37
Figure S15. Set8 and Suv4-20h1 interactions with the nucleosome and/or H4....38
Figure S16. Set8 monomethylation of H4(iso)D24(1–37) peptides, example HRMS time course....39
Figure S17. Set8 monomethylation of H4(iso)D24(1–37) peptides, analyses....40
Figure S18: TICs of H4 peptide methyltransferase assays....41
Figure S19: Mass spectra of H4 peptide methyltransferase assays....42
Figure S20: Analysis of 'designer' nucleosome arrays used for dimethylation assays....43
Figure S21: TICs of nucleosome array dimethylation assays....44
Figure S22. Deconvoluted mass spectra of nucleosome array dimethylation assays....45
Figure S23. Limit-of-detection analysis for the quantification of $k_{2,obs}$....46
Figure S24. Analysis of nucleosome array integrity after dimethylation assays....47
Figure S25. Analysis of 'designer' nucleosome arrays used for monomethylation assays....48
Figure S26. TICs of nucleosome array monomethylation assays....49
Figure S27. Deconvoluted mass spectra of nucleosome array monomethylation assays....50
Figure S28. Kinetics of nucleosome array monomethylation assays....51
Figure S29. Analysis of nucleosome array integrity after monomethylation assays....51

Supplementary References 52

Experimental Procedures

General materials and methods

Peptide resins, Fmoc-L-amino acids, the monoprotected isoAsp building block Fmoc-Asp-OtBu, and Oxyma were purchased from Novabiochem. Diisopropylethylamine (DIEA), piperidine, reduced L-glutathione, and tris(2-carboxyethyl)phosphine hydrochloride (TCEP) were purchased from Merck Sigma-Aldrich. *N*-methyl-2-pyrrolidone (NMP), diisopropylcarbodiimide (DIC), triisopropylsilane (TIS), and dichloromethane (DCM) were purchased from Merck. Peptide synthesis grade dimethylformamide (DMF) was purchased from Cambridge Reagents Ltd. Methyl thioglycolate was purchased from Sigma-Aldrich. VA-044 was purchased from FUJIFILM Wako Pure Chemical Corporation. Peptide grade trifluoroacetic acid (TFA) was purchased from FluoroChem. Dithiothreitol (DTT) was purchased from both AnaSpec and Fluorochem. *S*-adenosyl-L-methionine (AdoMet) was purchased from Sigma-Aldrich and stored at 5 mM in 20 mM HCl. Set8 (195–352; UniProt: Q9NQR1-2; GenBank: NM_020382) was purchased as a GST-tag fusion from either Sigma-Aldrich (SRP0150-50UG; Used for monomethylation of H4 peptides) or Cambridge Bioscience (BPS-51008; Used for Set8/Suv4-20h1 methylation cascades on H4 peptides and 12-mers) and used without cleavage of the affinity tag.

Reagents and solvents were used without further purification, and all H_2O was deionised to resistivities of 15 or 18.2 mΩ·cm. DNA and native protein concentrations were determined using a NanoDrop ND-8000, 8-Sample spectrophotometer (Labtech) and absorbances at 260 nm (A_{260}) or 280 nm (A_{280}), respectively, with sequence-derived extinction coefficients. Sequencing of plasmids was obtained through Genewiz. All *E. coli* competent cells were made in-house from commercial strains: TOP10 for cloning; BL21(DE3) and Rosetta (DE3) for expression.

Peptide concentrations were quantified using peak areas obtained via analytical RP-HPLC with 214 nm detection.[1]

The enzymes Ulp1 (used for cleavage of SUMO solubility tags) and PIMT (used for methylation of L-isoAsp) were N-terminally H_6-tagged, and both were produced and purified by Ni-NTA chromatography under standard conditions. Note that the affinity tags were not cleaved before use.

The 12x“601” DNA template was generously provided by the Muir lab (Princeton University), and MMTV DNA was purchased as a synthetic gene (Genewiz) and produced by large-scale PCR.

All molecular graphics were prepared with UCSF ChimeraX.[2]

All custom Python and MATLAB scripts used for the analyses in Figures 5, S4, and S23 can be found at https://github.com/MuellerLab-KCL/H4isoD24.

All raw data can be found in the King's Open Research Data System (KORDS) with DOI: 10.18742/27150027.

General procedure for reversed-phase high-performance liquid chromatography (RP-HPLC)

Analytical and semi-preparative RP-HPLC was performed on an Agilent 1260 Infinity II instrument equipped with a DAD UV-VIS detector (G4212B). For analytical work, a ZORBAX 300SB-C18, 4.6x150 mm, 3.5 µm column was used at a constant flow of 1 mL/min. In semi-preparative mode, a ZORBAX 300SB-C18, 9.4x250 mm, 5 µm column was used at a flow rate of 5 mL/min. Typical gradients using mixtures of H_2O containing 0.1% TFA (solvent A) and acetonitrile containing 0.1% TFA (solvent B) are described below.

- Gradient LC-A: Linear gradient from 5–35% B over 40 min; Room temperature (RT)
- Gradient LC-B: Linear gradient from 0–50% B over 29 min; 30 °C
- Gradient LC-C: Linear gradient from 0–30% B over 40 min; RT
- Gradient LC-D: Linear gradient from 15–40% B over 40 min; RT
- Gradient LC-E: Linear gradient from 10–50% B over 40 min; RT
- Gradient LC-F: Linear gradient from 5–50% B over 40 min; RT
- Gradient LC-G: Linear gradient from 30–70% B over 40 min; RT
- Gradient LC-H: Linear gradient from 0–70% B over 29 min; 25 °C
- Gradient LC-I: Linear gradient from 20–70% B over 40 min; RT

HPLC purification of the peptides was performed on an Agilent 1260 Preparative HPLC system equipped with a MWD UV-VIS detector (G7165A) using a reversed phase ZORBAX 300SB-C18, 21.2x150 mm, 7 µm column running at 20 mL/min.

Mass spectrometry

High-resolution mass spectra (HRMS) were recorded on a Xevo G2-XS QTof (Waters) after separation by reversed-phase liquid chromatography on an Acquity UPLC (Waters). Peptides were analysed on an Acquity UPLC BEH C18, 130 Å, 1.7 µm, 2.1x50 mm column (Waters), and proteins were analysed on an Acquity UPLC Protein BEH C4, 300 Å, 1.7 µm, 2.1x50 mm column (Waters). All HRMS analyses were performed with at a flow rate of 0.4 mL/min unless stated otherwise. Typical gradients using mixtures of H_2O containing 0.1% formic acid (solvent C) and acetonitrile containing 0.1% formic acid (solvent D) are described below:

- Gradient MS-A: Linear gradient from 5–75% D over 4.5 min; 60 °C
- Gradient MS-B: Linear gradient from 5–75% D over 3.5 min; 40 °C
- Gradient MS-C: Linear gradient from 0–75% D over 3.5 min; 60 °C
- Gradient MS-D: Linear gradient from 5–75% D over 2 min; 60 °C
- Gradient MS-E: Linear gradient from 20–40% D over 8 min; 40 °C

For HRMS validation of purified peptides, deconvolutions of the obtained mass spectra were performed with the UNIFI software platform (Waters) using the MaxEnt3 algorithm with the following parameters:

- Input m/z range: 50–3000 Da/e
- Output range: 400–4000 Da/e

- Maximum charge: 4
- Width model: Tof, with resolution = 30000.0
- Molecule type: Protein
- Charge carrier: Hydrogen
- Iterations: 20
- Ensemble members: 1

For HRMS validation of purified proteins, deconvolutions of the obtained mass spectra were again performed with UNIFI, but now using the MaxEnt1 algorithm with the following parameters:

- Input range: 500–3000 Da/e
- Output range: **Dependent on expected mass of product**
- Peak width model: Tof, with resolution = 30000.0
- Enable noise autoscaling: Yes
- Iterate to convergence: Yes
- Charge carrier: Hydrogen
- Output resolution: 1.0000 Da
- Minimum left and right intensities: 30%

Protein sequences

- H2A (UniProt: Q6FI13; N-terminal Met is cleaved during expression)

MSGRGKQGGKARAKAKSRSSRAGLQFPVGRVHRLLRKGNYAERVGAGAPVYMAAVLEYLTAEILELA
GNAARDNKKTRIIPRHLQLAIRNDEELNKLLGKVTIAQGGVLPNIQAVLLPKKTESHHKAKGK

- H2B (UniProt: O60814; N-terminal Met is cleaved during expression)

MPEPAKSAPAPKKGSKKAVTKAQKKDGKKRKRSRKESYSVYVYKVLKQVHPDTGISSKAMGIMNSFV
NDIFERIAGEASRLAHYNKRSTITSREIQTAVRLLLPGELAKHAVSEGTKAVTKYTSAK

- H3 (UniProt: P68431 – C->A mutations highlighted in red; N-terminal Met is cleaved during expression)

MARTKQTARKSTGGKAPRKQLATKAARKSAPATGGVKKPHRYRPGTVALREIRRYQKSTELLIRKLPF
QRLVREIAQDFKTDLRFQSSAVMALQEAAEAYLVGLFEDTNLAAIHAKRVTIMPKDIQLARRIRGERA

- H_6-SUMO-H4(A38C, 38–102) (UniProt, SUMO: Q12306 – bold; UniProt, H4: P62805 - underlined)

MGSSHHHHHHSSG**MSDSEVNQEAKPEVKPEVKPETHINLKVSDGSSEIFFKIKKTTPLRRLMEAFAK**
RQGKEMDSLRFLYDGIRIQADQTPEDLDMEDNDIIEAHREQIGGCRRGGVKRISGLIYEETRGVLKVFL
ENVIRDAVTYTEHAKRKTVTAMDVVYALKRQGRTLYGFGG

- Suv4-20h1(61-327) (UniProt: Q3U8K7 – Underlined; numbering as in ref. [3])

GPLGSHMSSGMSAKELCENDDLATSLVLDPYLGFQTHKMNTSAFPSRSSRHISKADSFSHNNPVRFR
PIKGRQEELKEVIERFKKDEHLEKAFKCLTSGEWARHYFLNKNKMQEKLFKEHVFIYLRMFATDSGFEIL
PCNRYSSEQNGAKIVATKEWKRNDKIELLVGCIAELSEIEENMLLRHGENDFSVMYSTRKNCAQLWLG
PAAFINHDCRPNCKFVSTGRDTACVKALRDIEPGEEISCYYGDGFFGENNEFCECYTCERRGTGAFKS
R

- Chd1 (UniProt: P32657)

MAAKDISTEVLQNPELYGLRRSHRAAAHQQNYFNDSDDEDDEDNIKQSRRKRMTTIEDDEDEFEDEE
GEEDSGEDEDEEDFEEDDDYYGSPIKQNRSKPKSRTKSKSKSKPKSQSEKQSTVKIPTRFSNRQNKTV
NYNIDYSDDDLLESEDDYGSEEALSEENVHEASANPQPEDFHGIDIVINHRLKTSLEEGKVLEKTVPDLN
NCKENYEFLIKWTDESHLHNTWETYESIGQVRGLKRLDNYCKQFIIEDQQVRLDPYVTAEDIEIMDMER
ERRLDEFEEFHVPERIIDSQRASLEDGTSQLQYLVKWRRLNYDEATWENATDIVKLAPEQVKHFQNRE
NSKILPQYSSNYTSQRPRFEKLSVQPPFIKGGELRDFQLTGINWMAFLWSKGDNGILADEMGLGKTVQ
TVAFISWLIFARRQNGPHIIVVPLSTMPAWLDTFEKWAPDLNCICYMGNQKSRDTIREYEFYTNPRAKG
KKTMKFNVLLTTYEYILKDRAELGSIKWQFMAVDEAHRLKNAESSLYESLNSFKVANRMLITGTPLQNNI
KELAALVNFLMPGRFTIDQEIDFENQDEEQEEYIHDLHRRIQPFILRRLKKDVEKSLPSKTERILRVELSD
VQTEYYKNILTKNYSALTAGAKGGHFSLLNIMNELKKASNHPYLFDNAEERVLQKFGDGKMTRENVLR
GLIMSSGKMVLLDQLLTRLKKDGHRVLIFSQMVRMLDILGDYLSIKGINFQRLDGTVPSAQRRISIDHFNS
PDSNDFVFLLSTRAGGLGINLMTADTVVIFDSDWNPQADLQAMARAHRIGQKNHVMVYRLVSKDTVEE
EVLERARKKMILEYAIISLGVTDGNKYTKKNEPNAGELSAILKFGAGNMFTATDNQKKLEDLNLDDVLNH
AEDHVTTPDLGESHLGGEEFLKQFEVTDYKADIDWDDIIPEEELKKLQDEEQKRKDEEYVKEQLEMMN
RRDNALKKIKNSVNGDGTAANSDSDDDSTSRSSRRRARANDMDSIGESEVRALYKAILKFGNLKEILDE
LIADGTLPVKSFEKYGETYDEMMEAAKDCVHEEEKNRKEILEKLEKHATAYRAKLKSGEIKAENQPKDN
PLTRLSLKKREKKAVLFNFKGVKSLNAESLLSRVEDLKYLKNLINSNYKDDPLKFSLGNNTPKPVQNWS
SNWTKEEDEKLLIGVFKYGYGSWTQIRDDPFLGITDKIFLNEVHNPVAKKSASSSDTTPTPSKKGKGITG
SSKKVPGAIHLGRRVDYLLSFLRGGLNTKSPSADIGSKKLPTGPSKKRQRKPANHSKSMTPEITSSEPA
NGPPSKRMKALPKGPAALINNTRLSPNSPTPPLKSKVSRDNGTRQSSNPSSGSAHEKEYDSMDEEDC
RHTMSAIRTSLKRLRRGGKSLDRKEWAKILKTELTTIGNHIESQKGSSRKASPEKYRKHLWSYSANFWP
ADVKSTKLMAMYDKITESQKK

- H_6-PIMT (UniProt: P22061-2 - underlined; N->K mutation highlighted in red)

MHHHHHHSSGLVPRGSGMKETAAAKFERQHMDSPDLGTDDDDKAMAWKSGGASHSELIHNLRKKGII
KTDKVFEVMLATDRSHYAKCNPYMDSPQSIGFQATISAPHMHAYALELLFDQLHEGAKALDVGSGSGIL
TACFARMVGCTGKVIGIDHIKELVDDSVNNVRKDDPTLLSSGRVQLVVGDGRMGYAEEAPYDAIHVGA
AAPVVPQALIDQLKPGGRLILPVGPAGGNQMLEQYDKLQDGSIKMKPLMGVIYVPLTDKEKQWSRDEL

DNA sequences for mononucleosome (MN) and 12-mer nucleosome array production

- Offset 1x“601” template for MN assembly (147 bp of the nucleosome core particle are underlined[4])

GGATAGTGTTCCGAGCTCCCACTCTAGAGGATCCATCCAGTGAATTCGCACACTGTGCCAAGTAC
TTACGCGGCTACACTGGAGAATCCCGGTGCCGAGGCCGCTCAATTGGTCGTAGACAGCTCTAGCA

CCGCTTAAACGCACGTACGCGCTGTCCCCCGCGTTTTAACCGCCAAGGGGATTACTCCCTAGTCT
CCAGGCACGTGTCAGATACTGCAGAGATCT

- 12x“601” template for 12-mer assembly (ScaI restriction sites are in bold; 147 bp of the nucleosome core particle are underlined[4]).

ATCACGCGGCCGCCCTGGAGAATCCCGGTGCCGAGGCCGCTCAATTGGTCGTAGACAGCTCTAG
CACCGCTTAAACGCACGTACGCGCTGTCCCCCGCGTTTTAACCGCCAAGGGGATTACTCCCTAGT
CTCCAGGCACGTGTCAGATATATACATCCTGTGCATGTAAGATCC**AGTACT**ACGCGGCCGCCCTG
GAGAATCCCGGTGCCGAGGCCGCTCAATTGGTCGTAGACAGCTCTAGCACCGCTTAAACGCACGT
ACGCGCTGTCCCCCGCGTTTTAACCGCCAAGGGGATTACTCCCTAGTCTCCAGGCACGTGTCAGA
TATATACATCCTGTGCATGTAAGATCC**AGTACT**ACGCGGCCGCCCTGGAGAATCCCGGTGCCGAG
GCCGCTCAATTGGTCGTAGACAGCTCTAGCACCGCTTAAACGCACGTACGCGCTGTCCCCCGCGT
TTTAACCGCCAAGGGGATTACTCCCTAGTCTCCAGGCACGTGTCAGATATATACATCCTGTGCATG
TAAGATCC**AGTACT**ACGCGGCCGCCCTGGAGAATCCCGGTGCCGAGGCCGCTCAATTGGTCGTA
GACAGCTCTAGCACCGCTTAAACGCACGTACGCGCTGTCCCCCGCGTTTTAACCGCCAAGGGGAT
TACTCCCTAGTCTCCAGGCACGTGTCAGATATATACATCCTGTGCATGTAAGATCC**AGTACT**ACGC
GGCCGCCCTGGAGAATCCCGGTGCCGAGGCCGCTCAATTGGTCGTAGACAGCTCTAGCACCGCT
TAAACGCACGTACGCGCTGTCCCCCGCGTTTTAACCGCCAAGGGGATTACTCCCTAGTCTCCAGG
CACGTGTCAGATATATACATCCTGTGCATGTAAGATCC**AGTACT**ACGCGGCCGCCCTGGAGAATC
CCGGTGCCGAGGCCGCTCAATTGGTCGTAGACAGCTCTAGCACCGCTTAAACGCACGTACGCGC
TGTCCCCCGCGTTTTAACCGCCAAGGGGATTACTCCCTAGTCTCCAGGCACGTGTCAGATATATAC
ATCCTGTGCATGTAAGATCC**AGTACT**ACGCGGCCGCCCTGGAGAATCCCGGTGCCGAGGCCGCT
CAATTGGTCGTAGACAGCTCTAGCACCGCTTAAACGCACGTACGCGCTGTCCCCCGCGTTTTAAC
CGCCAAGGGGATTACTCCCTAGTCTCCAGGCACGTGTCAGATATATACATCCTGTGCATGTAAGAT
CC**AGTACT**ACGCGGCCGCCCTGGAGAATCCCGGTGCCGAGGCCGCTCAATTGGTCGTAGACAGC
TCTAGCACCGCTTAAACGCACGTACGCGCTGTCCCCCGCGTTTTAACCGCCAAGGGGATTACTCC
CTAGTCTCCAGGCACGTGTCAGATATATACATCCTGTGCATGTAAGATCC**AGTACT**ACGCGGCCG
CCCTGGAGAATCCCGGTGCCGAGGCCGCTCAATTGGTCGTAGACAGCTCTAGCACCGCTTAAAC
GCACGTACGCGCTGTCCCCCGCGTTTTAACCGCCAAGGGGATTACTCCCTAGTCTCCAGGCACGT
GTCAGATATATACATCCTGTGCATGTAAGATCC**AGTACT**ACGCGGCCGCCCTGGAGAATCCCGGT
GCCGAGGCCGCTCAATTGGTCGTAGACAGCTCTAGCACCGCTTAAACGCACGTACGCGCTGTCC
CCCGCGTTTTAACCGCCAAGGGGATTACTCCCTAGTCTCCAGGCACGTGTCAGATATATACATCCT
GTGCATGTAAGATCC**AGTACT**ACGCGGCCGCCCTGGAGAATCCCGGTGCCGAGGCCGCTCAATT
GGTCGTAGACAGCTCTAGCACCGCTTAAACGCACGTACGCGCTGTCCCCCGCGTTTTAACCGCCA
AGGGGATTACTCCCTAGTCTCCAGGCACGTGTCAGATATATACATCCTGTGCATGTAAGATCC**AGT
ACT**ACGCGGCCGCCCTGGAGAATCCCGGTGCCGAGGCCGCTCAATTGGTCGTAGACAGCTCTAG
CACCGCTTAAACGCACGTACGCGCTGTCCCCCGCGTTTTAACCGCCAAGGGGATTACTCCCTAGT
CTCCAGGCACGTGTCAGATATATACATCCTGTGCATGTAAGATCTGAT

- MMTV DNA

ACTTGCAACAGTCCTAACATTCACCTCTTGTGTGTTTGTGTCTGTTCGCCATCCCGTCTCCGCTCG TCACTTATCCTTCACTTTCCAGAGGGTCCCCCCGCAGACCCCGGCGACCCTCAGGTCGGCCGACT GCGGCACAGTTTTTTG

Resin functionalization with hydrazine

Typically, 0.53 g of 2-Cl-Trt-resin (0.85 mmol, 1 eq., substitution: 1.60 mmol/g) were swollen in 3 mL DMF for 15 min at 4 °C on ice. Subsequently, triethylamine (2.57 mmol, 360 µL, 3 eq.) was added. Then a solution of hydrazine monohydrate (1.7 mmol, 85 µL, 2 eq.) in DMF was added dropwise. The reaction mixture was stirred for 1 h at RT. Then 2 mL methanol (MeOH) were added, and the resin was further stirred 15 min at RT to quench the excess of reactive chloride sites on the resin. Then the mixture was transferred to a reaction vessel and washed with 2x8 mL DMF; 2x5 mL H_2O; 2x8 mL DMF. As the hydrazine-functionalized resin is not stable, the C-terminal residue (leucine for the H4(iso)D24(1–37) and H4isoD24(A15C, 15–37) fragments; glycine for the H4(1–14) fragment) was manually coupled by standard Fmoc chemistry. In a separate conical tube, Fmoc-Leu/Gly (4 eq. = 3.4 mmol), Oxyma (4 eq. = 3.4 mmol), DIC (4 eq. = 3.4 mmol) were mixed and dissolved with a minimum amount of DMF (3.5 mL). The mixture was added to the resin and reacted while bubbling N_2 for 45 min at RT. The excess reagents were removed by filtration and another 4 eq. of fresh reagents were added and reacted for 45 min. The resin was washed again by bubbling and flow wash using DMF, and a final wash with DCM and then sufficiently dried under vacuum. Resin loading was determined by weighing out a certain amount (around 5 mg) of resin and treating with 1 mL 20% piperidine in DMF for 10 min at RT, followed by measuring the UV absorbance at 290 nm for the quantification of released Fmoc group. The loadings of Fmoc-Leu-NHNH-Cl-Trt and Fmoc-Gly-NHNH-Cl-Trt were approximately 0.57 mmol/g (36 % yield) and 0.71 mmol/g (45 % yield), respectively. Dried resins were stored in a desiccator for future use.

Solid-phase synthesis and purification of all peptides

Peptides were synthesized via Fmoc-based synthesis with DIC/Oxyma on a Biotage Initiator+ Alstra synthesizer. Typically, the resin was swelled in DCM (60 min, RT) followed by DMF (20 min, 70 °C). Fmoc-deprotection was done in two rounds with 20% piperidine in DMF (3 min, RT followed by 10 min, RT). To minimise aspartimide formation at (iso)Asp24, the deprotection reagent for H4isoD24(A15C, 15–37) was 20% piperidine in DMF with the addition of 0.1 M Oxyma. Amino acids were double coupled using 4 eq. amino acid (0.5 M stocks in DMF) and as coupling reagents correspondingly 4 eq. Oxyma (0.5 M stock in DMF) and 4 eq. DIC (0.5 M stock in DMF). Equivalents were increased if required to ensure sufficient mixing during the coupling. Each coupling was for 60 min at RT. Because arginine truncation is common in the synthesis of H4 N-terminal peptides, capping with acetic anhydride was performed after coupling of each arginine to simplify purification. Both this latter capping and N-terminal acetylation were performed with 50 eq. acetic anhydride in DMF and 50 eq. DIEA in NMP for 10 min at RT with subsequent washing with DCM. After complete synthesis, resins were washed with DCM, dried, and stored under vacuum.

After synthesis, peptides were cleaved from the resin by treatment with cleavage cocktail (95% TFA, 2.5% H_2O, 2.5% triisopropylsilane) and precipitated with diethyl ether, dissolved in 50% B, lyophilised, and

subsequently purified via semi-preparative and/or preparative RP-HPLC and analysed by analytical RP-HPLC (Figures 2A,C; S1A; S2A,B) and HRMS (Figures 2B,D; S1B; S2C,D). All purified peptides were lyophilised and stored at -80 °C.

Peptide Ac-H4D24(1–37)-$NHNH_2$

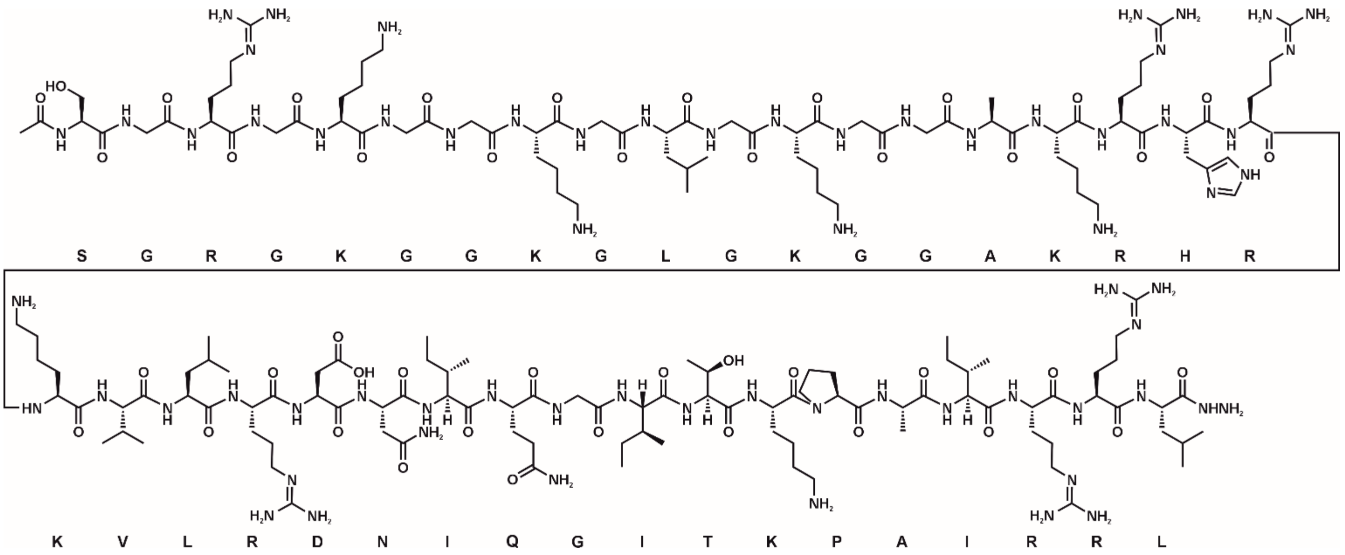

Synthesised as a C-terminal acyl hydrazide with N-terminal acetylation using SPPS as described above with 20% piperidine in DMF as deprotecting agent. Purified sequentially via preparative and then semi-preparative RP-HPLC using gradient LC-A to obtain 2 mg of peptide (≈ 0.50% yield).

Analytical data: RP-HPLC gradient LC-B, r_t = 16.6 min (Figure 2A). HRMS gradient MS-A, calculated mass 3991.38 Da, observed mass 3991.47 Da (Figure 2B).

Peptide Ac-H4isoD24(1–37)-$NHNH_2$ (linear synthesis)

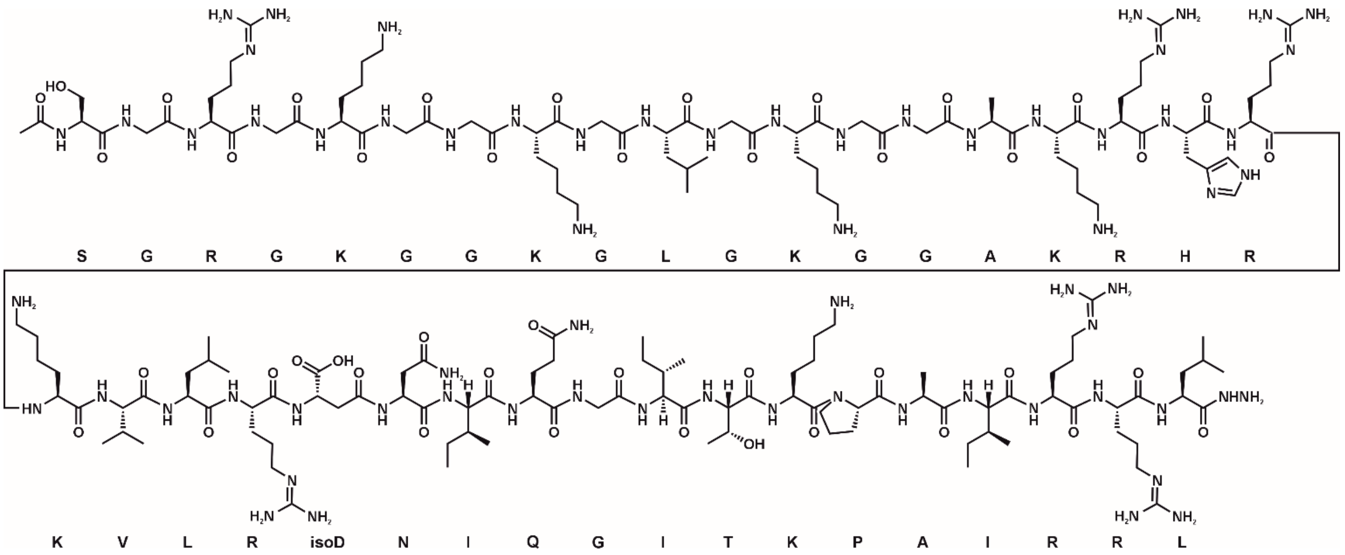

Synthesised as a C-terminal acyl hydrazide with N-terminal acetylation using SPPS as described above with 20% piperidine in DMF as deprotecting agent. Purified sequentially via preparative and then semi-preparative RP-HPLC using gradient LC-A to obtain 2.5 mg of peptide (≈ 0.63% yield, purity < 55% estimated from peak areas in Figure S1A). Due to the high extent of isomerisation, this preparation was not used in subsequent ligations.

Analytical data: RP-HPLC gradient LC-B, r_t = 16.8, 17.2 min. The peak at 17.2 min corresponds to isomerised Ac-H4D24(1–37)-$NHNH_2$ (Figure S1A). HRMS gradient MS-A, calculated mass [M + H^+] 3992.38 Da, observed mass [M + H^+] 3992.34 Da (Figure S1B).

Peptide Ac-H4(1–14)-$NHNH_2$

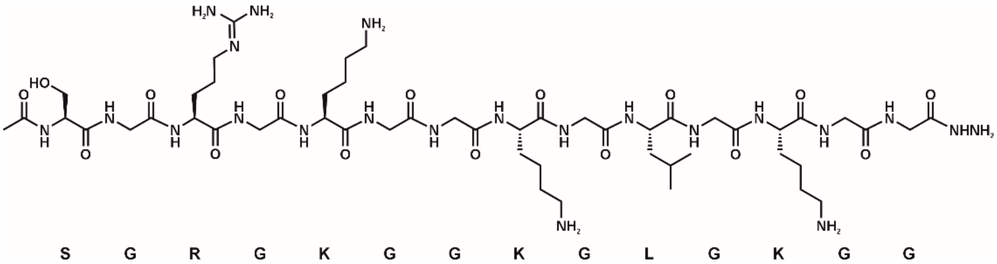

Synthesised as a C-terminal acyl hydrazide with N-terminal acetylation using SPPS as described above with 20% piperidine in DMF as deprotecting agent. Purified sequentially via preparative and then semi-preparative RP-HPLC using gradients LC-A and LC-C, respectively, to obtain 12 mg of peptide (≈4.7% yield).

Analytical data: RP-HPLC gradient LC-B, r_t = 8.8 min (Figure S2A). HRMS by direct injection, calculated mass [M + H^+] 1271.72 Da, [M + Na^+] 1293.71 Da, [M − H^+ + $2Na^+$] 1315.70 Da, observed mass [M + H^+] 1271.71 Da, [M + Na^+] 1293.69 Da, [M − H^+ + $2Na^+$] 1315.67 Da (Figure S2C). The peak at 1451.65 Da corresponds to a side product with N-terminal Fmoc protection in lieu of acetylation (calculated mass 1451.79 Da) which was not detected after subsequent ligation and purification (Figure 2D).

Peptide H4isoD24(A15C, 15–37)-$NHNH_2$

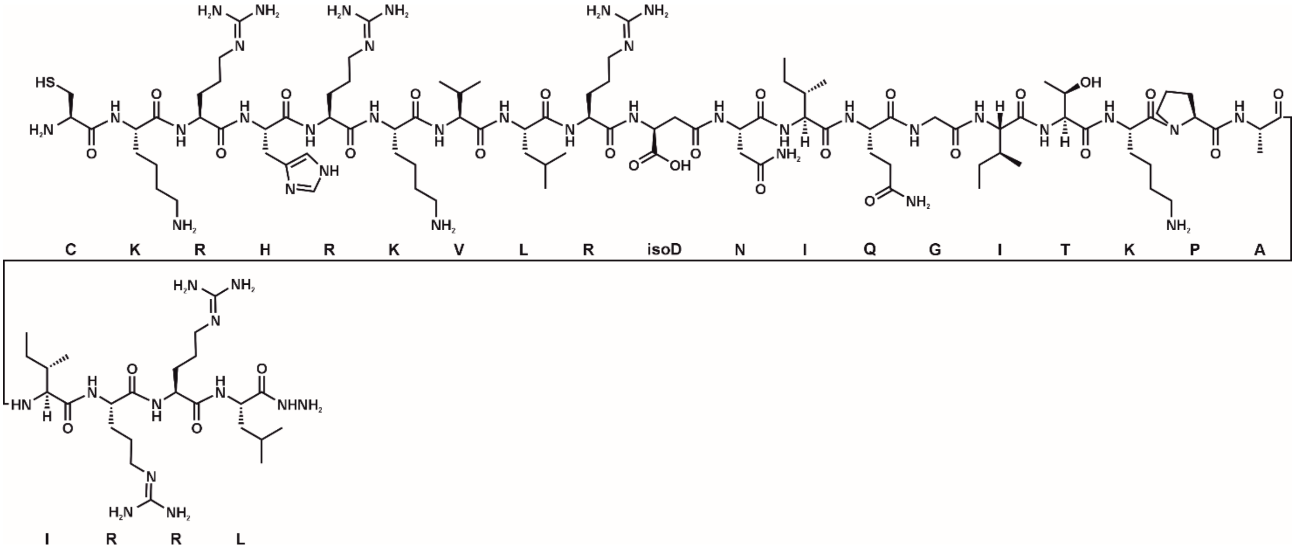

Synthesised as a C-terminal acyl hydrazide using SPPS as described above with 20% piperidine/0.1 M Oxyma in DMF as deprotecting agent. Purified via two sequential rounds of preparative RP-HPLC using gradients LC-D and LC-E, respectively, between which the peptide was dissolved in 7 mL of acetate buffer (pH 3), mixed with 5 mL of 1 M methoxylamine hydrochloride in acetate buffer (pH 4.2), and incubated at RT for 4 hours to remove an N-terminal acetaldehyde adduct. 8.6 mg of peptide was obtained (≈1.5% yield).

Analytical data: RP-HPLC gradient LC-B, r_t = 16.7 min (Figure S2B). HRMS gradient MS-A, calculated mass [M + H^+] 2785.67 Da, observed mass [M + H^+] 2785.68 Da. The peak at 2833.65 Da corresponds to oxidation of C15 to the sulfonic acid (Figure S2D).

Peptide Ac-H4isoD24(1–37)$NHNH_2$ (convergent synthesis)

H4isoD24(1–37) was assembled from peptides H4(1–14) and H4isoD24(A15C, 15–37) via native chemical ligation (NCL) using a protocol adapted from Zheng *et al.*[5] For the ligation, 3.2 mg (2.5 µmol) of lyophilised H4(1–14) were dissolved in 500 µL ligation buffer (200 mM phosphate buffer, 6 M guanidinium chloride, 1.5 mM EDTA, pH 3 and degassed with Argon) to a final concentration of 5 mM. The solution was stirred for 15 minutes at -15°C. To oxidize the peptide acyl hydrazide to the corresponding acyl azide, the peptide solution was cooled to -20°C and 5 µL of 5 M $NaNO_2$ in degassed water was added to a final concentration of 50 mM. The solution was gently stirred for 15 minutes in an ice/NaCl bath (-20 to -15 °C). The thioesterification occurred upon addition of methyl thioglycolate to a final concentration of 150 mM and adjustment of the pH to around 6.8–7.2 using 5 M NaOH. After 10 min of thioester formation at 25°C with shaking at 700 rpm using a Thermomixer C (Eppendorf), 3.5 mg (1.25 µmol) of lyophilised H4isoD24(A15C, 15–37) in ligation buffer at pH 6.8 was added [Before mixing with H4(1–14), the pH of this latter solution was adjusted back to 6.8 with 1 M NaOH after dissolving the peptide]. The reaction pH was adjusted to 6.8–7.2 with 1 M NaOH and the reaction was stirred for 1–2 h. 10 mM TCEP was added approximately every 15 min of reaction for a total of 3 additions and the final concentration of TCEP was approximately 30 mM over the course of ligation. To monitor the reaction progress, 2 µL of the mixture were withdrawn at specified intervals and diluted to 40 µL with ligation buffer at pH 3 and analysed by RP-HPLC and HRMS.

When the ligation was almost complete (≈ 1 h), desulfurisation of C15 to recover the native alanine was performed in the same reaction tube without prior purification. TCEP desulfurisation buffer (0.5 M TCEP, 6 M guanidinium chloride, 0.2 M phosphate, pH 7) was added to the reaction to a final TCEP concentration of 0.25 M, and the desulfurization reaction was initiated by addition of VA-044 and glutathione to final concentrations of 30 mM and 40 mM, respectively. VA-044 and GSH were pre-dissolved to 1 M stock solutions with desulfurisation buffer and adjusted to pH 7 using NaOH. After addition of these reagents, reaction pH was monitored and adjusted to around 6.6 with 1 M NaOH if necessary. The reaction was incubated overnight at 37 °C with shaking at 700 rpm using a Thermomixer C (Eppendorf) and monitored by RP-HPLC and HRMS analysis.

When the desulfurisation was complete, the peptide was purified via semi-preparative RP-HPLC using gradient LC-F to obtain 2.2 mg of peptide (≈ 37% yield).

Analytical data: RP-HPLC gradient LC-B, r_t = 16.5 min (Figure 2C). HRMS gradient MS-A, calculated mass 3991.38 Da, observed mass 3991.47 Da. Peaks at 459.79 Da and 551.54 Da are the 6+ and 5+ charge states of H4isoD24(A15C, 15–37) with loss of hydrazine, and peaks at 473.12 Da and 567.54 Da are the 6+ and 5+ charge states of H4isoD24(A15C, 15–37) with oxidation of C15 to the sulfonic acid (Figure 2D).

Expression and purification of recombinant histone proteins

Recombinant human wild-type core histones H2A, H2B, and H3(C96A, C110A), H4 were expressed and purified as previously described.[6] Histone expression plasmids (provided by the lab of Tom Muir, Princeton University) were transformed into BL21(DE3) cells and 1 L of culture per histone was grown at 37°C to OD_{600} around 0.5. Expression was induced by addition of 0.8 mM IPTG for 2–3 hours. The cell cultures were centrifuged, and the pellets were stored in -80°C.

Cell pellets were lysed by sonication in lysis buffer (50 mM Tris pH 7.6, 100 mM NaCl, 1 mM EDTA, 1 mM 2-mercaptoethanol) for 4 minutes in cycles of 15s ON, 15s OFF at 40% amplitude at 4°C. The lysate was then spun at 4°C for 30 minutes at 27,200 xg. The supernatant was decanted, and the pellet was washed in 10 mL lysis buffer + 1% v/v Triton-X and then centrifuged at 4°C for 10 minutes at 27,200 xg. The supernatant was again decanted, and another Triton wash was performed in 10 mL lysis buffer + 1% v/v Triton-X. The sample was centrifuged at 4 °C for 10 minutes at 27,200 xg. The supernatant was decanted, and a final wash was performed without detergent in 10 mL of lysis buffer. The sample was again spun at 4°C for 10 minutes at 27,200 xg. The supernatant was decanted and the resulting pellet containing histone proteins in inclusion bodies was resuspended in 8 mL of buffer containing 6 M guanidinium chloride, 20 mM Tris, 1 mM EDTA, 100 mM NaCl, 1 mM DTT, pH 7.6. This resuspension buffer was added to the pellet and nutated at 4 °C for 2 hours and then centrifuged for 30 min at 30,000 xg. The supernatant was removed and dialyzed in 1 L of 6.2 M Urea, 10 mM Tris, 1 mM EDTA, 100 mM NaCl, 1 mM DTT, pH 7.6. The next day, the dialyzed supernatant was injected onto a 5 mL HiTrap HP SP column (Cytiva) on an ÅKTA pure FPLC (Cytiva) at 4 °C and eluted at 1.5 mL/min by 6.2 M Urea, 10 mM Tris, 1 mM EDTA, 1 mM DTT, pH 7.6 buffer with a salt gradient of 0.1–1 M NaCl. Eluted histone proteins were dialyzed in 1 L water + 0.1% acetic acid, lyophilised, and stored at -80 °C. After enough materials were obtained, the pooled materials for each histone were purified via preparative RP-HPLC using gradient LC-G to obtain 31 mg/3.1 L, 8.7 mg/4.1 L, and 27 mg/2 L for histones H2B, H2A, and H3, respectively. These purified histones were lyophilised and stored at -80 °C.

Analytical data: RP-HPLC gradient LC-H, r_t = 21.6 min (H2A, Figure S7A), r_t = 21.4 min (H2B, Figure S7B), r_t = 24.8 min (H3, Figure S7C). HRMS gradient MS-B: Calculated average mass 13964 Da, observed mass 13964 Da (H2A, Figure S8A); calculated average mass 13759 Da, observed mass 13759 Da (H2B, Figure S8B); calculated average mass 15209 Da, observed mass 15209 Da (H3, Figure S8C).

Expression and purification of truncated H4 38−102 (A38C)

The C-terminal fragment of histone H4 [H4(A38C, 38–102)] with N-terminal fusion to a His_6-SUMO tag was recombinantly expressed in Rosetta (DE3) cells. Bacterial cultures were grown in 16x0.85 L LB medium (with 100 µg/mL ampicillin and 35 µg/mL chloramphenicol) at 37°C to an OD_{600} of 0.6–0.8. Protein expression was induced with 0.25 mM IPTG, and cells were incubated at 37°C for 2 h. Cells were harvested by centrifugation (4200 xg, 4°C, 15 min) and stored at -80°C. H_6-SUMO-H4(A38C, 38–102) is insoluble and expresses as inclusion bodies. Thus, cell pellets were lysed and processed as for recombinant histones to extract and re-solubilize the inclusion bodies. After solubilisation and centrifugation to remove insoluble material, the soluble fraction [15 mL resuspension buffer (6 M

guanidinium chloride, 20 mM Tris, 1 mM EDTA, 100 mM NaCl, 1 mM DTT, pH 7.6) with material from 4x0.85 L cultures] was applied to 4 mL Ni-NTA resin (Qiagen) in a plastic column which was previously equilibrated in resuspension buffer. The supernatant was left to bind the resin overnight with gentle shaking at 4°C. The next day, the flow-through was collected and the resin washed with 3 column volumes (CV) of resuspension buffer and 3 CV of urea wash buffer (6 M Urea, 50 mM Tris-HCl, 1.5 mM DTT, pH 7.7). The protein was then eluted with 4x1.5 CV urea elution buffer (6 M Urea, 50 mM Tris-HCl, 1.5 mM DTT, 250 mM imidazole, pH 7.7). Washing and elution fractions were analysed by SDS-PAGE (15% acrylamide) and fractions containing His-SUMO-H4(38–102) were pooled. ~0.2 mg Ulp1 were added to the pooled fractions and the mixture was dialyzed against 1 L dialysis buffer (1 M Urea, 150 mM NaCl, 75 mM Tris-HCl, 25 mM L-Arg, 5 mM L-Cys, 2 mM DTT, pH 7.5) overnight at 4°C. This procedure allows the refolding and cleavage of the His_6-SUMO tag from the His_6-SUMO-H4(A38C, 38–102) fusion protein. The next day, the dialysed mixture was collected and centrifuged at 15,000 xg for 15 min at 4 °C. The resulting pellet was dissolved with 2 mL resuspension buffer, and both the resuspended pellet and supernatant were treated with 5 mM TCEP. Crude H4(A38C, 38–102) in both fractions was purified via semi-preparative RP-HPLC using gradient LC-I to obtain 2.7 mg protein. The pure construct was lyophilised and stored at -80 °C.

Analytical data: RP-HPLC gradient LC-H, r_t = 23.1 min (Figure S5A). HRMS gradient MS-B, calculated mass [M + H^+] 7344.98 Da, observed mass [M + H^+] 7344.97 Da (Figure S5B).

Expression and purification of Chd1

A StrepTag-MBP-TEV-Chd1 (MBP = Maltose-binding protein; TEV = TEV protease recognition site) construct was cloned into pACEBac1 (Geneva Biotech) and baculovirus particles were generated as per the manufacturer's instructions. 1 L cultures of Sf9 cells were grown to 2–2.5 x 10^6 cells/mL. Subsequently, the cells were infected with baculovirus, and the cultures were incubated for 3 days at 27 °C before harvesting through centrifugation (1500 xg, 4° C for 20 min). Supernatants were discarded and pellets were resuspended in phosphate-buffered saline (PBS) containing protease inhibitors (Roche) (10 mL PBS per 1 L of culture), flash frozen, and stored at -80 °C.

12–15 g of frozen pellets were thawed at room temperature in 36 mL of lysis buffer [200 mM KCl, 2 mM DTT, 100 mM Tris-HCl (pH 7.5), 50 mM MgOAc, 0.1% NP-40, with added Protease inhibitor cocktail (Roche), 1mM PMSF and 20 µL DNaseI (NEB)]. Cell pellets were redispersed by stirring with a magnetic stir bar and then kept on ice for lysis. The resulting lysate was centrifuged for 35 min at 35,000 rpm at 4 °C (Ti70 rotor, Beckman Coulter). The supernatant was further filtered through a 5 µm syringe filter (Millex, Millipore). The cleared lysate was loaded onto a Strep-Trap column (GE, AKTA system), which was previously pre-equilibrated with lysis buffer. The column was washed with storage buffer [200 mM KCl, 10 mM HEPES pH 7.6, 50 mM MgOAc, and 5 mM β-mercaptoethanol (β-Me)] and the protein was eluted with 5 x column volumes (CV) of storage buffer containing 2.5 mM desthiobiotin. Fractions containing Chd1 were identified by SDS-Page and pooled, and then concentrated to ~500 µL total volume using 10 kDa molecular weight cutoff (MWCO) centrifugal filters (Amicon). The protein concentration was determined using UV spectroscopy. The MBP tag was subsequently removed by TEV protease digestion

at 4 °C. Chd1 was finally purified by size-exclusion chromatography (SEC) using a Superose6 10/300 GL column (GE healthcare) in storage buffer with a flow-rate of 0.4 mL/min (Figure S12B). Fractions were analysed using SDS-PAGE (Figure S12C), clean fractions were pooled, concentrated (Amicon 10 kDa MWCO filter), and protein concentrations were determined using UV spectrophotometry.

Expression and purification of Suv4-20h1

M. musculus Suv4-20h1(61-327) was expressed as a GST-fusion protein in *E. coli* as described in ref [3]. The GST-fusion protein was purified on glutathione Sepharose affinity resin then cleaved from the GST tag by incubation with rhinovirus 3C protease. The protein was further purified by SEC (Superdex S75, Cytiva). The purification buffer contained 20 mM HEPES (pH 8.0), 500 mM NaCl, and 2 mM β-Me.

Native chemical ligation and desulfurisation of H4(iso)D24(1–102)

<u>The ligation of H4isoD24(1–37) to H4(A38C, 38–102):</u> 1.1 mg (0.275 µmol) of lyophilised H4isoD24(1–37) acyl hydrazide (synthesised convergently) was used to ligate with 1.1 mg (0.15 µmol) of lyophilised H4(A38C, 38–102) as described above for the ligation of H4isoD24(1–37).

<u>The ligation of H4D24(1–37) to H4(A38C, 38–102):</u> 0.7 mg (0.175 µmol) of lyophilised H4D24(1–37) acyl hydrazide was used to ligate with 0.6 mg (0.082 µmol) of lyophilised H4(A38C, 38–102) as described above for the ligation of H4isoD24(1–37).

<u>For both ligations:</u> Ligations and desulfurisation reactions were carried out as for the convergent synthesis of the H4isoD24(1–37) acyl hydrazide, though the ligations required 5–6 h to reach completion due to the sterically-demanding leucine at position 37 (Figure S6). Purified via semi-preparative RP-HPLC using gradient LC-I to obtain 0.36 mg (≈25% yield) and 0.42 mg (≈25% yield) for H4D24(1–102) and H4isoD24(1–102), respectively.

Analytical data: RP-HPLC gradient LC-H, r_t = 22.4 min (H4D24, Figure 2E), r_t = 22.4 min (H4isoD24, Figure 2G). HRMS gradient MS-B: Calculated average mass 11278.2 Da, observed mass 11278.1 Da, the peak at 11121.0 Da is a -Arg_1 truncation (H4D24, Figure 2F); calculated average mass 11278.2 Da, observed mass 11278.1 Da (H4isoD24, Figure 2H).

PIMT methylation of H4D24(1–37) and H4isoD24(1–37) peptides

The reactions below were mixed on ice in low-binding microcentrifuge tubes (Eppendorf).

1) **Reaction 1 (800 µL):** 100 mM sodium phosphate (pH 6.8), 1 mM EGTA, 20 µM AdoMet, 1 µM PIMT, 2 µM H4D24(1–37) acyl hydrazide
2) **Reaction 2 (800 µL):** 100 mM sodium phosphate (pH 6.8), 1 mM EGTA, 20 µM AdoMet, 1 µM PIMT, 2 µM H4isoD24(1–37) acyl hydrazide
3) **Reaction 3 (100 µL):** 100 mM sodium phosphate (pH 6.8), 1 mM EGTA, 20 µM AdoMet, 2 µM H4D24(1–37) acyl hydrazide
4) **Reaction 4 (100 µL):** 100 mM sodium phosphate (pH 6.8), 1 mM EGTA, 20 µM AdoMet, 2 µM H4isoD24(1–37) acyl hydrazide

Reactions 3 and 4 were used as 0 min timepoints and were immediately mixed with 20 µL of 0.3 M phosphoric acid. Reactions 1 and 2 were incubated on a Thermomixer C (Eppendorf) at 30 °C with shaking at 550 rpm. At 1, 2, 5, 10, 15, 30, and 60 min, 100 µL aliquots from reactions 1 and 2 were quenched by mixing with 20 µL of 0.3 M phosphoric acid and incubating on ice for a least 10 min. All timepoints were then centrifuged at 14,000 xg for 10 min at 4 °C. 3 µL (5 pmol peptide) of each timepoint were analysed by HRMS with gradient MS-C. The resulting mass spectra were deconvoluted using the MaxEnt1 algorithm through the UNIFI software platform (Waters) with the following parameters:

- Input range: 350–900 Da
- Output range: 3500–4500 Da
- Output resolution: 0.01 Da
- Peak width model: Manual (Start peak width = 0.0221; End peak width = 0.0436)
- Minimum left and right intensities: 30%
- Charge carrier: Hydrogen
- Iterate to convergence with noise autoscaling

The peaks of the deconvoluted spectra were then centred using the same software with the following parameters:

- Top of each peak to use: 80%
- Minimum peak half width: 4
- Report using: Area

These centred deconvolutions yielded zero-charge mass spectra (Figure S4A,B). For the H4isoD24(1-37) reaction, the intensities of peaks with intensities greater than 104,000 (coloured peaks in Figure S4) in the succinimidyl (Expected mass = 3973.37 Da), unmethylated (Expected mass = 3991.38 Da), and methylated (Expected mass = 4005.40 Da) peptide isotope clusters were then summed and normalised to total peptide signal to yield relative intensities of each peptidic species. Assuming the reaction diagram below (Scheme S1), the following system of ordinary differential equations was solved numerically using a custom Python script, where A = isoAsp peptide, B = methyl-isoAsp peptide, C = succinimidyl peptide, and D = Asp peptide:

$$\frac{d[\mathrm{A}]}{dt} = k_3[\mathrm{C}](t) - k_1[\mathrm{A}](t), \tag{S1}$$

$$\frac{d[\mathrm{B}]}{dt} = k_1[\mathrm{A}](t) - k_2[\mathrm{B}](t), \tag{S2}$$

$$\frac{d[\mathrm{C}]}{dt} = k_2[\mathrm{B}](t) - (k_3 + k_4)[\mathrm{C}](t), \text{ and} \tag{S3}$$

$$\frac{d[\mathrm{D}]}{dt} = k_4[\mathrm{C}](t). \tag{S4}$$

Performing a non-linear, least-squares global fit of the obtained solutions to Equations S1–S4 (noting that the solutions for S1 and S4 must be summed as the isoAsp and Asp peptides are indistinguishable by mass) to the relative amounts of each peptidic species over time yielded the following rate constants for the H4isoD24(1–37) PIMT reaction [with 95% confidence interval (CI)]: $k_1 = 0.079 \pm 0.003$ min^{-1}, $k_2 = 0.011 \pm 0.002$ min^{-1}, $k_3 = 0.00 \pm 0.01$ min^{-1}, and $k_4 = 0.008 \pm 0.006$ min^{-1} (Figure S4C).

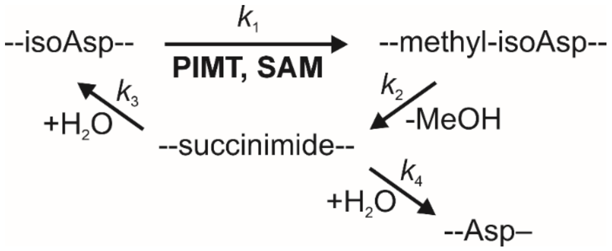

Scheme S1. Reaction diagram for PIMT-mediated methylation of isoAsp containing peptides.

Octamer assembly

Histone octamers were prepared via established methods.[7, 8] Briefly, the four lyophilized core histones were separately dissolved to ≈2 mg/mL in octamer unfolding buffer (20 mM Tris, 6 M guanidinium hydrochloride, 0.5 mM EDTA, 10 mM DTT, pH 7.5 at 4°C), and the precise concentration of the solutions were determined by UV absorbance at 280 nm using the calculated extinction coefficients. The four histone proteins were combined with a ratio of H2A:H2B:H3:H4 = 1.1 : 1.1 : 1 : 1, as the excess of H2A/H2B over H3/H4 prevents formation of H3/H4 tetramers that are difficult to remove. The final concentration of the mixture was adjusted to 1 mg/mL by dilution with additional octamer unfolding buffer. The solution was dialyzed against octamer refolding buffer (10 mM Tris, 2 M NaCl, 0.5 mM EDTA, 1 mM DTT, pH 7.5 at 4 °C) at 4°C, and the octamers were purified from aggregates, H3/H4 tetramers, and H2A/H2B dimers by SEC using a Superdex 200 10/300 column (GE Healthcare Life Sciences) with octamer refolding buffer at a flow rate of 0.4 mL/min (Figure S9A,B). The fractions were analysed by SDS-PAGE (15% acrylamide), and pure fractions were combined, concentrated (Vivaspin, 10 kDa MWCO) to a final concentration of 29.3 µM and 28.8 µM for H4D24- and H4isoD24-containing octamers, respectively, and stored in 50% glycerol at -20 °C. SDS-PAGE (15% acrylamide) confirmed equimolar presence of each histone in the purified octamer preparations (Figure S9C).

MN assembly

A DNA template with an offset “601” sequence was mixed with H4D24- or H4isoD24-containing octamers with octamer:DNA ratios of 1, 1.5, or 2 in assembly buffer (2 M NaCl, 10 mM Tris, 1 mM EDTA, pH 7.5 at 25 °C). Each assembly mixture was dialysed (Slide-A-Lyzer MINI dialysis units, 10 kDa MWCO; Thermo Scientific) against 100 mL of assembly buffer at 4 °C. The NaCl concentration of the dialysate was gradually lowered to 10 mM using assembly buffer without NaCl and a peristaltic pump at 3 mL/min. Native PAGE (12% acrylamide) was used to ascertain the quality of MN assembly (Figure S12A), and MNs assembled with an octamer:DNA ratio of 1.5 were chosen for remodelling assays.

Assembly of 12-mers

12-mer nucleosome arrays were assembled by salt-gradient dialysis of purified H4D24- or H4isoD24-containing octamers and a 12x“601” DNA template in the presence of MMTV buffer DNA as per previously published methods.[7] The resulting arrays were purified by Mg^{2+}-mediated self-association: Mg^{2+} was added to the 12-mer assembly mixtures to the desired concentration (see note at end of section), after which the mixtures were incubated on ice for 20 min and then centrifuged for 10 min at 15,000g at 4 °C. The resulting pellet was resuspended by adding ice-cold TEK buffer (10 mM Tris, 0.1 mM EDTA, 10 mM KCl, pH 7.5 at 4 °C). Concentrations were quantified by DNA absorbance at 260 nm, and typical yields were 75–90% (Concentration of resuspended pellet normalised by summed concentrations of resuspended pellet and supernatant). Purified 12-mers were visualised by native agarose/polyacrylamide gel electrophoresis (APAGE) [2% acrylamide, 1% agarose; 0.25x TAE, 120 V, 10% sucrose as densifying agent, SybrGold (ThermoFisher Scientific) staining] (Figures S10, S20).

Note that, depending on the researcher, final Mg^{2+} concentrations used for purification ranged between 3–5 mM and ratios of octamer to individual “601” site ranged between 1.9–3.85. Therefore, all large-scale 12-mer preparations were preceded by small-scale test assemblies and arrays were only used after validation by restriction digest (described in the following section).

Quality checking of 12-mers

Array integrity was assessed via restriction digest by ScaI or MNase. The former was performed for 4 h at 37 °C in ScaI digest buffer [10 mM Tris (pH 7.5 at 4 °C) containing 0.1 M KCl, 0.5 mM $MgCl_2$ and 1 mM DTT], with either: (1) 1pmol of “601” sites and 0.5 µL ScaI-HF (NEB) in a total volume of 3.75 µL; or (2) 50 ng of DNA and 0.5 µL ScaI-HF (NEB) in a total volume of 10 µL. Digested products were analysed by native APAGE [2% acrylamide, 1% agarose; 0.25x TAE, 120 V, 10% sucrose as densifying agent, SybrGold (ThermoFisher Scientific) staining]. MNase digestion was performed with 2 pmol of 601 sites and 0.3 µL MNase (NEB) in 10 µL of 50 mM Tris-HCl, pH 7.9 at RT, containing 5 mM $CaCl_2$ on ice. The reaction was quenched with 10 µL SDS buffer (0.4 M NaCl, 0.2% (w/v) SDS, 20 mM EDTA) after 20 s, and the digested DNA was isolated with a PCR purification kit (Qiagen) and analysed by agarose gel electrophoresis (1% agarose, 1x TAE).

For appropriately saturated 12-mers, ScaI digestion should predominantly produce MNs (Figures S10A,B; S20; S24; S25; and S29) and MNase digests should produce 12-species ladders (Figure S10C).

Mg^{2+}-mediated self-association of H4(iso)D24 12-mers

The Mg^{2+}-mediated self-association assay was performed at approximately 0.3 µM of 601 sites in TEK buffer. Briefly, 6.5 µL of 0.6 µM “601” sites were mixed with 6.5 µL of $MgCl_2$ at double the desired final concentration of Mg^{2+} and then incubated for 15 min at 20°C. Samples were centrifuged at 15,000 xg for 12 min at 20°C, and the supernatants were transferred to a fresh tube. The A_{260} of the supernatant for each sample was measured and normalised to the A_{260} of the supernatant at 0 mM Mg^{2+}. The assay was performed from 0 to 5 mM final $MgCl_2$ in 0.5 mM increments. Self-association profiles were obtained by plotting the fractions of the array in the supernatant against final Mg^{2+} concentrations. Two technical

replicates were averaged for each sample, and the number of biological replicates per concentration of Mg^{2+} is described in the caption for Figure 3. Data were fit using the "[Inhibitor] vs. response – Variable slope (four parameters)" function in Prism (GraphPad).

Chd1 remodelling of H4(iso)D24 MNs

Remodelling reactions were performed in 20 µL volumes containing 20 nM H4D24- or H4isoD24-containing MNs, 0.3 eq. of Chd1 to MNs, 1 mM ATP, and reaction buffer (20 mM Tris, 300 mM KCl, 6 mM $MgCl_2$, 0.2 mg/mL BSA, pH 8 at 25 °C). Reaction mixtures were incubated at 30 °C for 0, 5, 12, 20, 30, 60, or 90 min, and then 500 ng of plasmid DNA was added to quench each reaction via competitive binding to Chd1. Each quenched timepoint was visualised by native PAGE (Figure S13), and the remodelled fraction was quantified as per Figure S14.

Data were fit using the "One-phase association" function in Prism (GraphPad) with remodelling fraction constrained to 0 at t = 0 min.

Set8 monomethylation on H4(iso)D24(1-37) peptides

Set8 was diluted with reaction buffer (25 mM Tris, 100 mM NaCl, 3 mM DTT, pH 8.0 at RT) to 5 pmol/µL. Separate 100 µL reactions were then prepared using reaction buffer, 0.1 µM Set8, 100 µM AdoMet, and 1 µM, 2 µM, 4 µM, 8 µM, 20 µM, or 40 µM of H4D24(1-37) or H4isoD24(1-37) acyl hydrazide peptide. Each reaction mixture was incubated on a Thermomixer C (Eppendorf) at 30 °C with shaking at 550 rpm. At the timepoints indicated in Figure S17A–F, 15 µL aliquots were removed from the reaction mixtures and quenched with 1% formic acid in water. The quenched aliquots were then appropriately diluted with HRMS solvent C before HRMS analysis with gradient MS-A.

For each reaction, mass spectra composing the single observed peak in the resulting total ion chromatograms (TICs) were summed together (Figure S16). Let S_{me0} and S_{me1} represent the intensities of the highest peaks in the +7 charge state isotope clusters of the unmethylated (H4K20me0; Expected 7+ m/z = 571.20) and monomethylated (H4K20me1; Expected 7+ m/z = 573.20) species. The relative intensity (assumed to be proportional to relative amount) of H4K20me1 was then calculated via [100% × $S_{me1}/(S_{me0} + S_{me1})$]. The reaction velocity at each peptide concentration was derived using the slopes given by linear regressions of relative intensity vs. time (Figure S17A–F), and the resulting Michaelis-Menten kinetic curves (Figure S17G) were fit using the "Michaelis-Menten" function in Prism (GraphPad). Approximation of k_{cat}/K_m was performed via linear regression in Prism of peptide concentrations 1–8 µM (Figure S17H) and using the given Set8 concentration of 0.1 µM.

Set8/Suv4-20h1 H4K20 dimethylation cascade on H4(iso)D24(1–37) peptides

Three replicates each (same peptide and enzyme stocks, but prepared independently) of the following two reactions were prepared on ice in low-binding microcentrifuge tubes (Eppendorf).

1) **Reaction 1 (21.111 µL):** 25 mM Tris (pH 8 at RT), 100 mM NaCl, 3 mM DTT, 500 µM AdoMet, 1 µM Set8, 1 µM Suv4-20h1, 0.9 µM H4D24(1–37) acyl hydrazide

2) **Reaction 2 (21.111 µL):** 25 mM Tris (pH 8 at RT), 100 mM NaCl, 3 mM DTT, 500 µM AdoMet, 1 µM Set8, 1 µM Suv4-20h1, 0.9 µM H4isoD24(1–37) acyl hydrazide

The 0 min timepoints were generated by immediately quenching 2.88 µL aliquots of each reaction with 10.12 µL of 0.064 M phosphoric acid. These reactions were then incubated on a Thermomixer C (Eppendorf) at 30 °C with shaking at 550 rpm. At 10, 20, 30, 45, 60, and 90 min, 2.88 µL aliquots were removed from each reaction and quenched with 10.12 µL of 0.064 M phosphoric acid. All timepoints were then centrifuged at 17,000 xg for 5 min at RT, after which 10 µL (2 pmol peptide) of each timepoint was analysed by HRMS with gradient MS-D. Mass spectra from the resulting TICs were summed over the ranges denoted in Figure S18, and then these summed spectra were deconvoluted using the MaxEnt1 algorithm through the MassLynx software platform (Waters) with the following parameters:

- Input range: 350–900 Da
- Output range: 3500–4500 Da
- Output resolution: 0.1 Da/channel
- Damage model: Uniform Gaussian, with width at half height of 0.037 Da
- Minimum left and right intensity ratios: 33%
- Iterated to convergence

The peaks of the deconvoluted spectra were then centred using the same software with the following parameters:

- Min peak width at half height (channels): 2
- Centre method: Median
- Create centred spectrum using areas

These centred deconvolutions yielded zero-charge mass spectra (Figure S19). The intensities of the first nine peaks (coloured peaks in Figure S19) in the unmethylated (H4K20me0; Expected mass = 3991.38 Da), monomethylated (H4K20me1; Expected mass = 4005.40 Da), and dimethylated (H4K20me2; Expected mass = 4019.42 Da) peptide isotope clusters were then summed and normalised to total peptide signal to yield relative intensities of each peptidic species. Finally, background subtraction was performed by (1) subtracting the relative intensities of the H4K20me1 and H4K20me2 species obtained at 0 min from the relative intensities of these species found at all timepoints, and (2) adding the relative intensities of H4K20me1 and H4K20me2 species obtained at 0 min to the relative intensity of the H4K20me0 species found at all timepoints (In this case, background subtraction could not be performed using absolute intensities as for the 12-mer reactions described below because of the high variance in signal intensities). Note that this ensures a relative intensity of unity for the H4K20me0 species at 0 min.

As indicated by the data shown in Figure 5C, the methylation of the peptides by Set8 and Suv4-20h1 did not go to completion. To obtain meaningful fits to the data, we therefore added a correction factor *p* to Equations S8–S10. Briefly, the concentration of H4K20me0 peptide [A] is assumed to be the sum of the concentrations of a static, unreactive population $[A]_U = (1-p)[A]_0$ and a time-dependent reactive population

$[A]_R$, where $[A]_0$ represents the initial concentration of unmethylated peptide. Given that the time evolution of $[A]_R$ is the same as in Equation S8 and its initial value must be $[A]_{R,0} = p[A]_0$, we then have

$$[A] = [A]_R + [A]_U = [A]_R e^{-k_{1,obs}t} + [A]_U = p[A]_0 e^{-k_{1,obs}t} + (1-p)[A]_0.$$

Normalising by $[A]_0$ yields the corrected version of Equation S8,

$$\frac{[A]}{[A]_0} = pe^{-k_{1,obs}t} + (1-p). \quad \text{(S5)}$$

We assume that the populations of H4K20me1 and H4K20me2 peptides—concentrations given by [B] and [C], respectively—are wholly reactive, and so the corrected equivalents of Equations S9 and S10 are just proportional to *p*:

$$\frac{[B]}{[A]_0} = p\left(\frac{k_{1,obs}}{k_{2,obs} - k_{1,obs}}\right)\left(e^{-k_{1,obs}t} - e^{-k_{2,obs}t}\right), \text{ and} \quad \text{(S6)}$$

$$\frac{[C]}{[A]_0} = p\left[1 + \left(\frac{k_{1,obs}e^{-k_{2,obs}t} - k_{2,obs}e^{-k_{1,obs}t}}{k_{2,obs} - k_{1,obs}}\right)\right]. \quad \text{(S7)}$$

Non-linear, least-squares global fits of the peptide reaction data above to Equations S5–S7 were then performed using a custom Python script to generate the estimated rate constants and correction factors reported in the main text.

Set8/Suv4-20h1 H4K20 dimethylation cascade on H4(iso)D24 12-mers

Three replicates each [same octamer, DNA, and enzyme stocks, but independently assembled 12-mers (Figure S20)] of the following two reactions were prepared on ice in low-binding microcentrifuge tubes (Eppendorf).

3) **Reaction 1 (21.111 µL):** 25 mM Tris (pH 8 at RT), 100 mM NaCl, 3 mM DTT, 500 µM AdoMet, 1 µM Set8, 1 µM Suv4-20h1, 0.45 µM “601” sites (0.9 µM H4D24) of H4D24-containing 12-mer
4) **Reaction 2 (21.111 µL):** 25 mM Tris (pH 8 at RT), 100 mM NaCl, 3 mM DTT, 500 µM AdoMet, 1 µM Set8, 1 µM Suv4-20h1, 0.45 µM “601” sites (0.9 µM H4isoD24) of H4isoD24-containing 12-mer

The 0 min timepoints were generated by immediately quenching 2.88 µL aliquots of each reaction with 10.12 µL of a solution containing 0.64% formic acid and 3.85 M guanidinium chloride. These reactions were then incubated on a Thermomixer C (Eppendorf) at 30 °C with shaking at 550 rpm. At 10, 20, 30, 45, 60, and 90 min, 2.88 µL aliquots were removed from each reaction and quenched with 10.12 µL of a solution containing 0.64% formic acid and 3.85 M guanidinium chloride. The unquenched remnants of each reaction mixture were then subjected to ScaI digestion and native APAGE as described above to verify that 12-mers remained intact throughout the reactions (Figure S24).

All timepoints were then centrifuged at 17,000 xg for 5 min at RT, after which 10 µL (2 pmol H4) of each timepoint was analysed by HRMS with gradient MS-E. Both a sampling cone voltage of 100 V and a

desolvation temperature of 350 °C were critical for optimal signal-to-noise. Mass spectra from the resulting TICs were summed over the ranges denoted in Figure S21, and then these summed spectra were deconvoluted using the MaxEnt1 algorithm through the MassLynx software platform (Waters) with the following parameters (Figure S22):

- Input range: 500–3000 Da
- Output range: 10,000–15,000 Da
- Output resolution: 1 Da/channel
- Damage model: Uniform Gaussian, with width at half height of 0.5 Da
- Minimum left and right intensity ratios: 33%
- Iterated to convergence

The deconvoluted peak intensities at the expected average masses of H4 proteoforms with H4K20me0 (11,278 Da), H4K20me1 (11,292 Da), and H4K20me2 (11,206 Da) were used directly and normalised to their sum to yield relative intensities of each proteoform (Centring of the deconvoluted spectra as performed for the peptide reactions described above was too inaccurate at this level of resolution). Finally, background subtraction was performed by (1) subtracting the absolute intensities of the H4K20me1 and H4K20me2 proteoforms obtained at 0 min from the absolute intensities of these proteoforms found at all timepoints, and (2) adding the absolute intensities of H4K20me1 and H4K20me2 proteoforms obtained at 0 min to the absolute intensity of the H4K20me0 proteoform found at all timepoints. Note that this ensures a relative intensity of unity for the H4K20me0 proteoform at 0 min.

Given $[\mathrm{A}]_0$ as the initial concentration of H4K20me0 and assuming the sequential reaction in Figure 5B, the integrated rate equations for the relative amounts of H4K20me0 ([A]), H4K20me1 ([B]), and H4K20me2 ([C]) are given by

$$\frac{[\mathrm{A}]}{[\mathrm{A}]_0} = e^{-k_{1,\mathrm{obs}}t}, \tag{S8}$$

$$\frac{[\mathrm{B}]}{[\mathrm{A}]_0} = \left(\frac{k_{1,\mathrm{obs}}}{k_{2,\mathrm{obs}} - k_{1,\mathrm{obs}}}\right)\left(e^{-k_{1,\mathrm{obs}}t} - e^{-k_{2,\mathrm{obs}}t}\right), \text{ and} \tag{S9}$$

$$\frac{[\mathrm{C}]}{[\mathrm{A}]_0} = 1 + \left(\frac{k_{1,\mathrm{obs}}e^{-k_{2,\mathrm{obs}}t} - k_{2,\mathrm{obs}}e^{-k_{1,\mathrm{obs}}t}}{k_{2,\mathrm{obs}} - k_{1,\mathrm{obs}}}\right). \tag{S10}$$

Non-linear, least-squares global fits of these data to Equations S8–S10 were then performed using a custom Python script to generate the estimated rate constants reported in the main text.

Set8 monomethylation of H4(iso)D24 12-mers

A new singleton of H4D24-containing 12-mer and two independent duplicates (“independence” defined in the previous section) each of 12-mers containing 2:1 or 1:1 ratios of H4D24- and H4isoD24-containing octamers were assembled (Figure S25) and subjected to the same reaction conditions and HRMS

analyses (Figure S26,S27) as for the dimethylation assays, but now without Suv4-20h1. In this case, the integrated rate equation for the relative amount of H4K20me1 ([B]) is now given by

$$\frac{[\mathrm{B}]}{[\mathrm{A}]_0} = 1 - e^{-k_{1,\mathrm{obs}}t}. \tag{S11}$$

As for the dimethylation assays, non-linear, least-squares global fits of the data to Equations S8 and S11 were performed using a custom Python script to generate estimates of $k_{1,\mathrm{obs}}$ (Figure S28). As before, the unquenched remnants of each reaction mixture were finally subjected to ScaI digestion and native APAGE to verify that 12-mers remained intact throughout the reactions (Figure S29).

Supplementary Figures

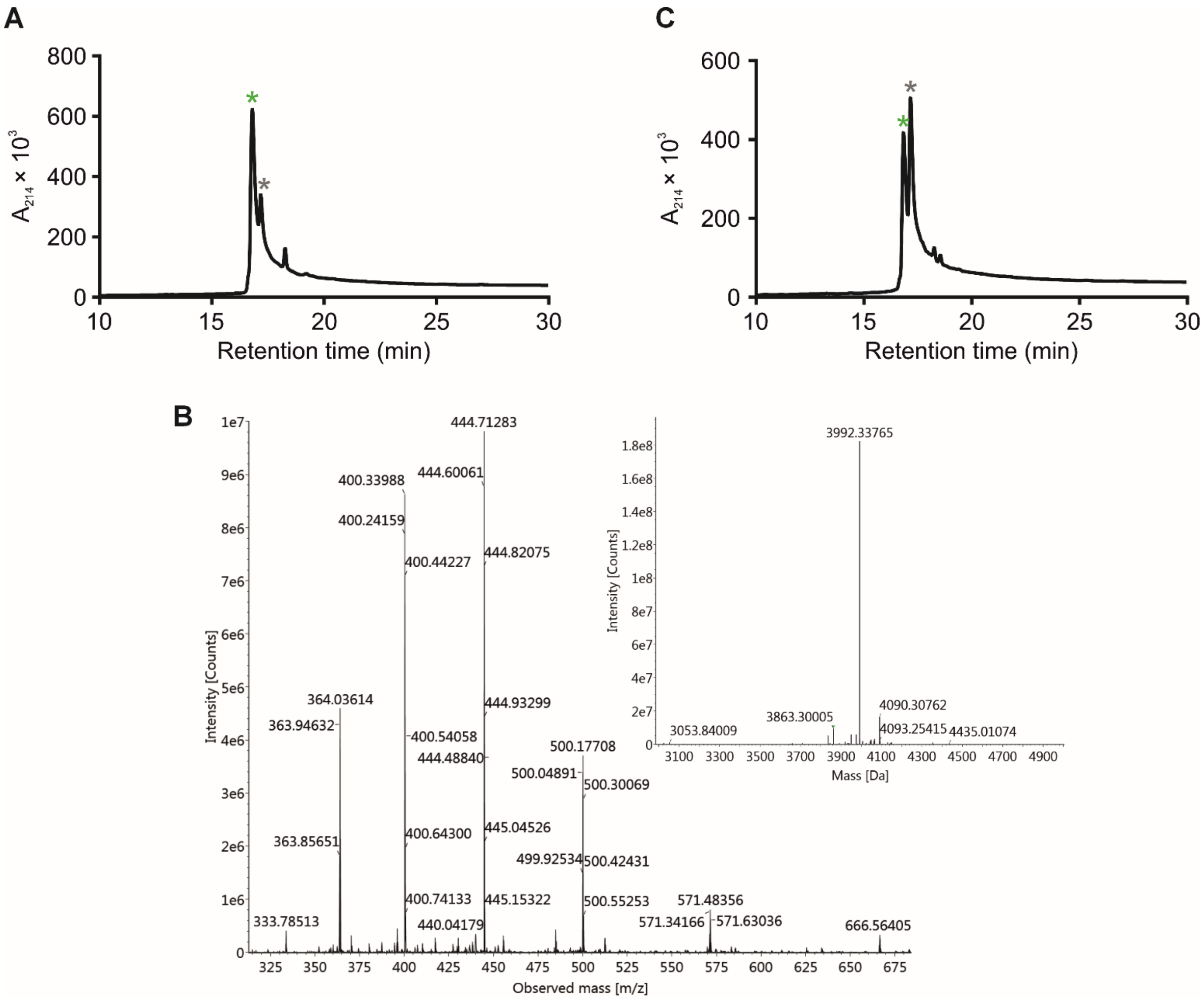

Figure S1: Analysis of linearly synthesised H4isoD24(1-37) peptide.

(A) Analytical RP-HPLC chromatogram and (B) HRMS spectrum of H4isoD24(1–37) acyl hydrazide peptide produced via linear synthesis. Inset shows deconvolution of mass spectrum. (C) Analytical RP-HPLC of a co-injected mixture of H4isoD24(1–37) and H4D24(1–37) acyl hydrazide peptides, both produced via linear synthesis. In both (A) and (C), green and grey asterisks denote peaks for the H4isoD24(1-37) and isomerised H4D24(1-37) acyl hydrazide peptides, respectively.

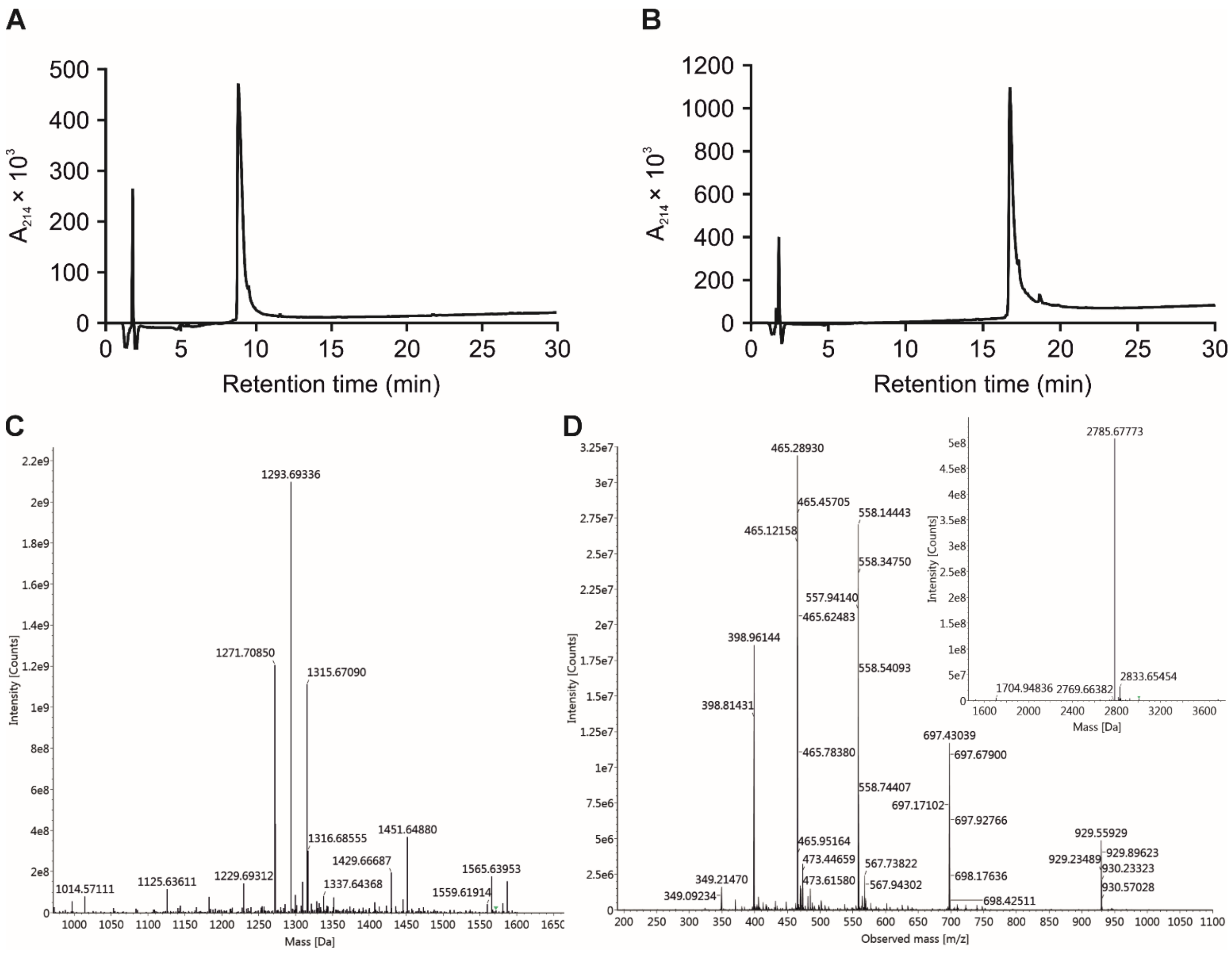

Figure S2: Analysis of H4isoD24(1-37) peptide building blocks.

Analytical RP-HPLC chromatograms of purified (A) H4(1–14) and (B) H4isoD24(A15C, 15–37) acyl hydrazide peptides. HRMS analyses of (C) H4(1–14) and (D) H4isoD24(A15C, 15–37) acyl hydrazide peptides. Inset in (D) depicts the deconvoluted spectrum.

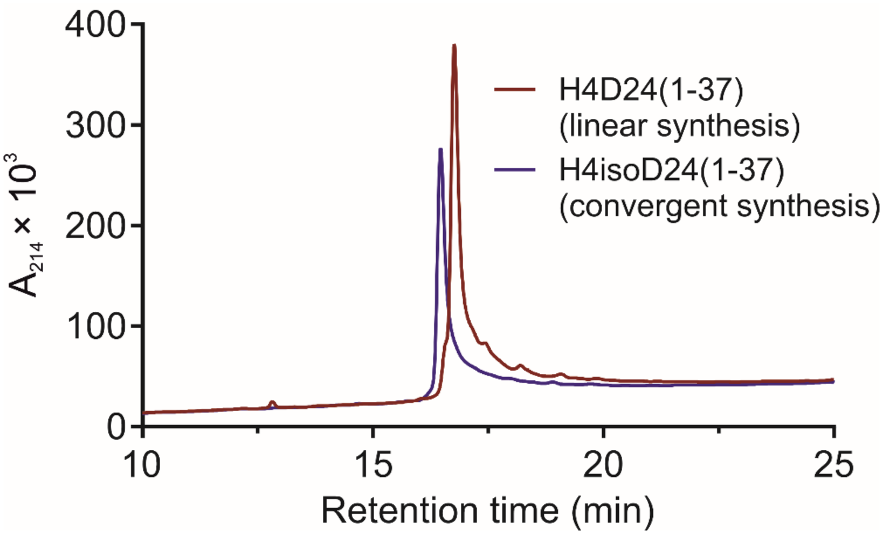

Figure S3: Comparison of H4D24(1-37) and H4isoD24(1-37) peptides.

Overlaid analytical RP-HPLC chromatograms of the C-terminal acyl hydrazide peptides H4D24(1–37), synthesised linearly (blue), and desulfurized H4isoD24(1–37), synthesised convergently (red).

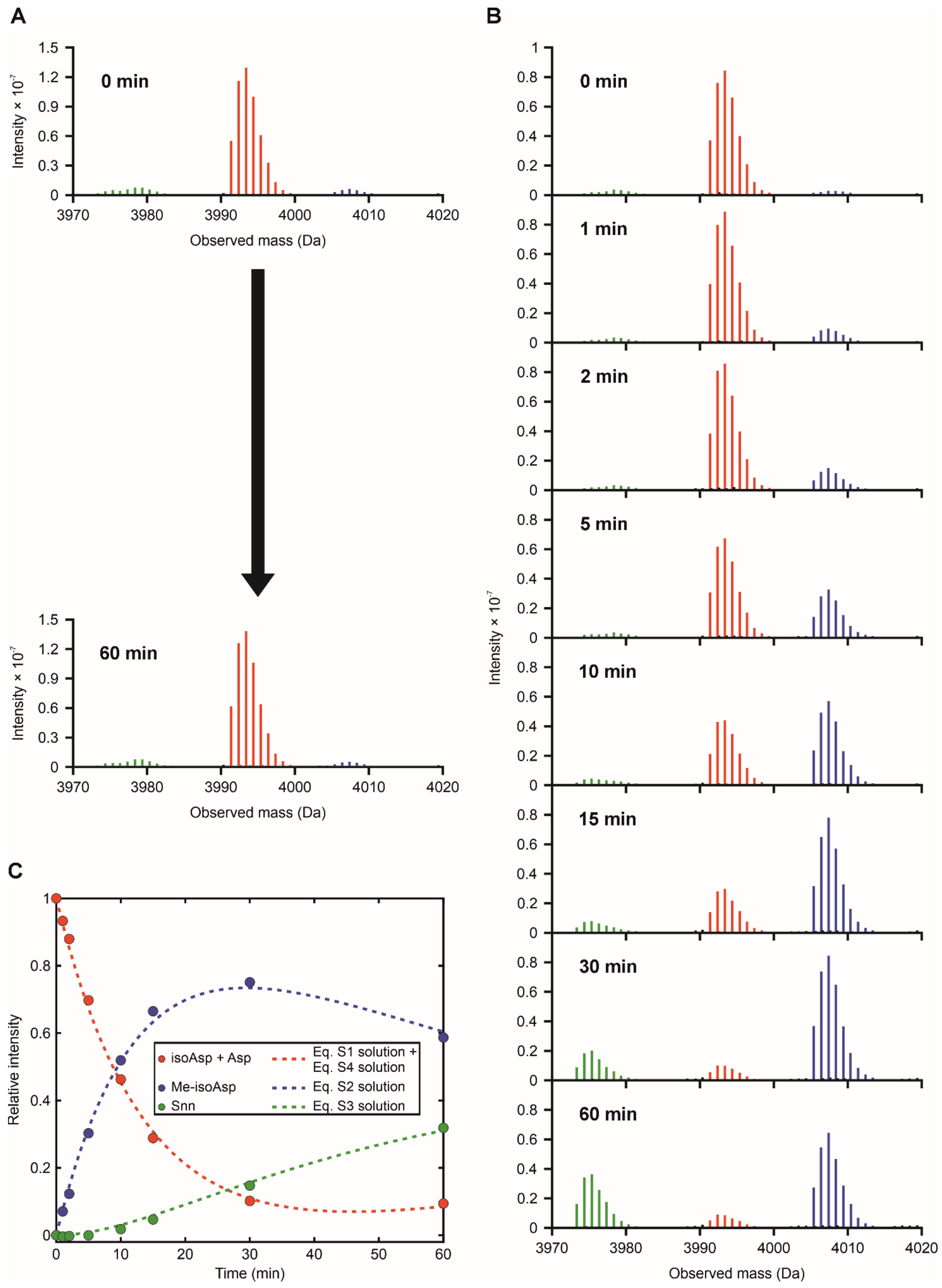

Figure S4: PIMT assays on H4 peptides.

PIMT-mediated methylation of (A) H4D24(1–37) and (B) H4isoD24(1–37) acyl hydrazide peptides. Green, red, and blue peaks represent the isotopic peaks of succinimidyl, unmethylated, and methylated peptidic species, respectively, with intensities greater than 104,000 after HRMS and deconvolution with centring. (C) Time course of reaction in (B), with each point given by the sum of the colour-matched peaks divided by the total peptide signal. Dashed lines represent non-linear, least-squares global fits performed using the solutions to Equations S1–S4.

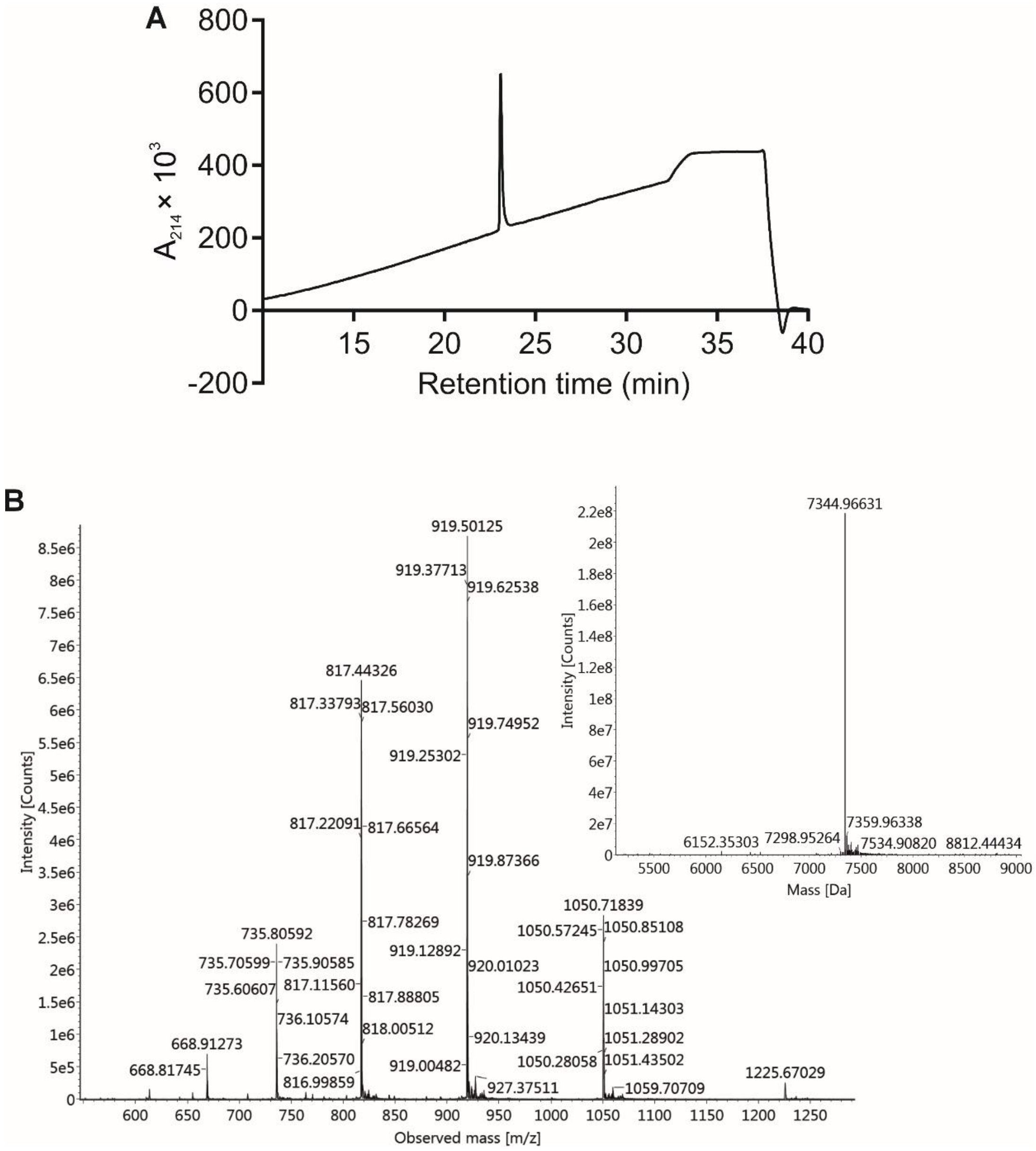

Figure S5: Analysis of H4(A38C, 38–102).

(A) Analytical RP-HPLC chromatogram and (B) HRMS spectrum of purified H4(A38C, 38–102). Inset depicts deconvoluted spectrum.

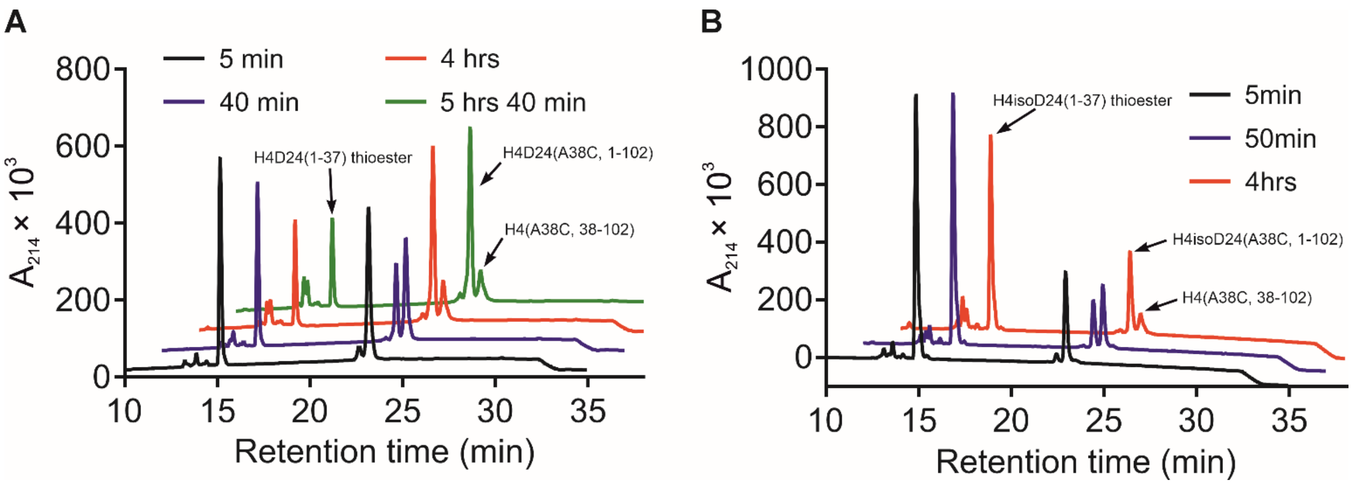

Figure S6. Synthesis of full-length H4 via native chemical ligation.

Native chemical ligation of N-terminal H4 peptides with the C-terminal truncation H4(A38C, 38–102). Reaction progress as measured by analytical RP-HPLC for the ligations with the (A) H4D24(1–37) and (B) H4isoD24(1–37) peptides. Retention times: r_t = 15.2 min [H4D24(1–37) thioester], r_t = 14.9 min [H4isoD24(1–37) thioester], r_t = 23.2 min and 22.9 min [H4(A38C, 38–102) in (A) and (B), respectively], r_t = 22.6 min [H4D24(A38C, 1–102)], and r_t = 22.4 min [H4isoD24(A38C, 1–102)].

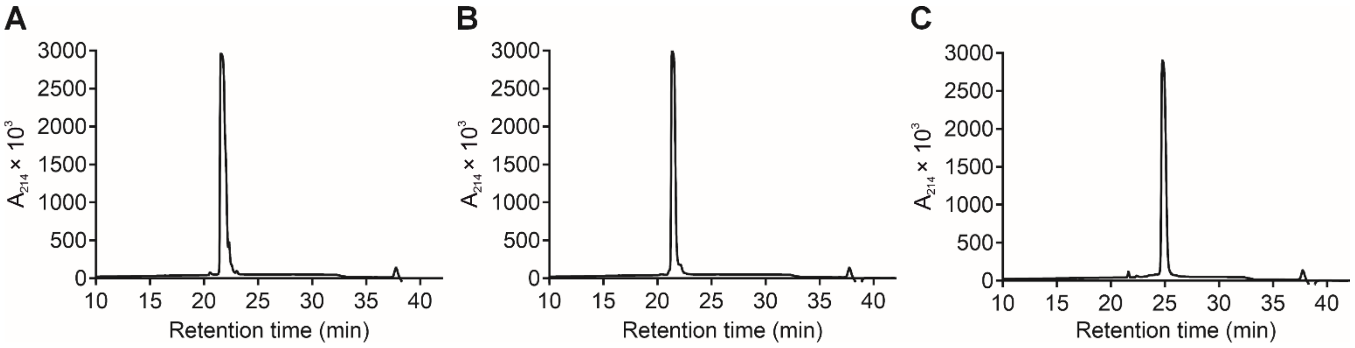

Figure S7. HPLC analysis of purified recombiant histones.

Analytical RP-HPLC chromatograms of purified recombinantly-produced histones (A) H2A, (B) H2B, and (C) H3.

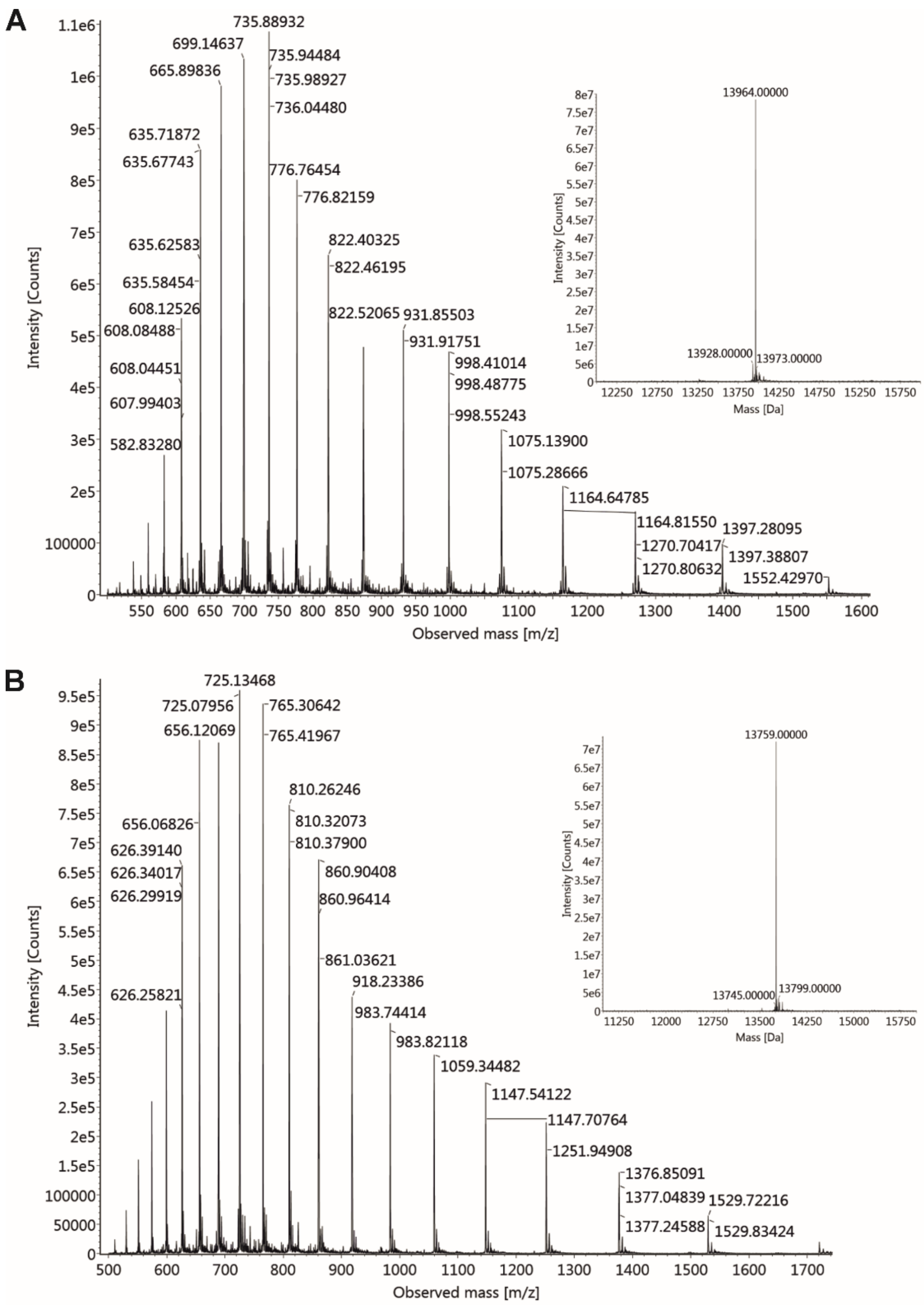

A
735.88932
699.14637
735.94484
665.89836
735.98927
736.04480
635.71872
635.67743
776.76454
776.82159
822.40325
635.62583
822.46195
635.58454
608.12526
822.52065
931.85503
608.08488
931.91751
998.41014
998.48775
608.04451
607.99403
998.55243
582.83280
1075.13900
1075.28666
1164.64785
1164.81550
1397.28095
1270.70417
1397.38807
1270.80632
1552.42970
Intensity [Counts]
Observed mass [m/z]
13964.00000
13928.00000
13973.00000
Mass [Da]
B
725.13468
725.07956
765.30642
656.12069
765.41967
810.26246
656.06826
810.32073
626.39140
810.37900
626.34017
860.90408
626.29919
860.96414
861.03621
918.23386
626.25821
983.74414
983.82118
1059.34482
1147.54122
1147.70764
1251.94908
1376.85091
1377.04839
1529.72216
1377.24588
1529.83424
Intensity [Counts]
Observed mass [m/z]
13759.00000
13745.00000
13799.00000
Mass [Da]

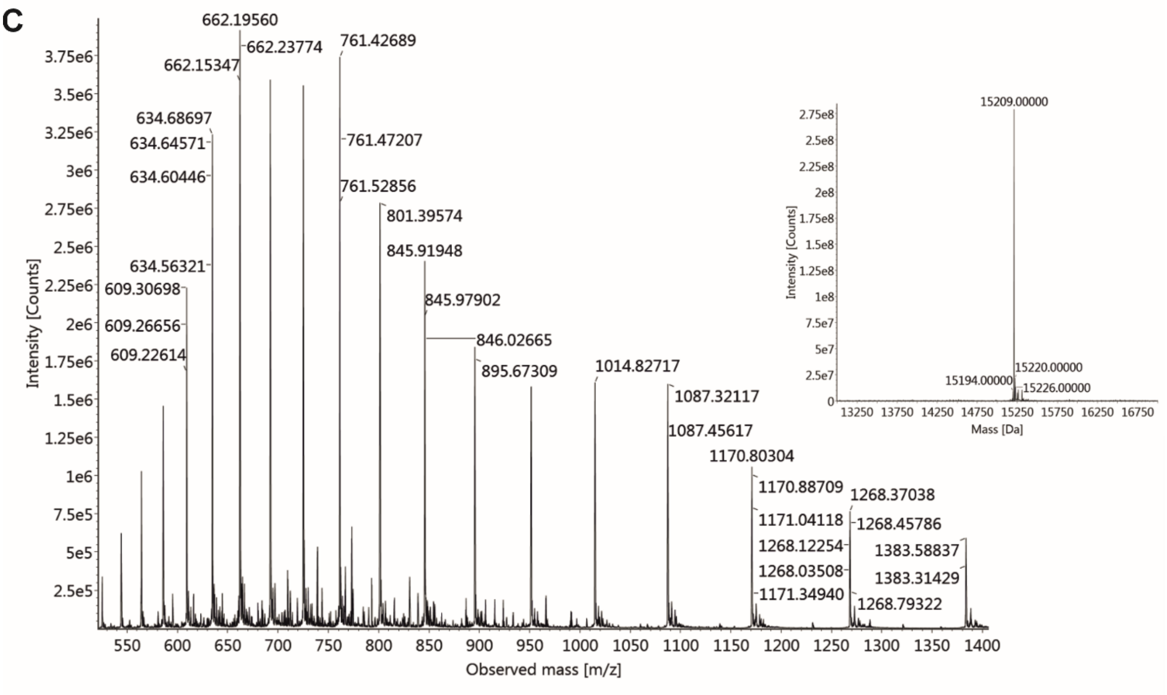

Figure S8: HRMS analysis of purified recombinantly-produced histones.

HRMS spectra of purified recombinantly-produced histones (A) H2A, (B) H2B, and (C) H3. Insets depict deconvoluted spectra.

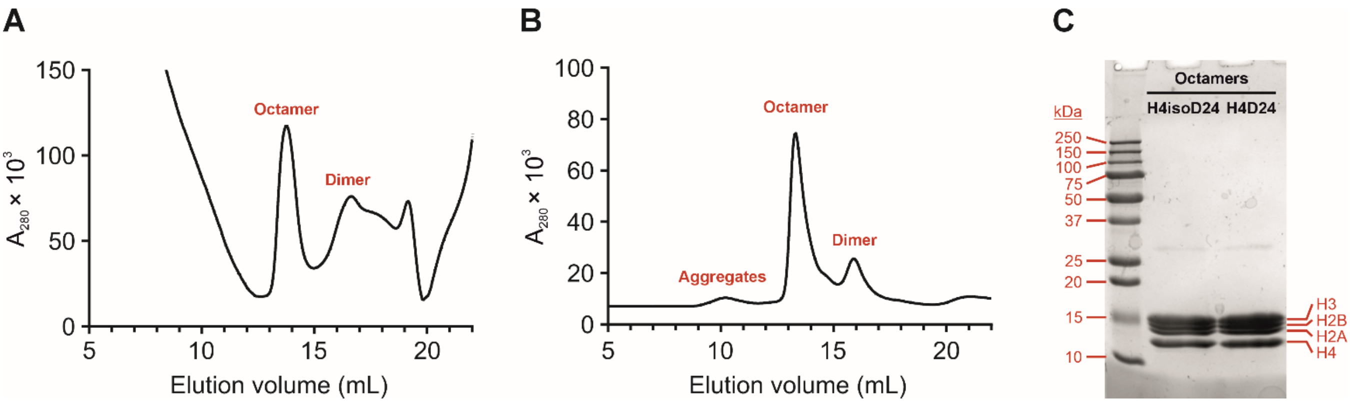

Figure S9: Purification and analysis of histone octamers.

SEC of histone mixtures after octamer refolding, incorporating either (A) H4D24 (elution volume = 13.8 mL) or (B) H4isoD24 (elution volume = 13.3 mL). (C) SDS-PAGE of SEC-purified octamer fractions.

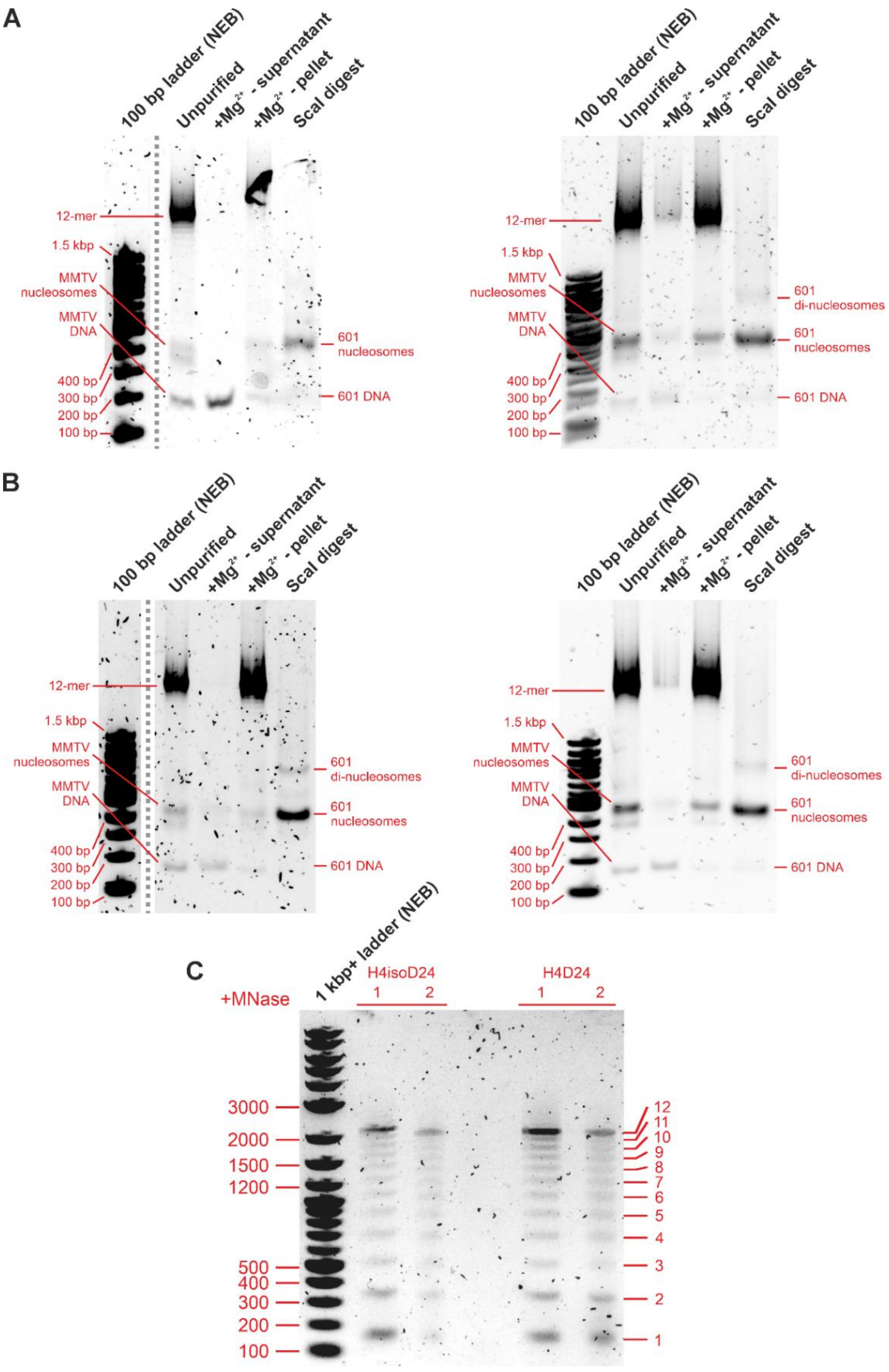

Figure S10: Analyses of H4(iso)D24-containing 12-mers.

Native APAGE of two independent preparations of 12-mers containing (A) H4D24 or (B) H4isoD24. Gel images show unpurified mixtures prior to purification by Mg^{2+}-mediated self-association ("Unpurified"), pure pellet fractions ("+Mg^{2+} - pellet") , the species that were successfully removed in the supernatant during purification ("+Mg^{2+} - supernatant"), and the purified 12-mers after site-specific digestion by ScaI ("ScaI digest"). Replicates shown in (A) and (B) were also used for Mg^{2+}-mediated self-association assays (Figure 3). (C) Agarose gel electrophoresis after digestion by MNase of two independent preparations of 12-mers containing each H4 variant.

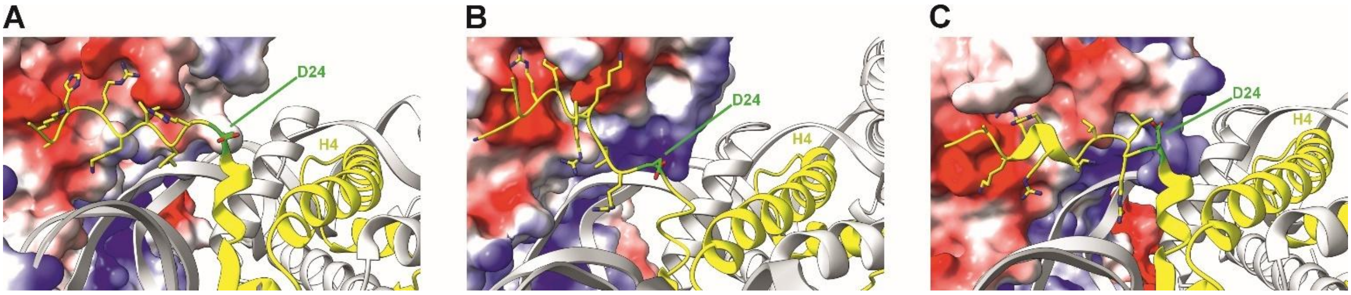

Figure S11. Interactions between the H4 N-terminal tail and selected nucleosome remodellers.

ChimeraX renderings of *S. cerevisiae* orthologs of the nucleosome remodellers (A) Chd1 (PDB: 5O9G)[9], (B) Snf2 (PDB: 5X0Y)[10], and (C) ISW1 (PDB: 6K1P)[11] are shown in complex with H4 (yellow); D24 is highlighted in green.

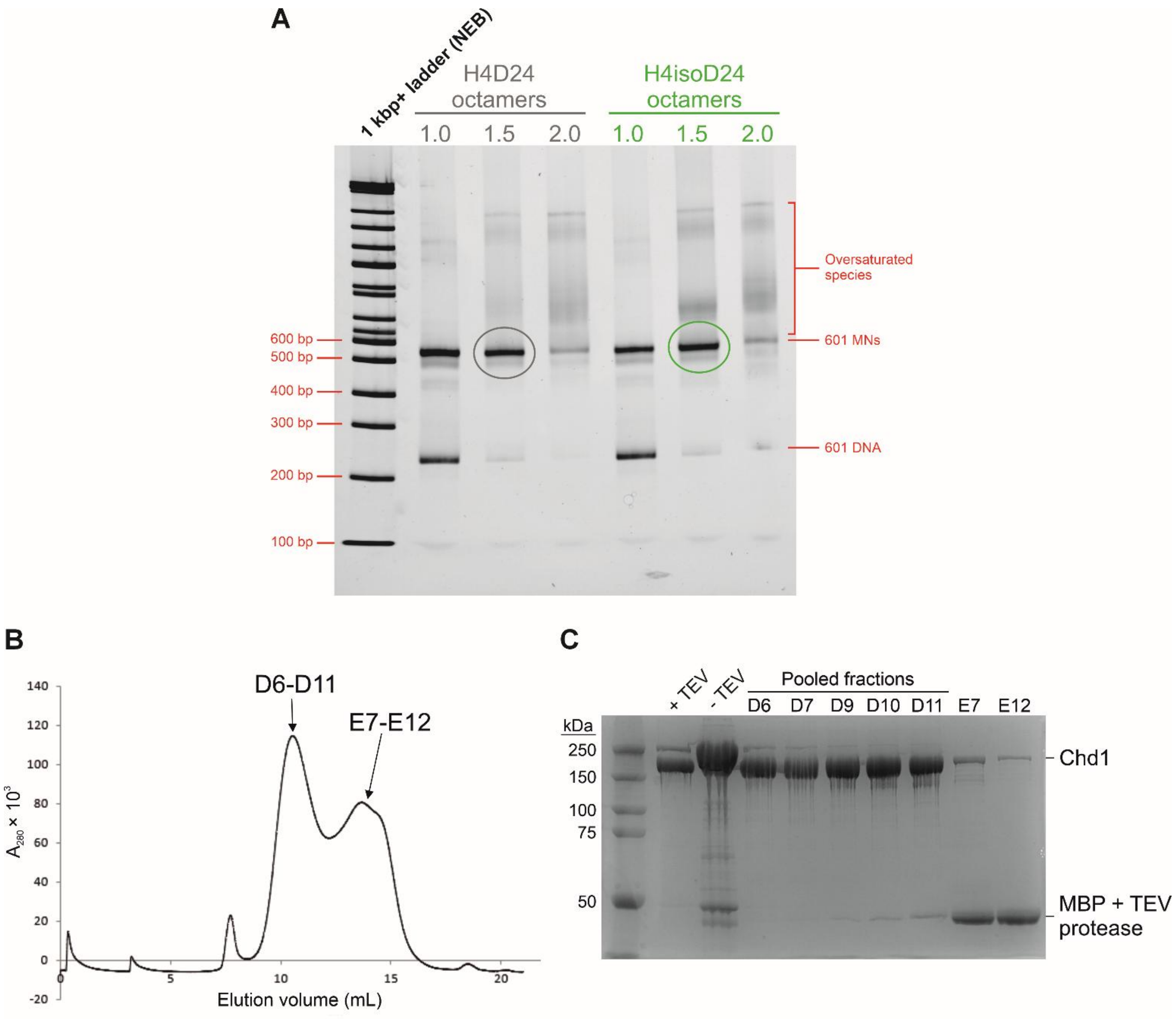

Figure S12. MN assembly and Chd1 purification

(A) Native PAGE of an example MN assembly with H4D24- or H4isoD24-containing octamers and octamer:DNA ratios of 1, 1.5, or 2. Circles indicate the desired MNs: MNs assembled with ratios of 1.5 were chosen due to the absence of both free “601” DNA and oversaturated species. (B) SEC of Chd1 after cleavage of the MBP solubility tag by TEV protease, with fractions collected at the two major peaks. (C) SDS-PAGE analysis of TEV protease digestion and SEC in (B), indicated the pooled, purified fractions used for subsequent remodelling assays.

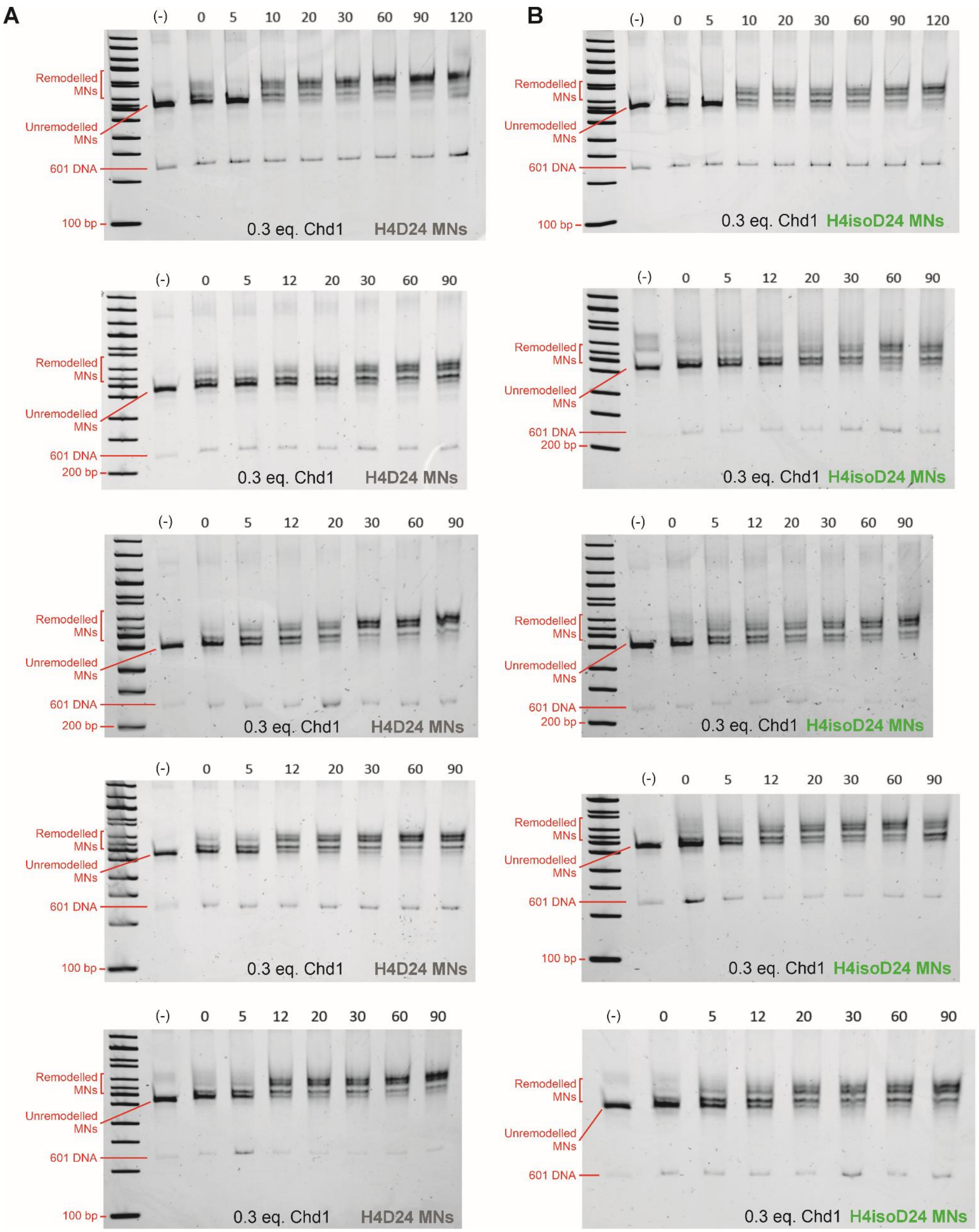

Figure S13. Native PAGE of remodelling assays

Native PAGE of Chd1 remodelling assays on (A) H4D24- and (B) H4isoD24-containing MNs. Five biological replicates were performed with 0.3 eq. of Chd1 to MNs, though the 5-minute timepoints of the top panels of (A) and (B) were excluded from subsequent analyses as clear outliers. (-) indicates no Chd1,

numbers above lanes indicate the reaction duration in minutes before quenching, and the DNA ladder was 1 kbp+ ladder (NEB).

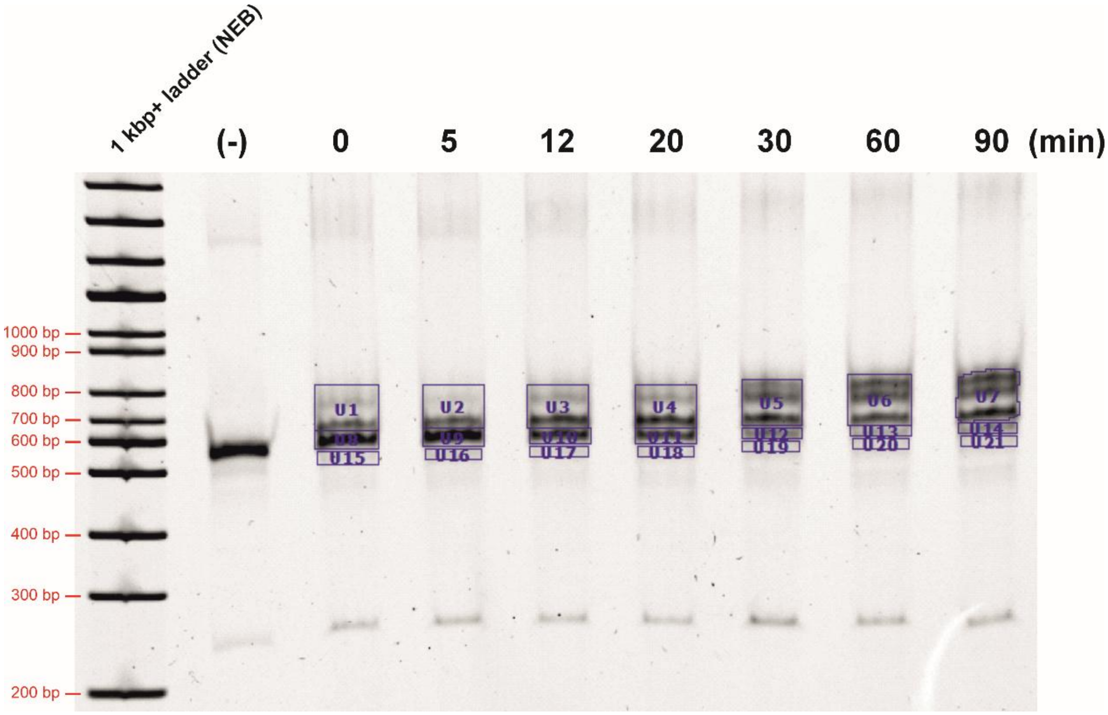

Figure S14: Example analysis of Chd1 remodelling assays on MNs

The remodelled fraction of H4D24-containing MNs at each given timepoint was calculated by dividing the intensity of remodelled MNs (all upper bands) to the summed intensities of remodelled and unaffected bands, with background subtraction. For example, U1–7 = remodelled MNs; U8–14 = unaffected MNs; U15–21 = background. Thus, for the 0 min timepoint the remodelled fraction would be (U1-U15)/[(U1-U15) + (U8-U15)].

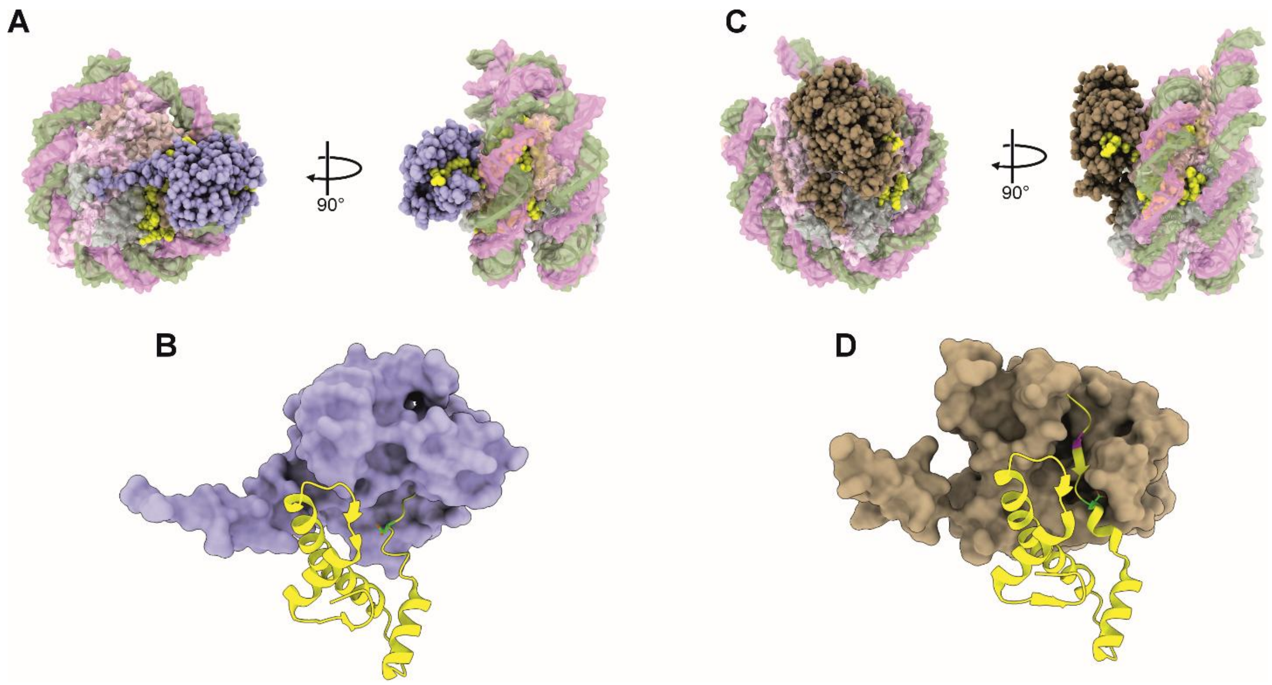

Figure S15. Set8 and Suv4-20h1 interactions with the nucleosome and/or H4.

(A) Structure of Set8 (purple) and a nucleosome with 145 bp of the “601” positioning sequence (H4 in yellow), as determined by cryo-EM (PDB: 7D1Z[12]). (C) Structure of Suv4-20h1 (brown) and a nucleosome with 167 bp of the “601” positioning sequence (H4 in yellow), obtained using cryo-EM (PDB: 8JHG).[13] (B, D) Same structures as in (A) and (C), respectively, with all nucleosomal species except H4 [H4, yellow; H4D24, green; H4K20 (H4K20M in the Suv4-20H1 structure), magenta] removed.

(A) and (C) reveal similar modes of macromolecular engagement for the two methyltransferases, with anchoring to the H2A/H2B acidic patch and interaction with an outward conformation of the H4 N-terminal tail on the same face. However, binding to the latter domain differs significantly between the two enzymes. The extended catalytic cleft of Suv4-20h1 features an overhang—missing in Set8—that makes extensive contacts with the residues C-terminal to H4K20 [cf. (B) and (D)]. Indeed, recognition of the H4 N-terminal tail by Set8 is primarily mediated by hydrogen bonding with residues inclusive of H4R17 and H4V21, while in Suv4-20h1 the bounds consist of a putative cation-π interaction with H4R19 and hydrogen bonds directly to H4D24 (Figure 5A). These shifted binding interfaces suggest that Suv4-20h1 may be highly susceptible to the presence of H4isoD24 and Set8 relatively more tolerant, hypotheses supported by *in vitro* binding to libraries generated by SPOT peptide synthesis with the canonical amino acids[14] or alanine scanning,[15] respectively.

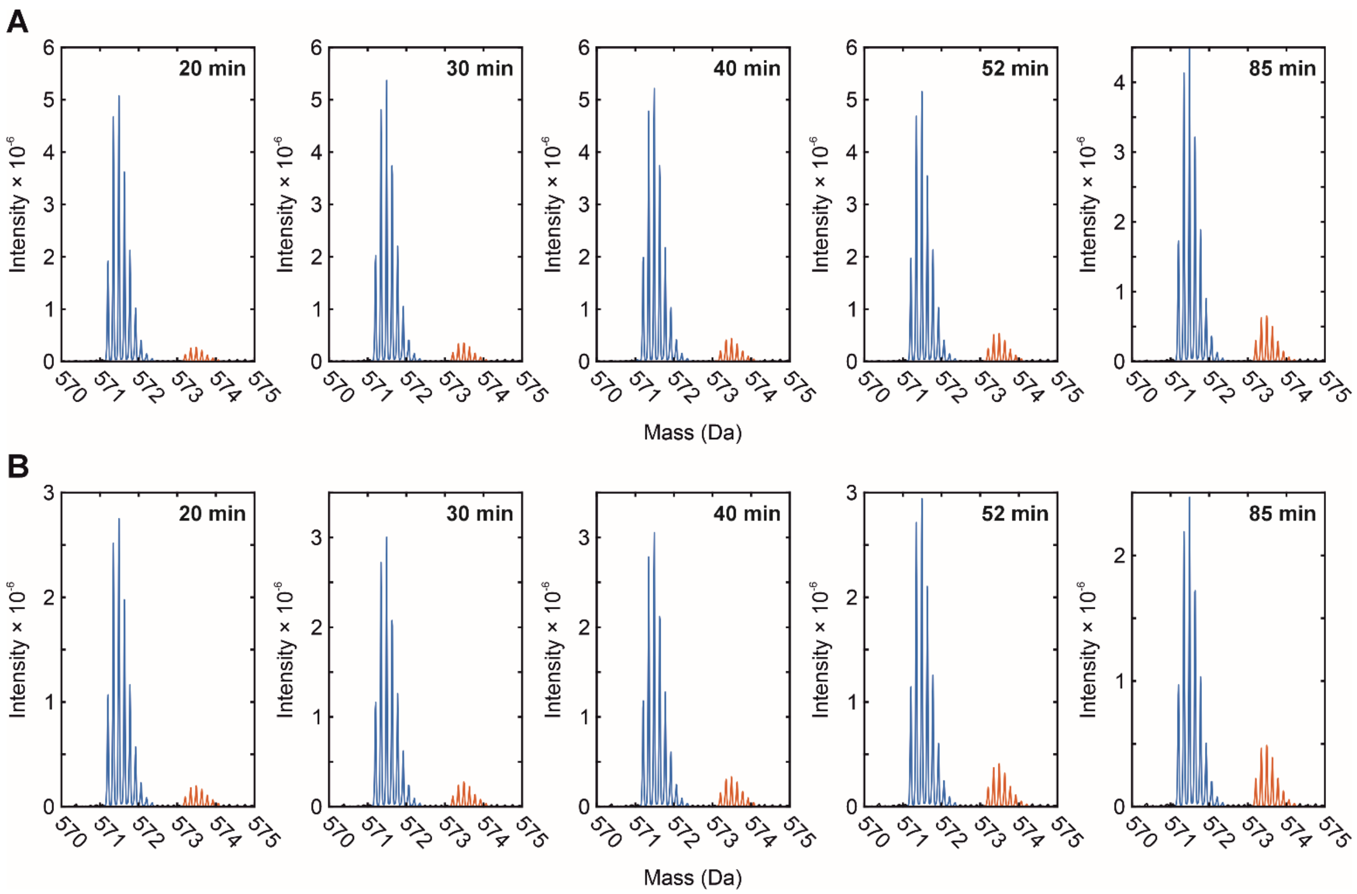

Figure S16. Set8 monomethylation of H4(iso)D24(1–37) peptides, example HRMS time course.

Mass spectra used to quantify Set8 monomethylation of H4K20 on (A) H4D24(1–37) and (B) H4isoD24(1–37) acyl hydrazide peptides at concentrations of 8 µM (Figure S17D). Clusters represent the isotopic distributions of the 7+ charge states for unmethylated (blue) and monomethylated (red) species. The intensities of the highest peaks in each cluster were used to calculate the relative amounts of each species.

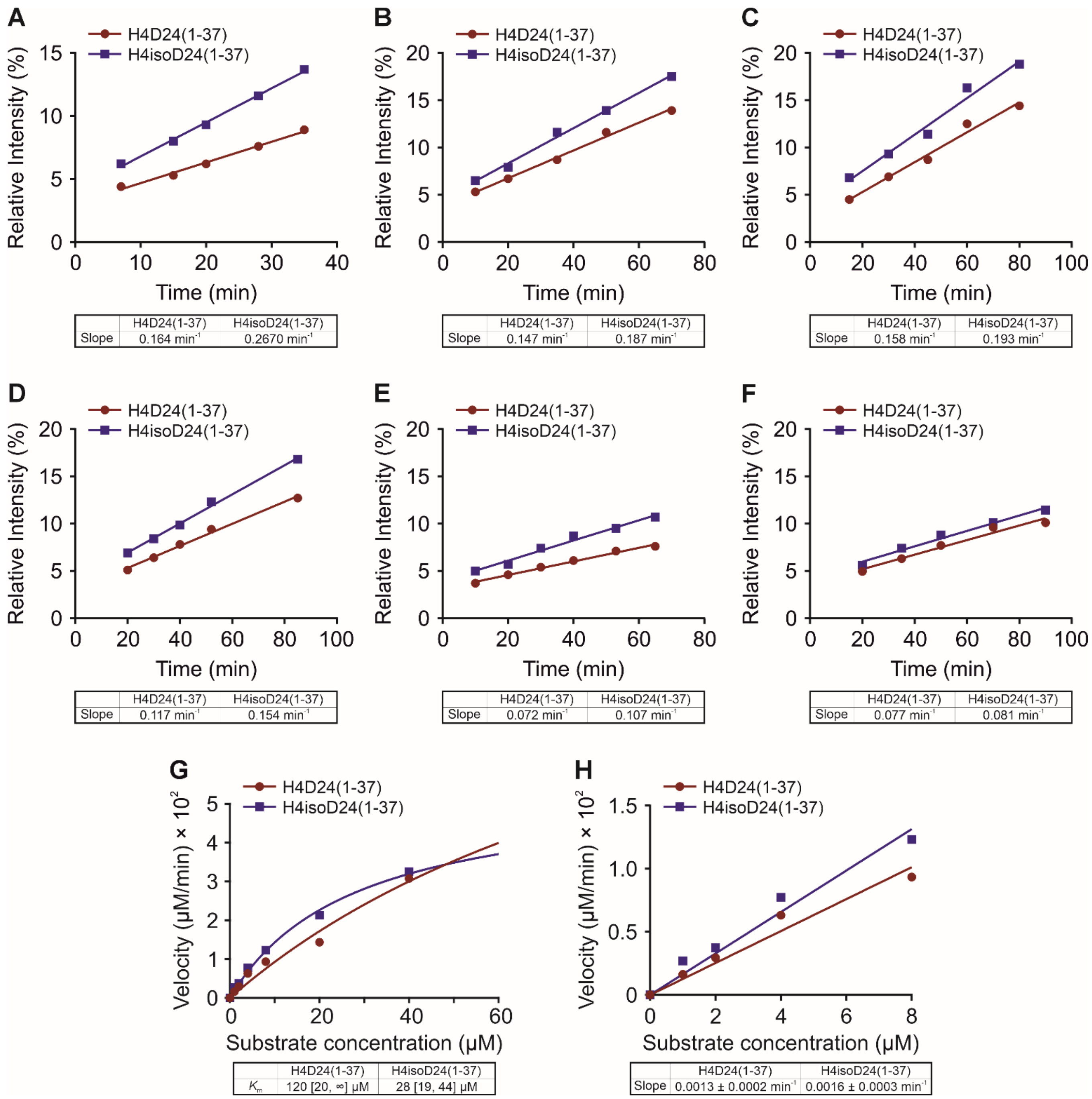

Figure S17. Set8 monomethylation of H4(iso)D24(1–37) peptides, analyses.

Relative intensities of the H4K20me1 MS signal vs. time (min) after Set8 monomethylation of H4D24(1–37) (red circles) and H4isoD24(1–37) (blue squares) acyl hydrazide peptides at concentrations of (A) 1 µM, (B) 2 µM, (C) 4 µM, (D) 8 µM, (E) 20 µM, and (F) 40 µM. Lines indicate linear regressions of the data with fitted slopes given below each graph. (G) Michaelis-Menten reaction velocities [(µM/min) × 10^2] vs. substrate concentration (µM) using the slopes from (A)–(F), with the same colour-coding. Lines represent fits of the corresponding data to the Michaelis-Menten equation, and resulting K_m values with asymmetric confidence intervals are shown below the graph. (H) As in (G), but using the slopes from only (A)–(D). Lines represent linear regressions of the corresponding data, and the resulting slopes (shown below the graph) were used to approximate k_{cat}/K_m as described in the main text.

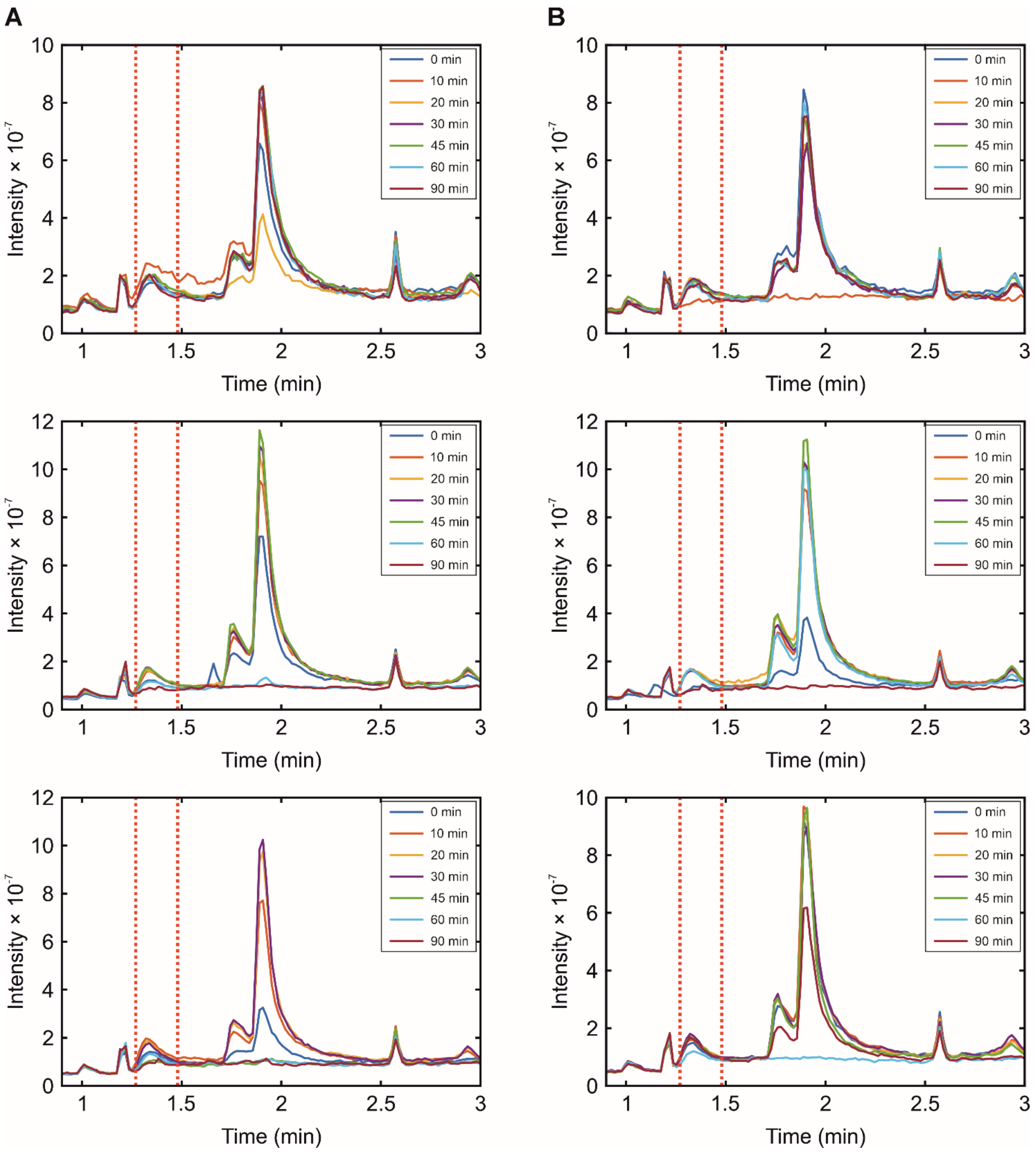

Figure S18: TICs of H4 peptide methyltransferase assays.

TICs obtained for every timepoint of all three replicates of Set8/Suv4-20h1 methylation reactions on (A) H4D24(1–37) and (B) H4isoD24(1–37) acyl hydrazide peptides. Red, dashed lines denote the boundaries of the region from which mass spectra were combined via summation for further analysis.

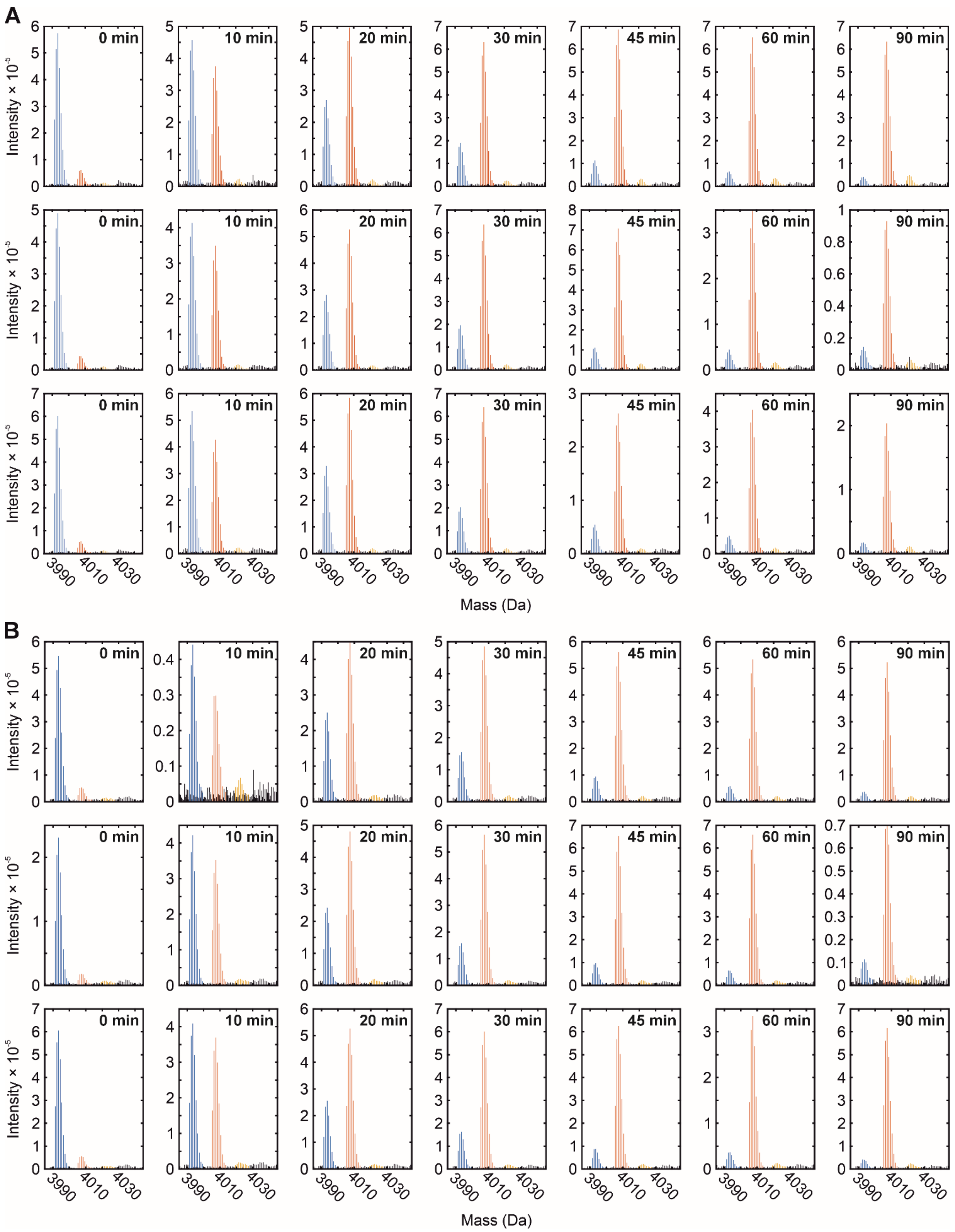

Figure S19: Mass spectra of H4 peptide methyltransferase assays.

Deconvoluted and centred mass spectra obtained from the TICs in Figure S19 for every timepoint of all three replicates of Set8/Suv4-20h1 methylation reactions on (A) H4D24(1–37) and (B) H4isoD24(1–37) acyl hydrazide peptides. The H4K20me0, H4K20me1, and H4K20me2 signals summed together for relative intensity calculations are shown in blue, red, and yellow, respectively.

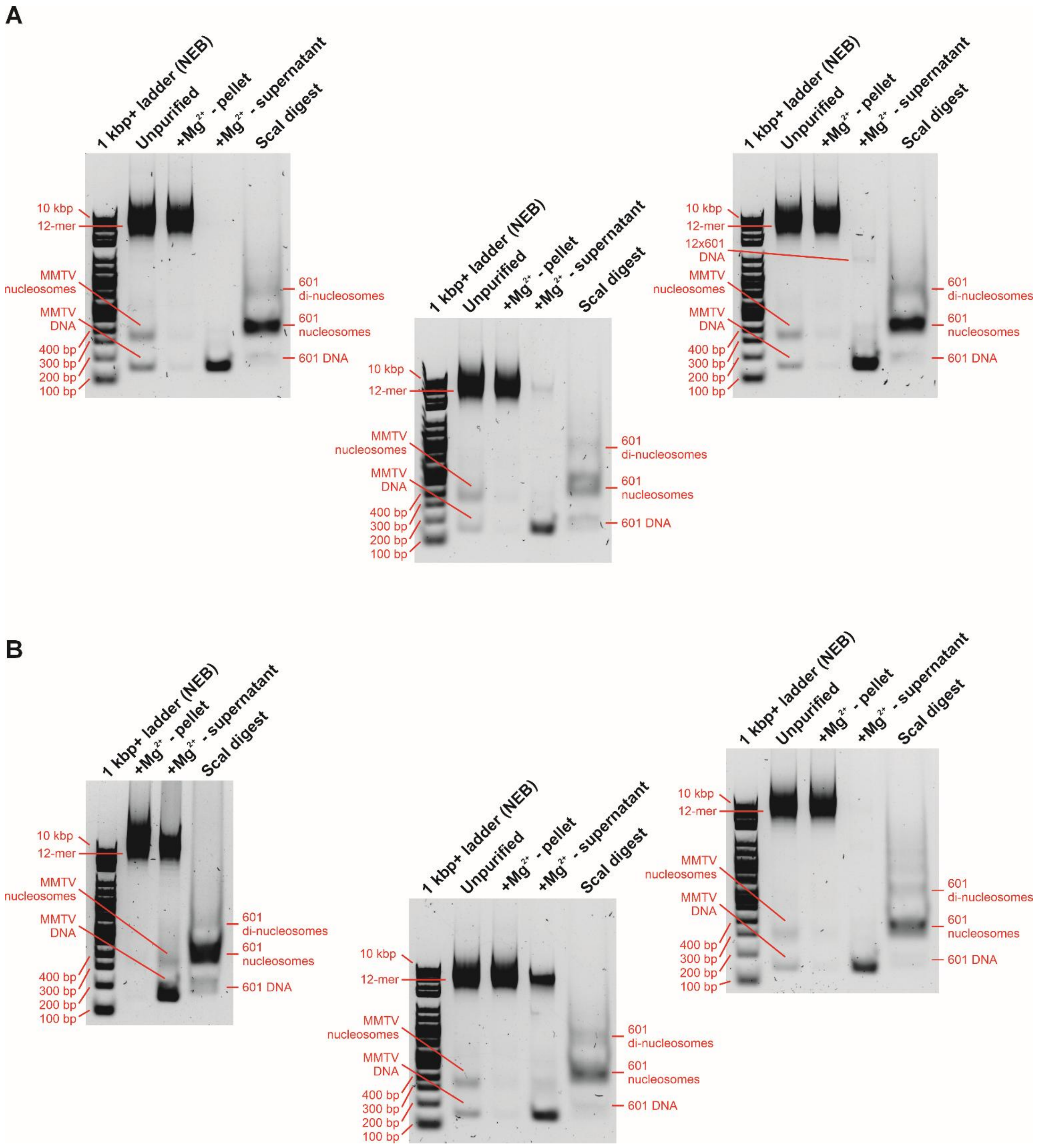

Figure S20: Analysis of ‘designer’ nucleosome arrays used for dimethylation assays.

Native APAGE of all three independent replicates of (A) H4D24- and (B) H4isoD24-containing 12-mers used for Set8/Suv4-20h1 methylation assays. Gel images show unpurified mixtures prior to purification by Mg^{2+}-mediated self-association (“Unpurified”), pure pellet fractions used for assays (“+Mg^{2+} - pellet”) , the species that were successfully removed in the supernatant during purification (“+Mg^{2+} - supernatant”), and the purified 12-mers after site-specific digestion by ScaI (“ScaI digest”).

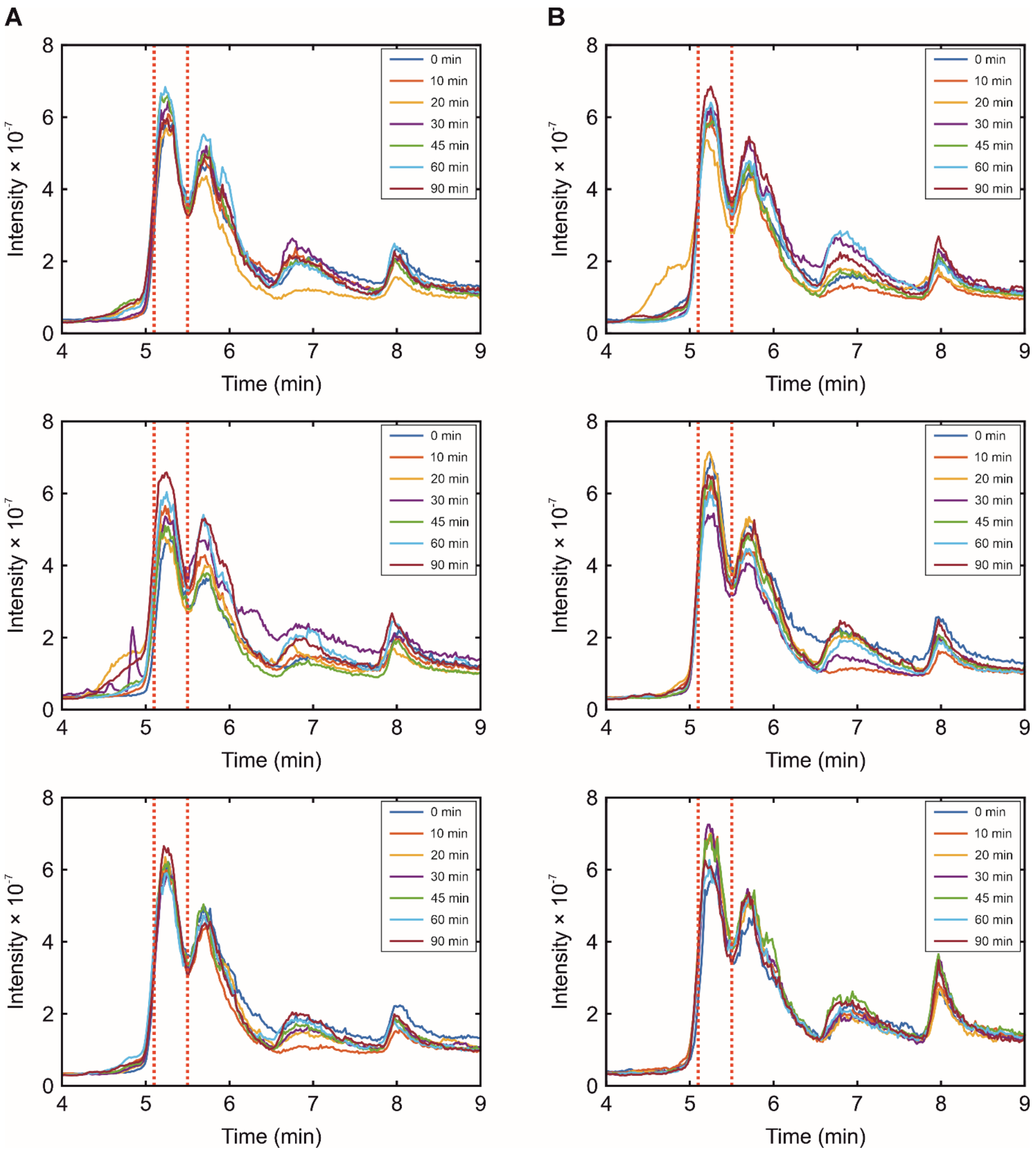

Figure S21: TICs of nucleosome array dimethylation assays.

TICs for every timepoint of all three replicates of Set8/Suv4-20h1 methylation reactions on (A) H4D24- and (B) H4isoD24-containing 12-mers. Red, dashed lines denote the boundaries of the region from which mass spectra were combined via summation for further analysis.

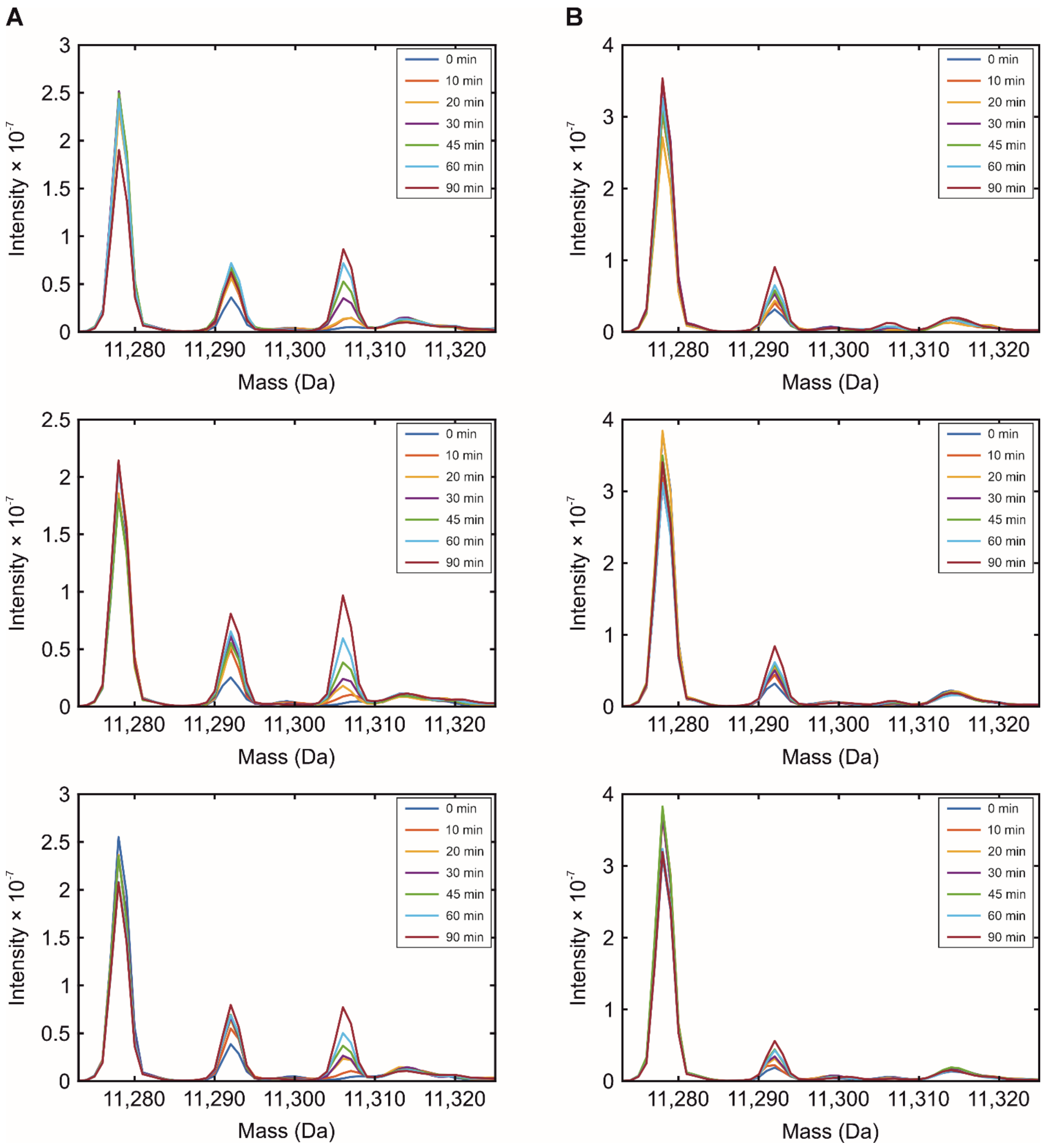

Figure S22. Deconvoluted mass spectra of nucleosome array dimethylation assays.

Deconvoluted mass spectra obtained from the TICs in Figure S21 for every timepoint of all three replicates of Set8/Suv4-20h1 methylation reactions on (A) H4D24- and (B) H4isoD24-containing 12-mers.

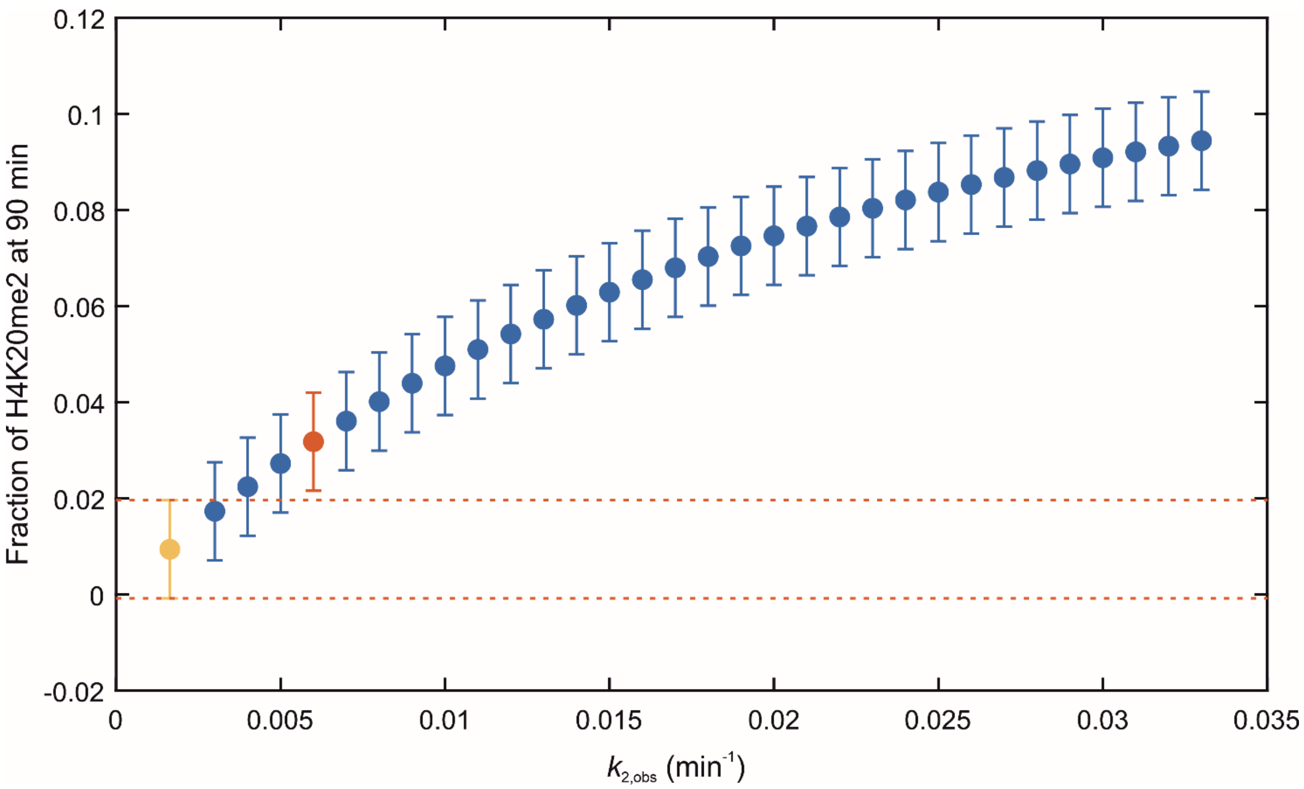

Figure S23. Limit-of-detection analysis for the quantification of $k_{2,obs}$

Fraction of H4K20me2 formed after 90 min as a function of the dimethylation rate, $k_{2,obs}$, calculated using Equation S10. $k_{1,obs}$ is held fixed at the experimentally observed value for H4isoD24-containing 12-mers, and error bars represent a 95% CI calculated from the experimental precisions in Table 1 of $k_{1,obs}$ and $k_{2,obs}$ for the same substrate (using the built-in MATLAB function “nlpredci”). The fractions of H4K20me2 at the experimentally observed $k_{2,obs}$ = 0.002 min^{-1} and at the first statistically significant point with $k_{2,obs}$ = 0.006 min^{-1} are highlighted in yellow and red, respectively. Red, dashed lines represent the 95% CI of the experimental point.

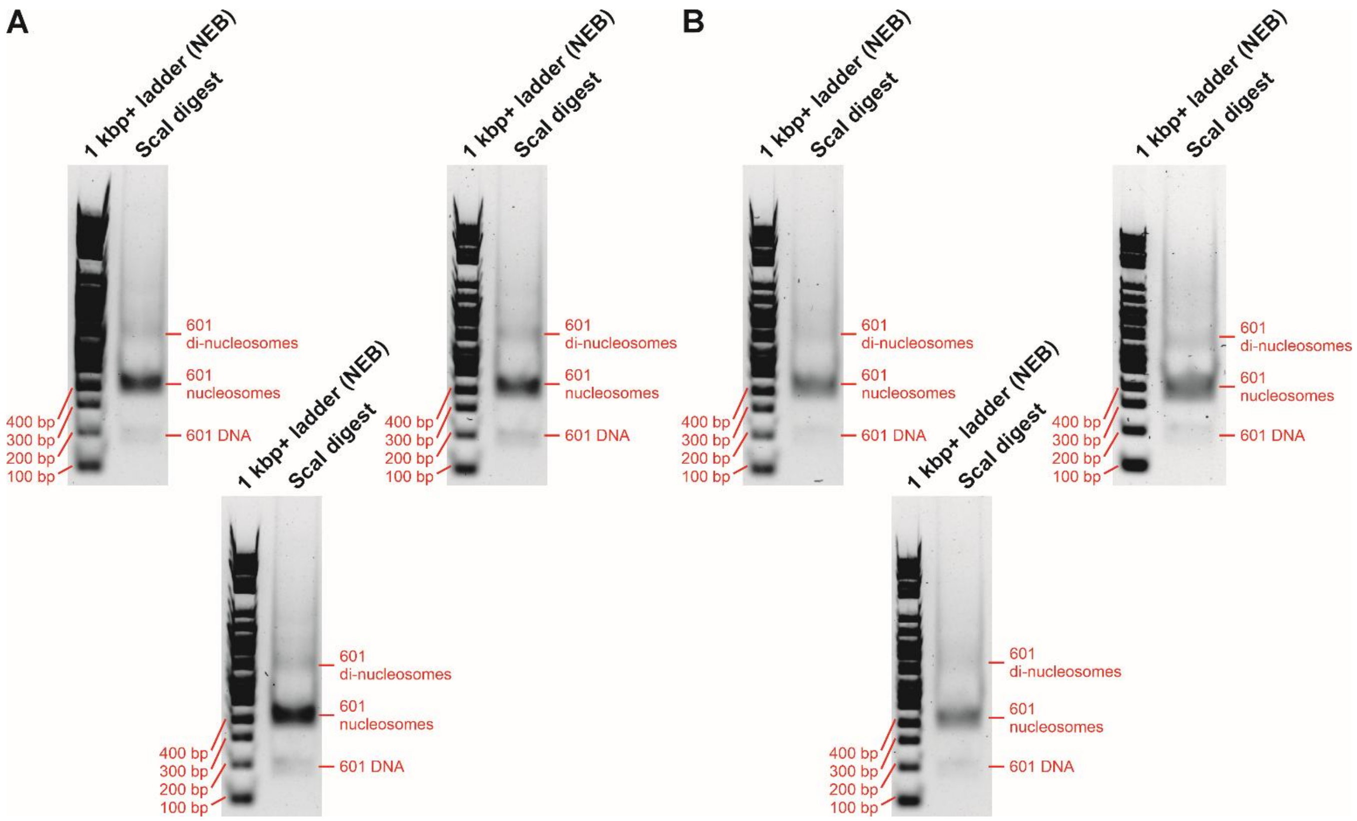

Figure S24. Analysis of nucleosome array integrity after dimethylation assays.

Native APAGE of all three replicates of (A) H4D24- and (B) H4isoD24-containing 12-mers subjected to ScaI restriction digest after Set8/Suv4-20h1 methylation assays.

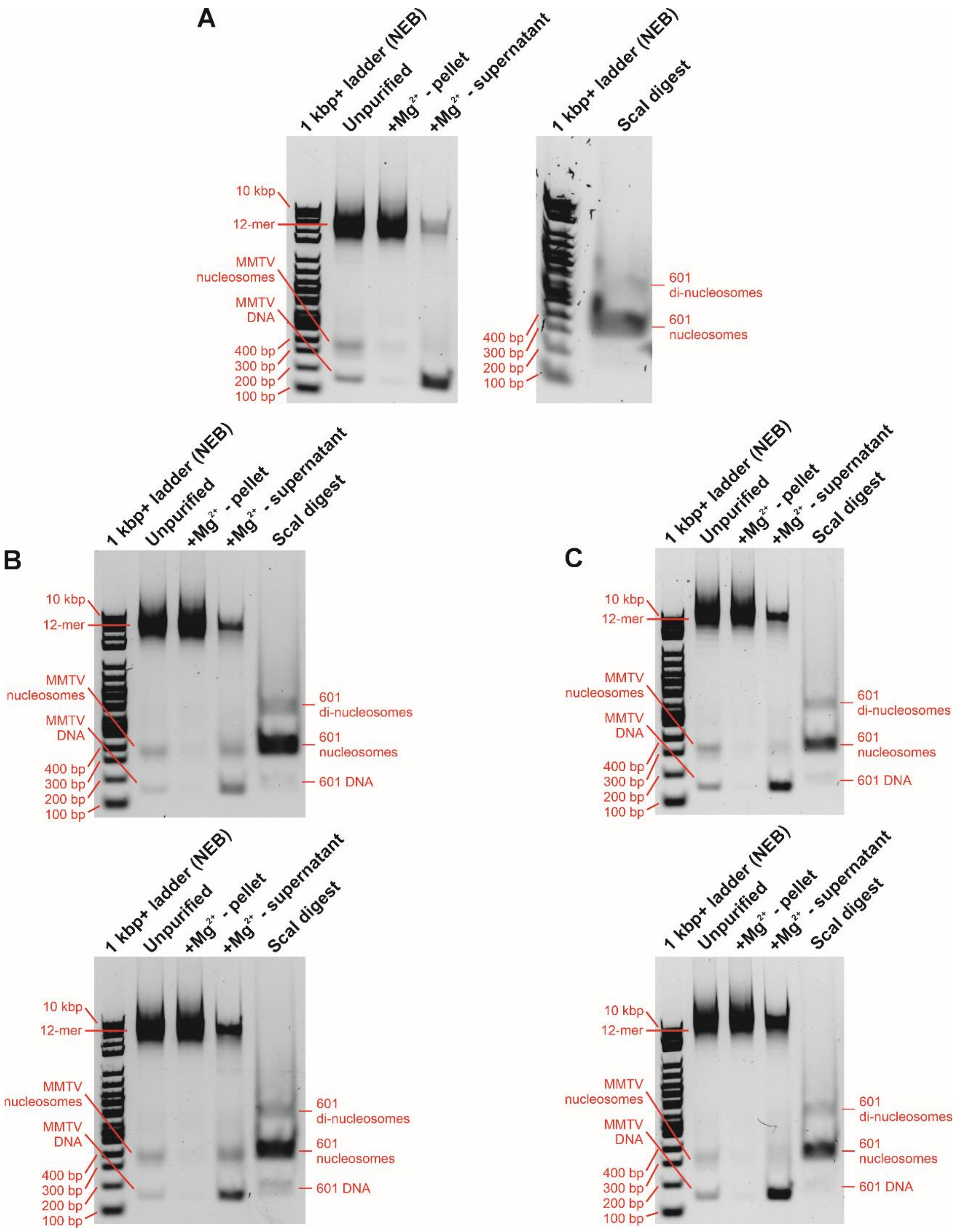

Figure S25. Analysis of 'designer' nucleosome arrays used for monomethylation assays.

Native APAGE of all independent 12-mer constructs used for Set8 monomethylation assays, assembled using (A) only H4D24-containing octamers or ratios of (B) 2:1 and (C) 1:1 H4D24- and H4isoD24-containing octamers, respectively. Gel images show unpurified mixtures prior to purification by Mg^{2+}-mediated self-association ("Unpurified"), pure pellet fractions used for assays ("+Mg^{2+} - pellet") , the species that were successfully removed in the supernatant during purification ("+Mg^{2+} - supernatant"), and the purified 12-mers after site-specific digestion by ScaI ("ScaI digest").

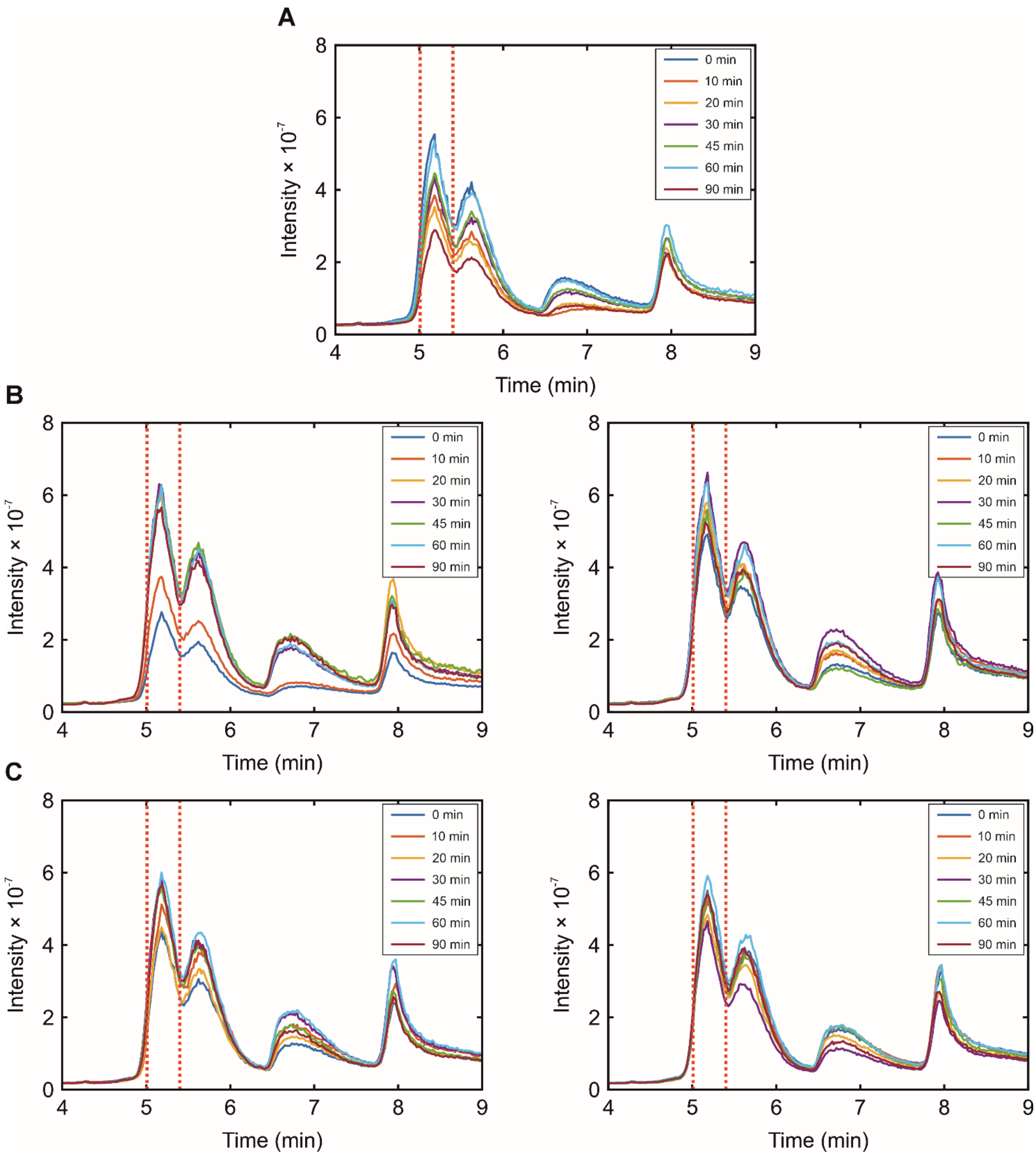

Figure S26. TICs of nucleosome array monomethylation assays.

TICs for every timepoint of all replicates of Set8 monomethylation reactions on 12-mers containing (A) only H4D24-containing octamers or (B) 2:1 and (C) 1:1 ratios of H4D24- and H4isoD24-containing octamers, respectively. Red, dashed lines denote the boundaries of the region from which mass spectra were combined via summation for further analysis.

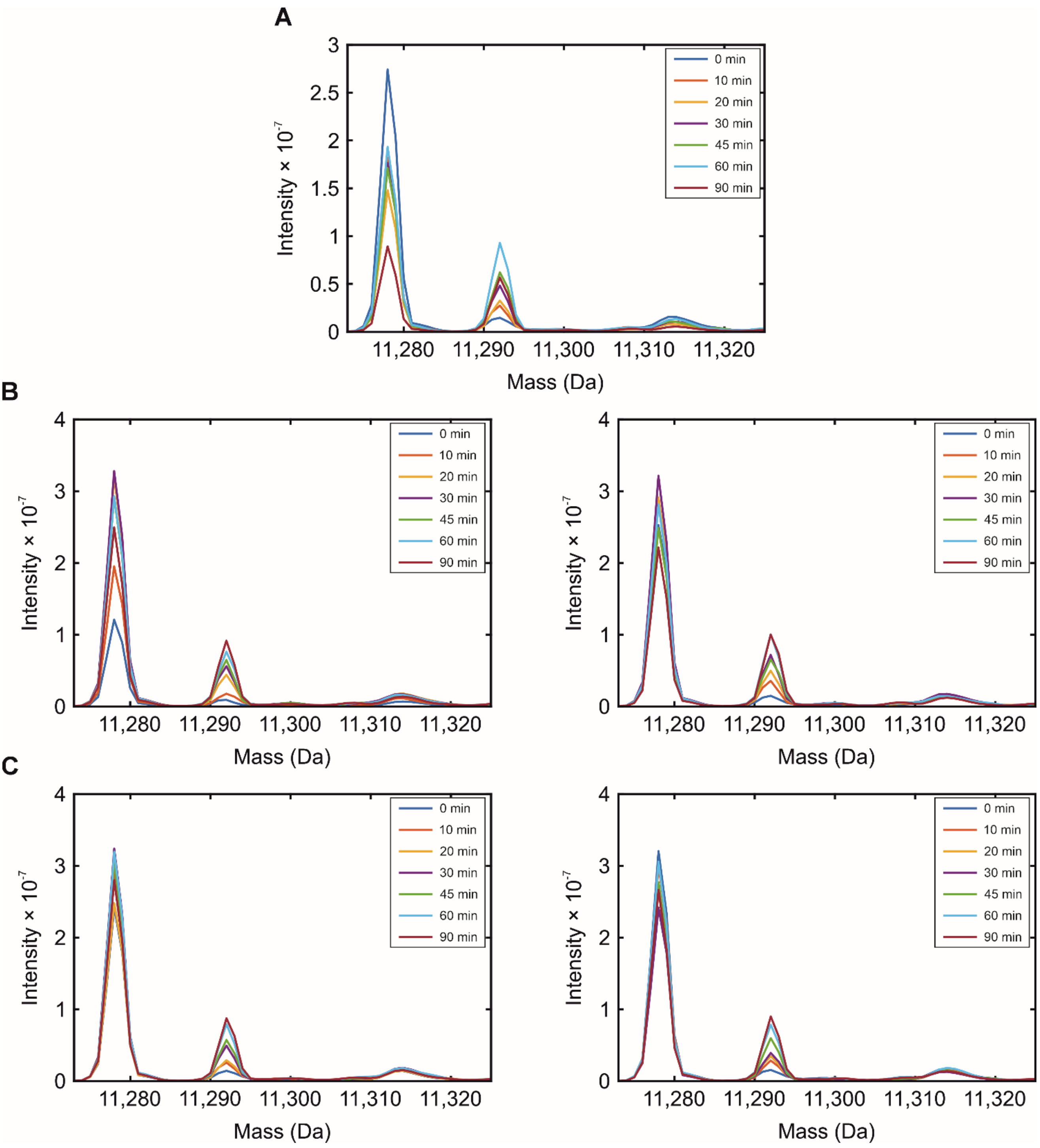

Figure S27. Deconvoluted mass spectra of nucleosome array monomethylation assays.

Deconvoluted mass spectra obtained from the TICs in Figure S26 for every timepoint of 12-mers containing (A) only H4D24-containing octamers or (B) 2:1 and (C) 1:1 ratios of H4D24- and H4isoD24-containing octamers, respectively.

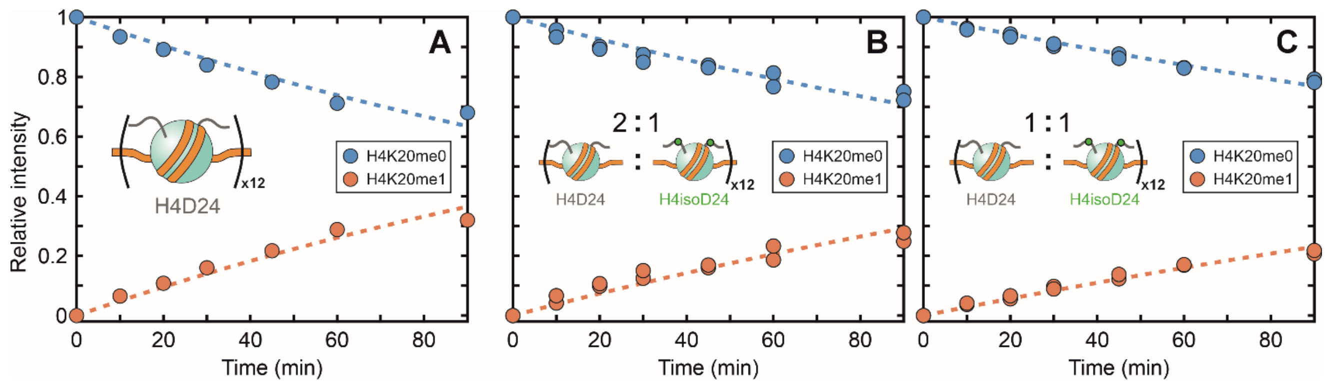

Figure S28. Kinetics of nucleosome array monomethylation assays.

Relative intensities as measured by HRMS of 12-mers assembled using (A) only H4D24-containing octamers or ratios of (B) 2:1 and (C) 1:1 H4D24- and H4isoD24-containing octamers, respectively, and exhibiting H4K20me0 (blue circles) and H4K20me1 (red circles) after incubation with Set8. Dashed lines represent global fits of the data to a single-step reaction model (Equations S8, S11).

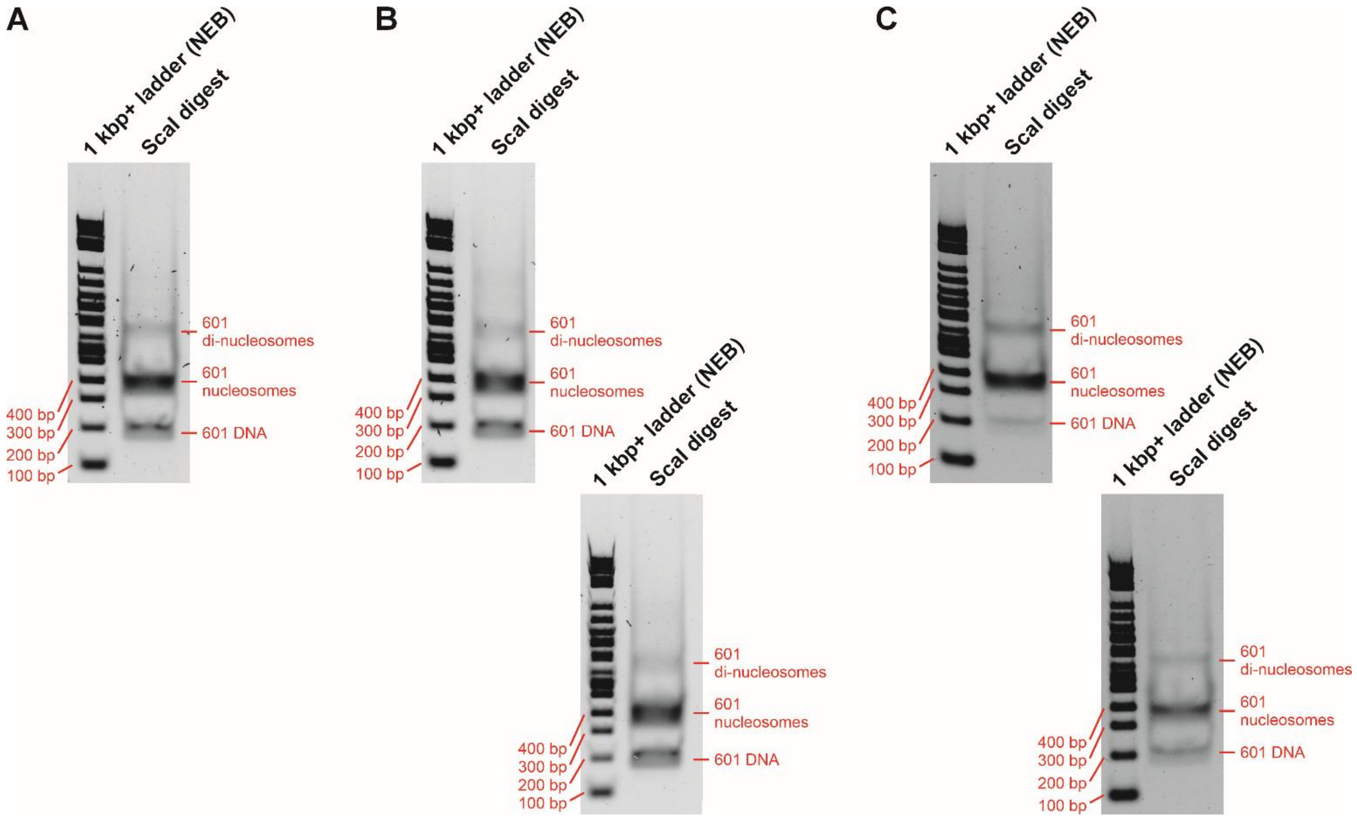

Figure S29. Analysis of nucleosome array integrity after monomethylation assays.

Native APAGE of all replicates of 12-mers assembled using (A) only H4D24-containing octamers or ratios of (B) 2:1 and (C) 1:1 H4D24- and H4isoD24-containing octamers, respectively, and subjected to ScaI restriction digest after Set8 monomethylation assays.

Supplementary References

(1) Kuipers, B. J.; Gruppen, H. Prediction of molar extinction coefficients of proteins and peptides using UV absorption of the constituent amino acids at 214 nm to enable quantitative reverse phase high-performance liquid chromatography-mass spectrometry analysis. *J Agric Food Chem* **2007**, *55* (14), 5445-5451. DOI: 10.1021/jf070337l

(2) Goddard, T. D.; Huang, C. C.; Meng, E. C.; Pettersen, E. F.; Couch, G. S.; Morris, J. H.; Ferrin, T. E. UCSF ChimeraX: Meeting modern challenges in visualization and analysis. *Protein Sci.* **2018**, *27* (1), 14-25. DOI: 10.1002/pro.3235

(3) Southall, S. M.; Cronin, N. B.; Wilson, J. R. A novel route to product specificity in the Suv4-20 family of histone H4K20 methyltransferases. *Nucleic Acids Res.* **2014**, *42* (1), 661-671. DOI: 10.1093/nar/gkt776

(4) Schalch, T. The 30-nm chromatin fiber: In vitro reconstitution and structural analysis. ETH Zürich, Zürich, Switzerland, 2004. https://doi.org/10.3929/ethz-a-004845137 (accessed 2024-08-20).

(5) Zheng, J. S.; Tang, S.; Qi, Y. K.; Wang, Z. P.; Liu, L. Chemical synthesis of proteins using peptide hydrazides as thioester surrogates. *Nat. Protoc.* **2013**, *8* (12), 2483-2495. DOI: 10.1038/nprot.2013.152

(6) Nguyen, U. T.; Bittova, L.; Muller, M. M.; Fierz, B.; David, Y.; Houck-Loomis, B.; Feng, V.; Dann, G. P.; Muir, T. W. Accelerated chromatin biochemistry using DNA-barcoded nucleosome libraries. *Nat. Methods* **2014**, *11* (8), 834-840. DOI: 10.1038/nmeth.3022

(7) Debelouchina, G. T.; Gerecht, K.; Muir, T. W. Ubiquitin utilizes an acidic surface patch to alter chromatin structure. *Nat. Chem. Biol.* **2017**, *13* (1), 105-110. DOI: 10.1038/nchembio.2235

(8) Fierz, B.; Chatterjee, C.; McGinty, R. K.; Bar-Dagan, M.; Raleigh, D. P.; Muir, T. W. Histone H2B ubiquitylation disrupts local and higher-order chromatin compaction. *Nat. Chem. Biol.* **2011**, *7* (2), 113-119. DOI: 10.1038/nchembio.501

(9) Farnung, L.; Vos, S. M.; Wigge, C.; Cramer, P. Nucleosome-Chd1 structure and implications for chromatin remodelling. *Nature* **2017**, *550* (7677), 539-542. DOI: 10.1038/nature24046

(10) Liu, X.; Li, M.; Xia, X.; Li, X.; Chen, Z. Mechanism of chromatin remodelling revealed by the Snf2-nucleosome structure. *Nature* **2017**, *544* (7651), 440-445. DOI: 10.1038/nature22036

(11) Yan, L.; Wu, H.; Li, X.; Gao, N.; Chen, Z. Structures of the ISWI-nucleosome complex reveal a conserved mechanism of chromatin remodeling. *Nat. Struct. Mol. Biol.* **2019**, *26* (4), 258-266. DOI: 10.1038/s41594-019-0199-9

(12) Ho, C. H.; Takizawa, Y.; Kobayashi, W.; Arimura, Y.; Kimura, H.; Kurumizaka, H. Structural basis of nucleosomal histone H4 lysine 20 methylation by SET8 methyltransferase. *Life Sci Alliance* **2021**, *4* (4). DOI: 10.26508/lsa.202000919

(13) Lin, F.; Zhang, R.; Shao, W.; Lei, C.; Ma, M.; Zhang, Y.; Wen, Z.; Li, W. Structural basis of nucleosomal H4K20 recognition and methylation by SUV420H1 methyltransferase. *Cell Discov* **2023**, *9* (1), 120. DOI: 10.1038/s41421-023-00620-5

(14) Weirich, S.; Kudithipudi, S.; Jeltsch, A. Specificity of the SUV4-20H1 and SUV4-20H2 protein lysine methyltransferases and methylation of novel substrates. *J. Mol. Biol.* **2016**, *428* (11), 2344-2358. DOI: 10.1016/j.jmb.2016.04.015

(15) Yin, Y.; Liu, C.; Tsai, S. N.; Zhou, B.; Ngai, S. M.; Zhu, G. SET8 recognizes the sequence RHRK20VLRDN within the N terminus of histone H4 and mono-methylates lysine 20. *J. Biol. Chem.* **2005**, *280* (34), 30025-30031. DOI: 10.1074/jbc.M501691200
